# Subversion of the salicylic acid signaling pathway by the bipartite begomoviral protein BV1 promotes virus infection and vector preference to virus-infected plants

**DOI:** 10.1371/journal.ppat.1014354

**Published:** 2026-07-07

**Authors:** Guan-Ping Chen, Di Li, Xing Zhang, Ming-Yang Yu, Jing-Ru Zhang, Wilmer J. Cuellar, Xiao-Wei Wang, Shu-Sheng Liu, Yin-Quan Liu, Li-Long Pan

**Affiliations:** 1 Ministry of Agriculture and Rural Affairs Key Laboratory of Molecular Biology of Crop Pathogens and Insect Pests, Zhejiang Key Laboratory of Biology and Ecological Regulation of Crop Pathogens and Insects, Institute of Insect Sciences, Zhejiang University, Hangzhou, China; 2 Virology and Crop Protection Laboratory, Cassava Program, International Center for Tropical Agriculture (CIAT), Palmira, Colombia; 3 The Rural Development Academy, Zhejiang University, Hangzhou, China; University of Cambridge, UNITED KINGDOM

## Abstract

The outbreak of vector-borne plant viruses entails both efficient *in planta* virus infection and productive vector-mediated transmission. Yet, the factors that concurrently regulate these two key viral traits remain understudied. Here we examine the role of viral proteins and the salicylic acid (SA) signaling pathway in modulating virus infection and vector preference to virus-infected plants. Infection of plants by the bipartite begomovirus, Sri Lankan cassava mosaic virus (SLCMV), dramatically induces the accumulation of SA, a positive regulator of antiviral defense. As countermeasures, SLCMV DNA-B and the BV1 protein encoded therein interfere with SA-induced antiviral defenses and SA signal transduction. Furthermore, whilst SA induces plant repellence to whitefly vectors, this repellence is mitigated by SLCMV DNA-B and BV1. Mechanistic explorations in *N. benthamiana* plants reveal that BV1 downregulates the transcription of *BTB/POZ and TAZ domain-containing protein 1* (*BT1*), a positive regulator of plant SA signal transduction, antiviral defenses and repellence against whitefly. Finally, these principles of plant-bipartite begomovirus interactions are also documented for another bipartite begomovirus. Together, our data highlight the role of virus-SA interplay in enabling competent interactions among plant hosts, bipartite begomoviruses and their whitefly vectors, and advance our understanding of the molecular mechanisms that promote the persistence of vector-borne plant viruses.

## Author summary

In the field, plant viruses can cause widespread disease epidemics only if they can readily infect and spread among their host plants. Hence, for vector-borne viruses whose spread relies on vectors, dissecting how they interact with their hosts and vectors will unravel the key mechanisms facilitating viral disease outbreaks. While the two processes have been extensively studied, the factors concurrently regulating virus infection and transmission are understudied. Here we explore the modulation of bipartite begomovirus infection and vector preference to infected plants by salicylic acid (SA) signaling pathway and begomoviral BV1 proteins. We show that while virus-induced SA accumulation induces antiviral immunity, DNA-B and BV1 proteins abolish these defenses by interfering with SA signal transduction. Furthermore, whilst SA induces plant repellence to whitefly vectors, this repellence is mitigated by DNA-B and BV1 of bipartite begomoviruses. Mechanistically, BV1 downregulates the transcription of a positive regulator of SA signal transduction. Our data provides critical new insights into the molecular mechanisms promoting the outbreaks of vector-borne plant viruses.

## Introduction

Both wild plants and cultivated crops are constantly challenged by a wide range of diseases caused by pathogenic microorganisms such as viruses. In agricultural ecosystems, viral diseases represent a major threat to the safe production of many staple foods, vegetables, and cash crops worldwide [1]. As obligate parasites, viruses rely on host cellular machinery for their propagation [2]. Over the long-term evolution, many viruses have acquired the ability to be transmitted by arthropod vectors, predominantly hemipteran insects [3]. In the field, vector-mediated transmission dictates the interplant spread of vector-borne viruses and in turn viral disease epidemics [4,5]. Hence, outbreaks of vector-borne plant viruses entail both efficient virus infection in hosts and productive virus transmission by vectors. Identifying the factors and molecular mechanisms that promote virus pathogenesis in plants and transmission by arthropod vectors will provide critical insights into the life cycle of vector-borne plant viruses and in turn viral disease outbreaks.

Upon virus infection of plant hosts, an array of defense responses is activated, including RNA interference and phytohormonal pathways, among others [6–8]. These defense responses significantly modulate multiple aspects of the viral life cycle, including replication and intercellular and long-distance trafficking within hosts [7,8]. As countermeasures, viral pathogens encode versatile proteins that actively subvert antiviral signaling pathways, thereby sustaining *in planta* virus infection (e.g., [9,10]. Among phytohormonal signaling pathways, salicylic acid (SA) plays a key role in plant antiviral defenses [8]. While SA-virus interactions have been studied extensively, progresses are limited for some DNA viruses such as begomoviruses. For example, while in general begomoviruses activate plant SA signaling pathways [11], the viral and plant factors involved are barely known. Additionally, while begomoviruses can be monopartite or bipartite based on the number of genomic molecules and some monopartite begomoviruses are associated with satellites such as betasatellites, so far only viral proteins encoded in monopartite begomoviruses or betasatellites have been reported to subvert SA signaling pathways [7,12–16]. Therefore, more investigations are need to fully dissect the interactions between SA signaling pathway and begomoviruses, particularly bipartite begomoviruses.

During a virus acquisition-transmission cycle, arthropod vectors move to infected plants and acquire viruses during feeding, after which viruliferous vectors translocate to uninfected plants to inoculate the viruses [3]. Productive virus transmission requires compatibility among arthropod vectors, viruses, and plant hosts at each step of this cycle [17]. For example, at the initial step, arthropod vectors must move to infected plants in which active interactions between viruses and plant biological processes have occurred. In this context, vector preference for infected plants, or in other words, the attractiveness of virus-infected plants to vectors, dictates vector movement to the source of inoculum and subsequent virus acquisition and transmission efficiencies [18]. In the last decades, dozens of case studies have been reported on the modulation of whitefly preference by whitefly-borne begomoviruses [14,19–21]. While several studies have been conducted to dissect the plant and viral factors involved, mechanistic insights were provided in only a few studies [14,19,20,22,23]. For example, jasmonate signaling pathway is often modulated by begomoviruses for the manipulation of whitefly preference [14,20,22,23]. Under this scenario, the identification of additional plant and viral factors, and more importantly, the molecular interplays between these factors governing the modulation of whitefly preference by begomoviruses, will significantly expand our understanding of begomovirus-whitefly interactions and virus-vector interactions in general.

Here we examined the impact of virus-SA interplay on bipartite begomovirus infection and vector preference to virus-infected plants. We first investigated the response of plant hormones to the infection by various begomoviruses and begomovirus-betasatellite complexes. We then examined the impact of SA on bipartite begomovirus infection and the viral countermeasures. Next, we explored the modulation of plant repellence against whitefly vectors by SA and viral proteins. Moreover, we deciphered how viral proteins dampen SA signal transduction. Finally, we repeated these analyses using another bipartite begomovirus. Together, our findings unravel a previously unrecognized mechanism that promotes the competent interactions among plant hosts, bipartite begomoviruses and their whitefly vectors.

## Results

### Sri Lankan cassava mosaic virus (SLCMV) induces plant SA accumulation

To explore the interactions between begomoviruses and plant hosts, we analyzed the responses of various phytohormones and related metabolites, including jasmonic acid (JA), jasmonoyl-isoleucine (JA-Ile), 12-oxo-phytodienoic acid (OPDA), abscisic acid (ABA), indole-3-acetic acid (IAA), and salicylic acid (SA), to the infection by diverse begomoviruses. We used two monopartite begomoviruses, including tomato yellow leaf curl virus (TYLCV), papaya leaf curl China virus (PaLCuCNV), and the bipartite begomovirus Sri Lankan cassava mosaic virus (SLCMV). As betasatellites play crucial roles in the life cycles of some monopartite begomoviruses [24], we also included two begomovirus-betasatellite complexes in the analysis, namely cotton leaf curl Multan virus (CLCuMuV)-cotton leaf curl Multan betasatellite (CLCuMuB) and tobacco curly shoot virus (TbCSV)-tobacco curly shoot betasatellite (TbCSB).

**Fig 1:**
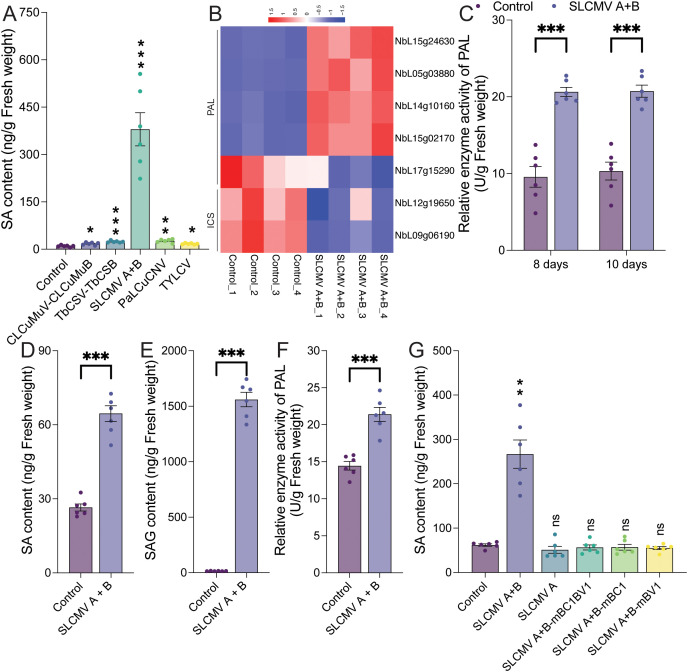
SLCMV induces plant SA accumulation. (A) SA content in *N. benthamiana* plants inoculated with pBINPLUS (control), cotton leaf curl Multan virus (CLCuMuV)-cotton leaf curl Multan betasatellite (CLCuMuB), tobacco curly shoot virus (TbCSV)-tobacco curly shoot betasatellite (TbCSB), Sri Lankan cassava mosaic virus (SLCMV) A + B, papaya leaf curl China virus (PaLCuCNV) or tomato yellow leaf curl virus (TYLCV). n = 6 samples (3 plants per sample). (B) Heatmaps showing the transcription level of *phenylalanine ammonia lyase* (*PAL*) and *isochorismate synthase* (*ICS*) genes that were differentially expressed in the comparisons between SLCMV A + B-infected and control *N. benthamiana* plants. (C) Relative enzyme activity of PAL in control and SLCMV A + B-infected *N. benthamiana* plants. n = 5–6 samples (3 plants per sample). (D and E) The contents of SA (D) and SA 2-O-β-D-glucoside (SAG) (E) in control and SLCMV A + B-infected cassava plants. n = 6 samples (3 plants per sample). (F) Relative enzyme activity of PAL in control and SLCMV A + B-infected cassava plants. n = 6 samples (3 plants per sample). (G) SA content in *N. benthamiana* plants inoculated with SLCMV DNA-A alone or with wild type or mutant DNA-B. n = 6 samples (3 plants per sample). Data were analyzed using the two-sided Student’s t-test and expressed as the mean ± SEM for (A, C, D-G). ns stands for no significant difference, *P < 0.05, **P < 0.01, ***P < 0.001.

Infection of *Nicotiana benthamiana* plants with these begomoviruses or begomovirus-betasatellite complexes resulted in severe leaf curling in apical leaves. Notably, among the viruses or complexes tested, only SLCMV induced leaf yellow mosaic symptoms (S1 Fig). While all begomoviruses and begomovirus-betasatellite complexes induced SA and JA accumulation, only some significantly increased the contents of JA-Ile, OPDA, ABA and IAA (Figs 1A, S2). Notably, SLCMV infection resulted in markedly higher SA accumulation compared with other begomoviruses or begomovirus-betasatellite complexes (Fig 1A). To exclude the effect of agrobacteria infiltration on SA accumulation, we compared SA contents in plants inoculated with infiltration buffer, untransformed agrobacteria and agrobacteria containing pBINPLUS (empty vector). No significant difference in SA contents among the three kinds of plants was found, indicating that neither the agrobacteria nor agrobacteria plus pBINPLUS induces SA accumulation (S3 Fig).

To investigate the molecular basis of SA accumulation, we performed RNA-seq on control and SLCMV-infected *N. benthamiana* plants. In total, 8,892 differentially expressed genes were identified (S4A Fig), and enrichment analysis revealed that several chloroplast-related gene ontology (GO) terms were enriched (S4B Fig). In plants, SA is produced from chorismate via the isochorismate synthase (ICS) and phenylalanine ammonia-lyase (PAL) pathways [25]. We therefore identified *ICS* and *PAL* genes in the *N. benthamiana* genome and examined their transcriptional responses. Of the thirteen *PAL* genes identified, five were differentially expressed, with four being upregulated and one being downregulated, whereas two of the seven identified *ICS* genes were differentially expressed (both were downregulated) (Fig 1B). Consistent with these transcriptional changes, PAL enzyme activity in *N. benthamiana* plants was significantly increased upon SLCMV infection (Fig 1C). To further determine the role of PAL pathways in SLCMV-induced SA accumulation, we employed 2-aminoindan-2-phosphonic acid (AIP), a strong competitive inhibitor of PAL enzymes [26]. In both control and SLCMV-infected plants, AIP induced around 60% reductions in SA content (S5A Fig). These data indicate that PAL pathway contributes to SLCMV-induced SA accumulation in *N. benthamiana* plants.

To determine whether SLCMV induces SA accumulation in its natural host, we analyzed infected cassava (*Manihot esculenta*), in which SLCMV infection caused leaf curling and mosaic symptoms (S6A-S6F Fig). Similar to the observation in *N. benthamiana*, SLCMV infection in cassava significantly increased SA accumulation and, more dramatically, the accumulation of the major SA conjugate SA 2-O-β-D-glucoside (SAG) (Fig 1D-1E). SA accumulation in cassava was also accompanied by a significant increase in PAL enzyme activity (Fig 1F). These findings suggest that the PAL pathway contributes to SLCMV-induced SA accumulation in plants.

We next sought to identify the viral factors responsible for SA accumulation in *N. benthamiana*. SLCMV DNA-A alone was able to systematically infect *N. benthamiana* plants, but DNA-B alone failed to do so [27]. We thus compared SA contents in plants infected with SLCMV DNA-A alone, or with DNA-B (or its mutants). While inoculation with SLCMV DNA-A plus DNA-B (SLCMV A + B) significantly induced SA accumulation, inoculation with SLCMV DNA-A alone did not affect SA content, indicating that the DNA-B component is indispensable for the induction of SA accumulation (Fig 1G). Furthermore, when DNA-A was co-inoculated with mutant DNA-B constructs unable to express BC1 and/or BV1, SA accumulation was abolished, indicating that both BC1 and BV1 are required for the induction of SA accumulation (Fig 1G). Importantly, SLCMV DNA-A accumulation and disease symptoms in *N. benthamiana* were attenuated when DNA-B or its encoded BC1 or BV1 was absent (S7A-S7B Fig). These findings indicate that DNA-B and its encoded BC1 and BV1 are required in SLCMV-induced SA accumulation, likely through their contribution to DNA-A infection and symptom development.

### SLCMV DNA-B and BV1 mitigate SA-induced antiviral defenses by interfering with SA signaling

**Fig 2:**
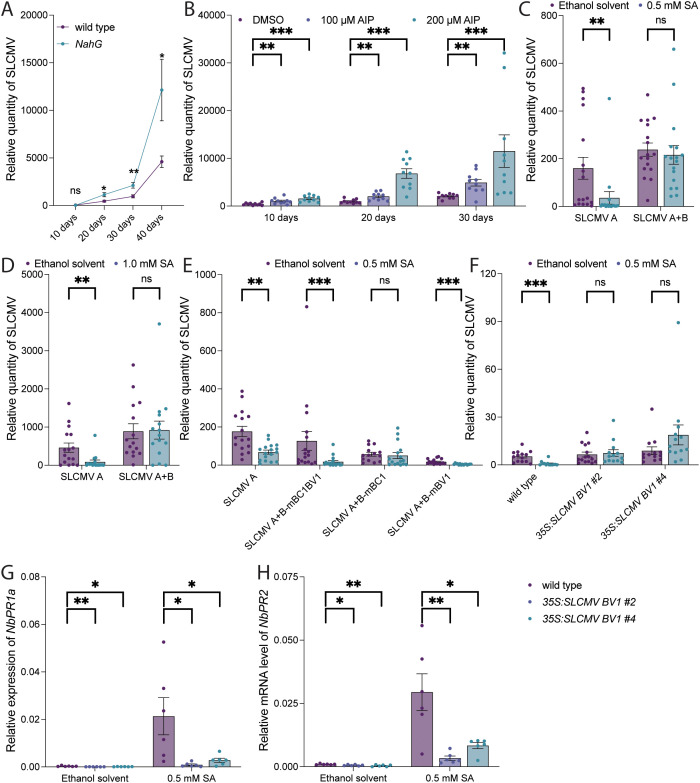
SLCMV DNA-B and BV1 dampen SA-induced antiviral defenses and SA signaling pathway. (A) Relative quantity of SLCMV DNA-A in wild type and *NahG*-transgenic *N. benthamiana* plants that were inoculated with SLCMV DNA-A + DNA-B at 10, 20, 30 and 40 days post inoculation. n = 1–14 plants. (B) Relative quantity of SLCMV DNA-A in *N. benthamiana* plants that were first inoculated with SLCMV DNA-A + DNA-B and then treated with DMSO (solvent control) or AIP. DMSO or AIP were sprayed for three consecutive days at 10 days post virus inoculation, and plants were sampled for virus quantification at 10, 20 and 30 days post the last spray (23, 33 and 43 days post inoculation). n = 15–16 plants. (C and D) Relative quantity of SLCMV DNA-A in *N. benthamiana* plants that were first sprayed with ethanol solvent or SA (C: 0.5 mM, D: 1.0 mM) and then inoculated with SLCMV DNA-A or SLCMV DNA-A + DNA-B. n = 17 plants. (E) Relative quantity of SLCMV DNA-A in plants that were first sprayed with ethanol solvent or 0.5 mM SA and then inoculated with SLCMV DNA-A alone or with various mutant DNA-Bs. n = 14–17 plants. (F) Relative quantity of SLCMV DNA-A in wild type and SLCMV *BV1*-transgenic plants that were first sprayed with ethanol solvent or 0.5 mM SA and then inoculated with SLCMV DNA-A. n = 13–15 plants.(G and H) Relative mRNA level of *PR1a* (G) and *PR2* (H) in wild type and SLCMV *BV1*-transgenic plants. n = 6 samples (3 plants per sample). Data were analyzed using the non-parametric Mann-Whitney U test (A-F) or two-sided Student’s t-test (G-H) and expressed as the mean ± SEM. ns stands for no significant difference, *P < 0.05, **P < 0.01, ***P < 0.001.

To explore the role of the SA signaling pathway in plant defense against SLCMV, we first assessed viral accumulation in *NahG*-transgenic *N. benthamiana* plants, in which SA cannot accumulate. While no significant differences were observed at the early stages of infection (10 days post inoculation), SLCMV DNA-A levels were significantly higher in *NahG*-transgenic plants than in wild type plants at 20, 30 and 40 days post inoculation (Fig 2A). Similarly, pharmacological inhibition of SA biosynthesis using 2-aminoindan-2-phosphonic acid (AIP) resulted in a dose-dependent increase in SLCMV DNA-A titers at 10, 20 and 30 days post treatment (23, 33 and 43 days post inoculation) (Fig 2B). To determine whether the effect of AIP results from its modulation of plant pathways other than SA or the virus itself, we analyzed its effect on the contents of various defense-related hormones and plant antiviral defenses in *NahG*-transgenic *N. benthamiana* plants. AIP treatment did not significantly affect the contents of JA, JA-Ile, OPDA or ABA (S5B-S5E Fig). Additionally, while in wild type plants AIP treatment significantly promoted SLCMV infection, in *NahG*-transgenic plants no significant difference in SLCMV DNA-A titers was found between DMSO and AIP-treated plants (S8 Fig). These results indicate that SA plays an important role in plant defense against SLCMV.

The ability of SLCMV to propagate efficiently in plants with high SA levels suggests that the virus encodes suppressors of SA-mediated immunity. Given that high SLCMV DNA-A titers and SA accumulation occurred concurrently in the presence of DNA-B, we hypothesized that DNA-B plays a role in suppressing SA-mediated defense. *N. benthamiana* plants were treated with SA or ethanol solvent and then inoculated with SLCMV DNA-A alone or with DNA-A plus DNA-B. Exogenous application of 0.5 or 1.0 mM SA substantially reduced DNA-A accumulation when DNA-A was inoculated alone (Fig 2C-2D). In contrast, SA-induced resistance was abolished in plants co-inoculated with DNA-A and DNA-B (Fig 2C-2D). These results demonstrate that SLCMV DNA-B suppresses SA-mediated antiviral defenses.

We further examined the roles of BC1 and BV1 in dampening SA-mediated antiviral defenses. SA treatment significantly suppressed SLCMV accumulation in plants inoculated with SLCMV DNA-A alone (Fig 2E). Similarly, SA induced resistance against DNA-A was observed when DNA-A was co-inoculated with mutant DNA-B constructs defective in BV1 or both BC1 and BV1 expression (Fig 2E). However, SA treatment did not reduce DNA-A titers in plants inoculated with DNA-A and a DNA-B construct defective in BC1 expression (Fig 2E). These results indicate that BV1 is required for the suppression of SA-induced defense by SLCMV DNA-B.

To further corroborate the role of BV1 in suppressing SA-mediated immunity, we generated transgenic *N. benthamiana* lines constitutively expressing SLCMV *BV1* (S9A-S9B Fig). While SA application significantly enhanced resistance to SLCMV DNA-A in wild type plants, SA-induced defenses were abolished in two independent *BV1*-transgenic lines (Fig 2F). To dissect the underlying mechanism, we examined whether BV1 affects SA accumulation or downstream signaling. SLCMV BV1 expression in *N. benthamiana* plants did not significantly alter endogenous SA levels (S10 Fig). However, transcription of the SA-responsive marker genes *PR1a* and *PR2* was significantly reduced in *BV1*-transgenic plants compared with wild type plants (Fig 2G-2H). These data demonstrate that SLCMV BV1 antagonizes SA-mediated antiviral defenses by suppressing SA signaling.

### SLCMV DNA-B and BV1 interfere with SA-induced plant repellence against whitefly

**Fig 3:**
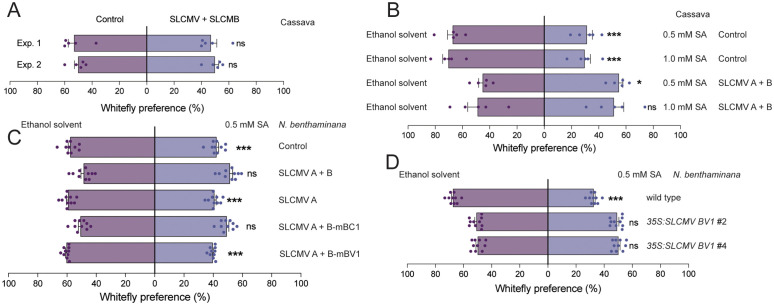
SLCMV DNA-B and BV1 subvert SA-induced plant repellence against whitefly. (A) Percentage of whiteflies choosing control or SLCMV A + B-infected cassava plants. Two plants of different treatments were placed diagonally in a cage, and the distance between the two closest leaves of the two plants was 20 cm. Next, twenty-five whiteflies were released at the center of the cage and 30 mins later the number of whiteflies on each plant was counted. The position of the two plants were then switched and 25 whiteflies were released and later counted again. Data from the two trails were combined as one replicate. n = 5 replicates. (B) Percentage of whiteflies choosing ethanol solvent or SA-treated control and SLCMV A + B-infected cassava plants. Cassava plants were first inoculated with pBINPLUS (control) or SLCMV A + B. The two kinds of plants were then treated with ethanol solvent or SA for three days. At one day post the last spray, whitefly preference to ethanol solvent-treated and SA-treated plants was determined at one day post the last spray. n = 5 replicates. (C) Percentage of whiteflies choosing ethanol solvent or SA-treated *N. benthamiana* plants that were inoculated with pBINPLUS (control), SLCMV A + B, SLCMV A, SLCMV A + B-mBC1 and SLCMV A + B-mBV1. *N. benthamiana* plants were first inoculated with pBINPLUS (control), SLCMV A + B, SLCMV A, SLCMV A + B-mBC1 or SLCMV A + B-mBV1. The five kinds of plants were then treated with ethanol solvent or SA for three days. Whitefly choice assay was conducted at one day post the last spray. n = 10 replicates. (D) Percentage of whiteflies choosing ethanol solvent or SA-treated wild type and SLCMV *BV1*-transgenic *N. benthamiana* plants. n = 10 replicates. Data were analyzed using the Generalized Linear Mixed Model (GLMM) with binomial distribution and logit link function and expressed as the mean ± SEM. ns stands for no significant difference, *P < 0.05, ***P < 0.001.

In addition to their roles in modulating virus infection, SA-mediated responses may also induce plant repellence against the whitefly vectors of begomoviruses (e.g., [28–31]. We thus tested whitefly preference for SLCMV-infected and control cassava plants. Intriguingly, no significant difference in whitefly preference was detected between the two plant types in two independent experiments (Fig 3A). The absence of detectable repellence in SLCMV-infected cassava plants despite high SA levels suggests that SLCMV may interfere with SA-induced plant repellence. To test this hypothesis, control and SLCMV-infected cassava plants were treated with SA or ethanol solvent, and then subjected to whitefly preference assays. While SA treatment (either 0.5 mM or 1.0 mM) significantly induced repellence against whitefly in control cassava plants, in SLCMV-infected cassava plants SA treatments did not affect whitefly preference (1.0 mM) or even rendered plants slightly more attractive (0.5 mM, 52.2% of whiteflies preferred SA-treated plants) (Fig 3B).

Similarly, in control *N. benthamiana* plants, treatment with 0.5 mM SA significantly increased plant repellence to whitefly, whereas in SLCMV A + B-inoculated plants SA-induced repellence was abolished (Fig 3C). Furthermore, while SA induced repellence against whitefly in plants inoculated with DNA-A alone or with DNA-B defective in BV1 expression, SA-induced repellence was abolished in plants inoculated with DNA-A plus DNA-B defective in BC1 expression (Fig 3C). The role of BV1 was further corroborated using *BV1*-transgenic *N. benthamiana* plants. SA treatment rendered wild type plants significantly more repellent to whitefly, but did not significantly affect repellence in two independent *BV1*-transgenic lines (Fig 3D). These data suggest that SLCMV DNA-B and its encoded BV1 interfere with SA-induced plant repellence against whitefly.

### SLCMV BV1 downregulates the transcription of *N. benthamiana BTB/POZ and TAZ domain-containing protein 1* (*NbBT1*), an SA-inducible gene

**Fig 4:**
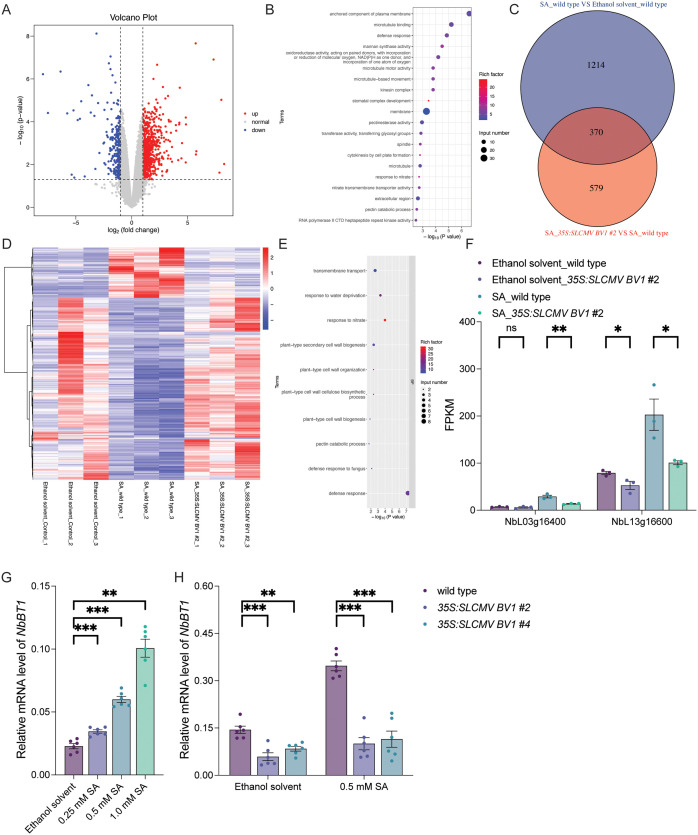
SLCMV BV1 downregulates the transcription of *NbBT1*, a SA-inducible gene. (A and B) Differentially expressed genes in SA-treated SLCMV *BV1*-transgenic *N. benthamiana* plants as compared with SA-treated wild-type plants (A), and GO enrichment analysis of these genes (B). Wild type and SLCMV *BV1*-transgenic *N. benthamiana* plants were treated with 0.5 mM SA and then sampled for RNA-seq. Differentially expressed genes were shown in the volcano plot with upregulated genes marked as red dots and downregulated genes marked as blue dots (A) and the identified genes were subjected to gene ontology (GO) enrichment analysis (B). (C) Venn diagram presenting the genes that were differentially expressed in the comparison between SA-treated wild type and ethanol solvent-treated wild type plants, and in the comparison between SA-treated SLCMV *BV1*-transgenic and SA-treated wild type plants. (D and E) Heatmaps showing the transcription level (D) and GO enrichment (E) of the genes that were differentially expressed in both comparisons. (F) Fragments per kilobase of exon model per million mapped fragments (FPKM) of two transcripts (NbL03g16400 and NbL13g16600) in ethanol solvent-treated wild type plants, ethanol solvent-treated SLCMV *BV1*-transgenic plants, SA-treated wild type plants, and SA-treated SLCMV *BV1*-transgenic plants. n = 3 samples (3 plants per sample) (G) Relative mRNA level of *NbBT1* in wild type *N. benthamiana* plants that were treated with ethanol solvent or 0.25, 0.5 or 1.0 mM SA. n = 6 samples (3 plants per sample). (H) Relative mRNA level of *NbBT1* in wild type and SLCMV *BV1*-transgenic *N. benthamiana* plants that were treated with ethanol solvent or 0.5 mM SA. n = 6 samples (3 plants per sample). Data were analyzed using the two-sided Student’s t-test for F-H and expressed as the mean ± SEM. *P < 0.05, **P < 0.01, ***P < 0.001.

To elucidate the mechanism by which SLCMV BV1 interferes with SA signaling, we performed RNA-seq. In total, four types of *N. benthamiana* plants, namely ethanol solvent-treated wild type, ethanol solvent-treated SLCMV *BV1*-transgenic, SA-treated wild type and SA-treated SLCMV *BV1*-transgenic plants. We hypothesized that the genes mediating the suppression of SA signaling by BV1 may be upregulated by SA treatment and downregulated by BV1. Following this strategy, we conducted comparisons between ethanol solvent- and SA-treated wild type plants, and SA-treated wild type and SA-treated *BV1*-transgenic plants. Comparison between ethanol solvent- and SA-treated wild type plants identified 1,584 differentially expressed genes involved in multiple pathways (S11A-S11B Fig). Comparison between SA-treated wild type and SA-treated *BV1*-transgenic plants identified 949 differentially-expressed genes (Fig 4A), and subsequent enrichment analysis revealed that several GO terms, including defense response were overrepresented (Fig 4B). We then identified 370 genes that were differentially-expressed in both comparisons (Fig 4C) and analyzed their transcriptional profiles (Fig 4D) and associated pathways (Fig 4E).

Because SLCMV BV1 may interfere with SA signaling by modulating SA-induced transcriptional changes of the regulators of SA signaling, we searched for the GO terms related to SA signaling among these 370 genes. We identified the term response to salicylic acid (GO: 0009751). Within this category, only two genes, namely *NbL03g16400* and *NbL13g16600*, were differentially expressed; both encode members of the *BTB/POZ and TAZ domain-containing protein 1* family. Transcript abundance analysis in plants of the four treatments showed that while the transcription of both genes was downregulated by BV1 upon SA treatment, only *NbL13g16600* was downregulated by BV1 in ethanol solvent-treated plants (Fig 4F). We selected *NbL13g16600* for further analysis because of its relatively high transcription level and that it was regulated by BV1 whether SA was sprayed or not. *NbL13g16600* was designated *N. benthamiana BTB/POZ and TAZ domain-containing protein 1* (*NbBT1*).

We next characterized *NbBT1* transcription using qPCR. In wild type plants, SA treatment significantly increased *NbBT1* transcript levels in a dose-dependent manner (Fig 4G). Furthermore, in both ethanol solvent- and SA-treated plants, SLCMV BV1 significantly reduced *NbBT1* transcription (Fig 4H). Together, these results indicate that SLCMV BV1 downregulates the transcription of *NbBT1*, a SA-inducible gene.

### *NbBT1* positively regulates plant SA signaling, antiviral defenses and repellence against whitefly

**Fig 5:**
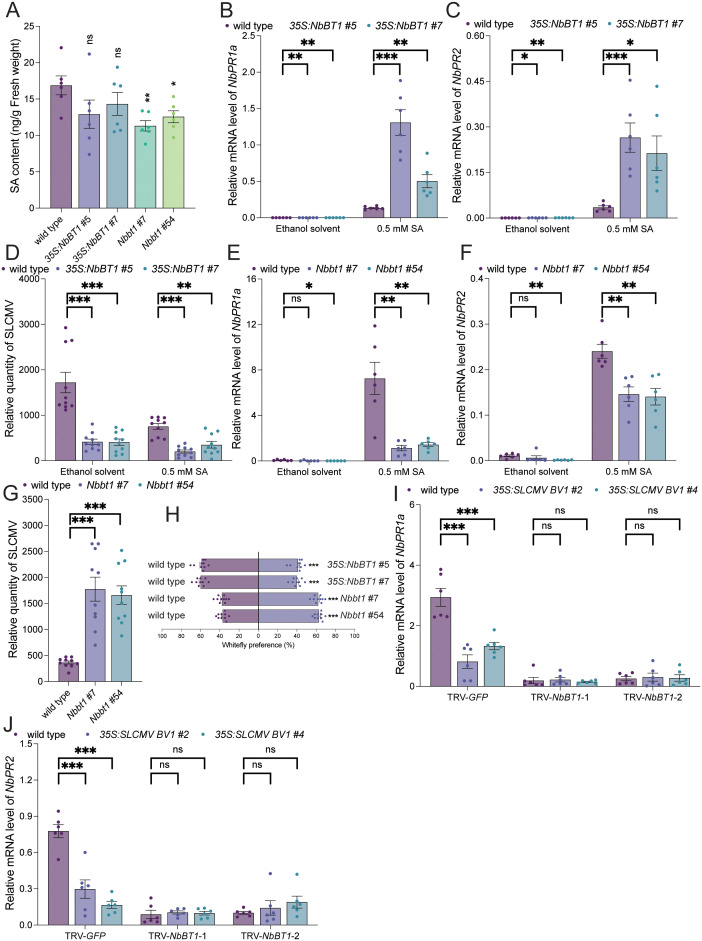
*NbBT1* positively regulates plant SA signaling, antiviral defenses and repellence against whitefly and is required for the suppression of SA signaling by SLCMV BV1. (A) SA content in wild type, *NbBT1-*transgenic and knockout *N. benthamiana* plants. n = 6 samples (3 plants per sample). (B and C) Relative mRNA level of *PR1a* (B) and *PR2* (C) in wild type and *NbBT1-*transgenic *N. benthamiana* plants that were sprayed with ethanol solvent or SA. n = 6 samples (3 plants per sample). (D) Relative quantity of SLCMV DNA-A in wild type and *NbBT1-*transgenic *N. benthamiana* plants that were first sprayed with ethanol solvent or SA and then inoculated with SLCMV DNA-A + DNA-B. n = 10 plants. (E and F) Relative mRNA level of *PR1a* (E) and *PR2* (F) in wild type and *NbBT1-*knockout *N. benthamiana* plants that were sprayed with ethanol solvent or SA. n = 6 samples (3 plants per sample). (G) Relative quantity of SLCMV DNA-A in wild type and *NbBT1-*knockout *N. benthamiana* plants that were inoculated with SLCMV DNA-A + DNA-B. n = 10 plants. (H) Percentage of whiteflies choosing wild type, *NbBT1-*knockout or *NbBT1*-overexpressing *N. benthamiana* plants. n = 10 replicates. (I and J) Relative mRNA level of *PR1a* (I) and *PR2* (J) in wild type and SLCMV *BV1-*transgenic *N. benthamiana* plants that were inoculated with TRV-*GFP*, TRV-*BT1*–1 or TRV-*BT1*–2 and then sprayed with 0.5 mM SA for 3 days at 7 days post inoculation. Plants were sampled for gene transcription analysis at one day post the last spray. n = 6 samples (3 plants per sample). Data were analyzed using the two-sided Student’s t-test (A-C, E-F, I-J), or non-parametric Mann-Whitney U test (D, G), or Generalized Linear Mixed Model (GLMM) with binomial distribution and logit link function (H), and expressed as the mean ± SEM. ns stands for no significant difference, *P < 0.05, **P < 0.01, ***P < 0.001.

To characterize the function of *NbBT1*, we generated and validated two *NbBT1-*overexpression *N. benthamiana* lines and two *NbBT1-*knockout lines (S12A-S12D Fig). While *NbBT1* overexpression did not significantly affect SA content, *NbBT1* knockout slightly reduced SA levels (Fig 5A). *NbBT1* overexpression significantly increased the transcription of *NbPR1a* and *NbPR2* following ethanol solvent or SA treatment (Fig 5B-5C) and significantly enhanced plant resistance to SLCMV, as evidenced by reduced SLCMV DNA-A accumulation (Fig 5D). In contrast, *NbBT1* knockout significantly reduced *NbPR1a* and *NbPR2* transcription and decreased resistance to SLCMV (Fig 5E-5G). In addition, *NbBT1* knockout and overexpression significantly impaired and induced plant repellence against whitefly, respectively, as indicated by the percentage of whiteflies choosing different plants (Fig 5H). Collectively, these data demonstrate that *NbBT1* serves as a positive regulator of plant SA signaling, antiviral defenses, and repellence against whitefly.

### *NbBT1* is required for the suppression of SA signaling by SLCMV BV1

To further examine the role of *NbBT1* in BV1-mediated suppression of SA signaling, we silenced *NbBT1* in wild type and *BV1*-transgenic plants. Gene transcription analysis showed that in all the three kinds of plants, *NbBT1* transcription levels were downregulated by over 70% (S13 Fig). In TRV-*GFP* inoculated plants, transcription of *NbPR1a* and *NbPR2* was significantly lower in *BV1*-trangenic plants than in wild type plants (Fig 5I-5J). However, when *NbBT1* was silenced, no significant difference in *NbPR1a* and *NbPR2* transcript levels was detected between wild type and *BV1*-trangenic plants (Fig 5I-5J). These results indicate that *NbBT1* is required for the suppression of SA signaling by SLCMV BV1.

### SLCCNV induces SA accumulation in plants and SLCCNV BV1 interferes with SA-mediated plant antiviral defenses and repellence against whitefly by subverting SA signaling

**Fig 6:**
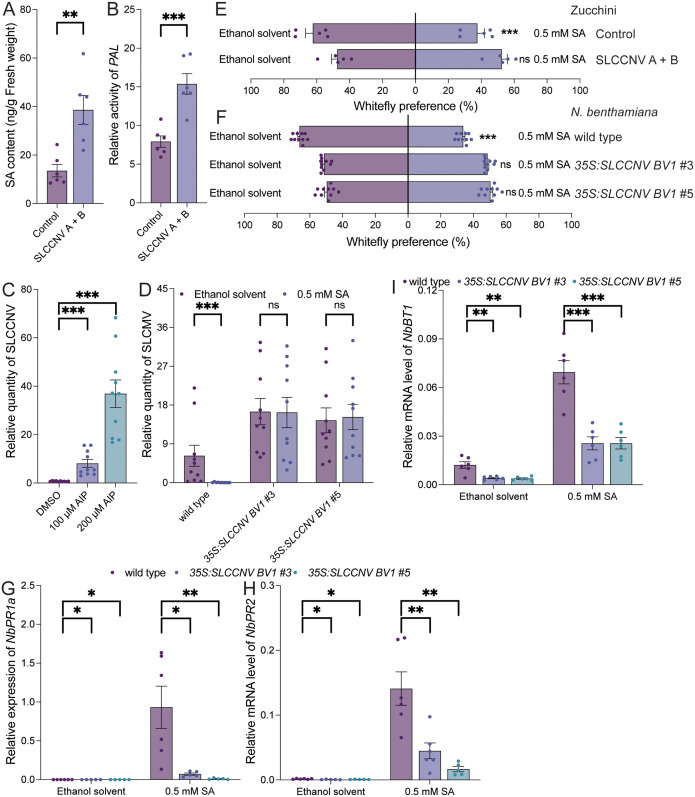
SLCCNV induces plant SA accumulation, and SLCCNV BV1 interferes with SA signaling and downregulates the transcription of *NbBT1.* (A and B) SA content (A) and relative enzyme activity of PAL (B) in control and SLCCNV A + B-infected zucchini plants. n = 6 samples (2–3 plants per sample). (C) Relative quantity of SLCCNV DNA-A in SLCCNV A + B-infected zucchini plants that were treated with DMSO (solvent) or AIP. n = 10 plants. (D) Relative quantity of SLCMV DNA-A in wild type and SLCCNV *BV1-*transgenic *N. benthamiana* plants that were first sprayed with ethanol solvent or SA and then inoculated with SLCMV DNA-A. n = 10 plants. (E) Percentage of whiteflies choosing ethanol solvent or SA-treated control and SLCCNV A + B-infected zucchini plants. n = 5 replicates. (F) Percentage of whiteflies choosing ethanol solvent or SA-treated wild type and SLCCNV *BV1*-transgenic *N. benthamiana* plants. n = 10 replicates. (G and H) Relative mRNA level of *PR1a* (G) and *PR2* (H) in wild type and SLCCNV *BV1-*transgenic *N. benthamiana* plants that were first sprayed with ethanol solvent or SA. n = 6 samples (3 plants per sample). (I) Relative mRNA level of *NbBT1* in wild type and SLCCNV *BV1-*transgenic *N. benthamiana* plants that were first sprayed with ethanol solvent or SA. n = 6 samples (3 plants per sample). Data were analyzed using the two-sided Student’s t-test (A-B, G-I), or non-parametric Mann-Whitney U test (C-D), or Generalized Linear Mixed Model (GLMM) with binomial distribution and logit link function (E-F), and expressed as the mean ± SEM, n. s. stands for no significant difference, *P < 0.05, **P < 0.01, ***P < 0.001.

To determine whether virus-induced SA accumulation and BV1-mediated suppression of SA signaling were only found on SLCMV, we examined squash leaf curl China virus (SLCCNV), another bipartite begomovirus. Similar to SLCMV, SLCCNV infection of zucchini plants significantly increased SA content and PAL enzyme activity (Fig 6A-6B). Pharmacological inhibition of SA biosynthesis using AIP significantly increased SLCCNV DNA-A accumulation (Fig 6C), indicating that SA contributes to antiviral defense in zucchini.

To examine the role of SLCCNV BV1, we generated and validated SLCCNV *BV1*-transgenic *N. benthamiana* plants (S9C-S9D Fig). Because SLCCNV DNA-A alone does not infect *N. benthamiana*, plants were inoculated with SLCMV DNA-A as a proxy for antiviral defense. While SA application significantly reduced SLCMV DNA-A accumulation in wild type plants, SA-induced resistance was abolished in SLCCNV *BV1*-transgenic plants (Fig 6D).

We next investigated the effects of SA and SLCCNV BV1 on plant repellence against whitefly. In control zucchini plants, SA treatment significantly induced repellence against whitefly (Fig 6E). In contrast, in SLCCNV-infected zucchini plants, no significant difference in whitefly preference was observed between ethanol solvent and SA-treated plants (Fig 6E). Moreover, SA-induced repellence against whitefly was abolished in SLCCNV *BV1*-transgenic *N. benthamiana* plants (Fig 6F).

Analysis of SA marker gene expression revealed that SLCCNV *BV1-*transgene significantly reduced *NbPR1a* and *NbPR2* transcript levels (Fig 6G-6H). In addition, SLCCNV BV1 downregulated the transcription of *NbBT1* following ethanol solvent or SA treatment (Fig 6I).

Taken together, these data demonstrate that SLCCNV induces SA accumulation and that SLCCNV BV1 dampens SA-mediated plant antiviral defenses, repellence against whitefly, and SA signaling by downregulating the transcription of *NbBT1*.

## Discussion

**Fig 7:**
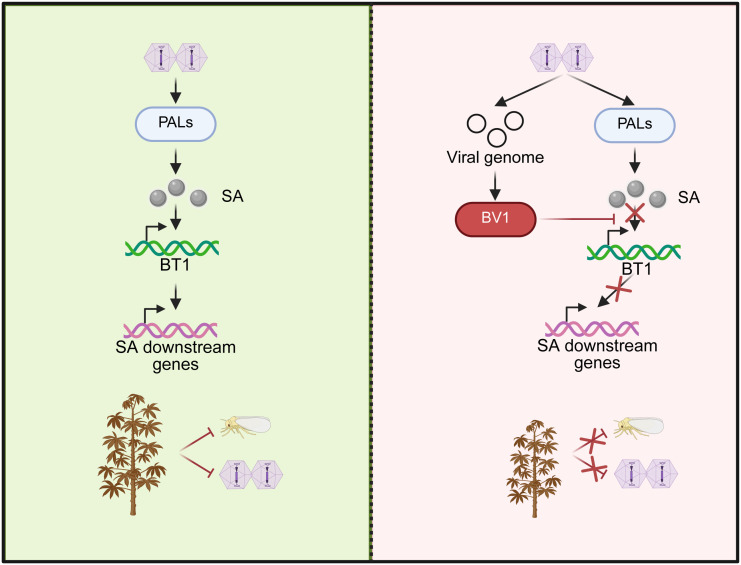
A working model for the role of viral proteins and SA signaling pathway in orchestrating bipartite begomovirus infection and vector preference. Infection of bipartite begomoviruses induces the transcription of *PAL* genes and in turn SA accumulation. SA upregulates the transcription of *NbBT1*, thereby activating the transcription of SA downstream genes and in turn plant antiviral defenses and repellence against whitefly vectors (left panel). BV1 proteins encoded in bipartite viruses interfere with SA-induced transcription of *NbBT1*, thereby mitigating SA-induced plant antiviral defenses and repellence against whitefly (right panel).

During virus infection, plants mount a repertoire of antiviral defenses that may be actively targeted by viral proteins to promote viral propagation. Concurrently, these virus-plant defense interactions may impact the life history of other plant-associated organisms, such as insect vectors. Notably, empirical studies examining these interactions and their underlying molecular mechanisms remain limited. Using multiple bipartite begomovirus-plant pathosystems, we show that virus infection and plant repellence against insect vectors are jointly modulated by SA signaling pathway and viral proteins. Upon infection by two bipartite begomoviruses, the PAL pathway contributes to SA accumulates in host plants. SA then activates the transcription of *NbBT1* and downstream SA-responsive genes, leading to enhanced antiviral defenses and repellence against whitefly. BV1 proteins encoded by the DNA-B components of two bipartite begomoviruses interfere with SA-mediated antiviral defenses and plant repellence by dampening SA signaling through downregulating *NbBT1* transcription (Fig 7).

Upon the infection by viral pathogens, a repertoire of plant defense responses was activated, including RNA interference and phytohormonal pathways, among others [6–8]. Here we focus on phytohormones as they are key regulators of plant development and stress responses, and their responses to begomovirus infection are understudied [32]. We profiled the contents of various hormones and related metabolites in pBINPLUS-inoculated (control) and begomoviruses or begomovirus-betasatellite complexes-infected *N. benthamiana* plants that were obtained using the same agrobacterium strain, inoculum OD value and vector backbone (pBINPLUS). SA accumulation was induced by all tested viruses and complexes, with the most pronounced increase (over 37 folds) observed following infection with the bipartite begomovirus SLCMV. The disparity in inducing SA accumulation might to be attributable to several reasons such as differential virus accumulation and symptoms, and should be subjected to further analysis. SA accumulation has been reported in several plant-begomovirus pathosystems, including pepper-euphorbia mosaic virus and tomato-tomato yellow leaf curl virus [33–36]. Furthermore, abolishing SA accumulation using *NahG*-transgenic plants or AIP treatment dramatically increased SLCMV infection. Together, these findings indicate that SA signaling pathway plays a conserved and important role in plant responses to begomoviruses, particularly the two bipartite begomoviruses tested in this study. Future studies may explore how SA accumulation is triggered by begomovirus infection, thereby providing new insights into viral activation of this canonical antiviral pathway. Moreover, in addition to phytohormonal pathways, other pathways may also play important roles in modulating the replication and accumulation of bipartite begomoviruses in the plants, and thus should be subjected to further investigations.

While SLCMV DNA-A alone can readily infect *N. benthamiana* plants, DNA-B alone fails to do so [27]. We thus examined the roles of DNA-B and its encoded proteins in SLCMV-induced SA accumulation by coupling DNA-A with DNA-B or its mutants. We show that DNA-B and its encoded proteins BC1 and BV1 were required for the induction of SA accumulation. Concurrently, infection with SLCMV DNA-A plus DNA-B resulted in severe downward leaf curling and leaf yellow mosaic, whereas the absence of DNA-B or mutation of BC1 and/or BC1 significantly attenuated symptoms to mild upward leaf curling. Moreover, absence of DNA-B, BC1 or BC1 significantly reduced the accumulation of SLCMV DNA-A. Given that BC1 and BV1 coordinate intercellular and intracellular movement of viral DNA, respectively [37], our findings suggest DNA-B and its encoded proteins may facilitate SLCMV DNA-A infection by promoting viral movement, thereby indirectly contributing to SA accumulation. At the plant level, transcription of *PAL* but not *ICS1* genes, together with PAL enzyme activity, was induced upon infection of SLCMV DNA-A plus DNA-B. More importantly, inhibiting PAL pathways using inhibitors significantly decreased SLCMV-induced SA accumulation. These results indicate that PAL pathway contributes to SLCMV-induced SA accumulation. This contrasts with findings in *Arabidopsis thaliana*, where SA accumulation induced by bacterial pathogens relies primarily on *ICS* rather than *PAL* genes [38,39]. Further investigations are needed to elucidate the interplay between viral and plant factors that lead to SA accumulation during bipartite begomovirus infection.

While previous studies have reported begomoviral subversion of SA signaling pathway, these studies were conducted with monopartite begomoviruses [13,14,40], or betasatellites associated with monopartite begomoviruses [12,15,16]. Specifically, C2 and C4 encoded by tomato yellow leaf curl virus dampen SA signaling and biosynthesis, respectively [13,20,40]. In addition, βC1 proteins encoded by betasatellites associated with monopartite begomoviruses interfere with SA signaling [12,15,16]. The genomic organization of bipartite begomoviruses differ significantly from their monopartite counterparts, and more importantly DNA-B of bipartite begomoviruses share no homology with the genome of monopartite begomoviruses or betasatellites except in the intergenic region [41]. How bipartite begomoviruses interact with host SA signaling pathway remain largely unknown. In this study, we unravel the dramatic induction of SA accumulation by two bipartite begomoviruses and modulation of SA-mediated plant antiviral defenses and repellence against whitefly, and SA signaling by DNA-B and DNA-B-encoded BV1 proteins. When exploring the modulation of SA-mediated antiviral immunity by SLCCNV BV1, we used SLCMV DNA-A as a proxy for SLCCNV DNA-A. Since the two viruses may differ in interactions with plant host, further investigations are required to further clarify the role of SLCCNV BV1. Nevertheless, our findings clearly show that BV1 proteins encoded by DNA-B of two bipartite begomoviruses subverts SA signaling. It should be noted that in our study all functional characterizations of BV1 proteins were conducted in *N. benthamiana*, but not the natural hosts of bipartite begomoviruses. Dissecting the role of BV1 in natural host plants such as cassava and zucchini will further extend our understanding of this important begomoviral protein.

In this study, we uncover two seemingly contrasting roles of BV1 proteins in SLCMV interaction with plant SA signaling pathway. On the one hand, BV1 is required for the induction of SA accumulation by SLCMV as mutation of the ORF encoding this gene abolished SLCMV-induced SA accumulation and at the same time significantly impaired SLCMV DNA-A accumulation and symptom development. On the other hand, BV1 subverts SA signaling as it markedly suppressed the transcription of SA downstream genes while does not affect SA content in plants. We propose that the two roles of BV1 are not conflicting as they occur in different processes. During SLCMV infection, BV1 may mediate the nuclear export of viral DNAs [37], and in turn promote virus systematic infection and symptom development, thereby indirectly contributing to SA accumulation. Concurrently, BV1 suppresses the downstream signaling of SA, thereby sustaining virus infection and plant attractiveness to whitefly. In addition to acting as a suppressor of SA signaling, BV1 proteins have also been shown to suppress transmembrane receptor kinase-mediated antiviral defenses, post-transcriptional gene silencing, and jasmonate signaling [22,42,43]. It seems that BV1 is similar to βC1 encoded in betasatellites associated with begomovirus, which promotes virus infection and at the same time dampens multiple plant antiviral defenses [44]. The identification of BV1 as a suppressor of SA signaling further expands our understanding of the multifaceted roles of bipartite begomoviral proteins in counteracting plant antiviral defenses.

So far, only three plant factors have been implicated in the modulation of whitefly preference by begomoviruses. For example, begomoviruses and begomoviral proteins modify whitefly preference by disrupting plant JA signaling [14,22,23] or inducing β-myrcene production and release [19]. Recently, AC1 proteins encoded in two SLCMV isolates (Col and HN7) induce plant repellence against whiteflies by promoting the accumulation and dimerization of MYC2 [20]. Therefore, our understanding of the modulation of whitefly preference by begomoviruses is far from complete. Here we found that SLCMV infection dramatically induces SA accumulation. Considering that the activation of SA signaling pathway in several plant species, including tomato, cucumber, and tobacco, induces repellence against whitefly [28–31,45], we explore the role of SA signaling pathway in the modulation of whitefly preference by begomoviruses. We found that although SLCMV infection in cassava and SLCCNV infection in zucchini resulted in substantial SA accumulation, no whitefly repellence was observed. It should be noted that while SLCMV of the two isolates Col and HN7 induces the repellence against whitefly in cassava and *N. benthamiana* plants in Wang D et al. [20], no such repellence was found in our study. While further explorations are need to determine the reason for the divergence, we propose that sequence similarity may play a key role. The sequence similarities between our isolate (Combodia2015) and HN7 are 99.82% for DNA-A and 99.78% for DNA-B, and that between Combodia2015 and Col are 93.33% for DNA-A, 95.69% for DNA-B. The more closely-related isolate HN7 induces only marginal repellence (45.4% whiteflies preferred infected plants) while the distantly-related isolate Col induces much more obvious repellence (only 26.8% whiteflies preferred infected plants).

The fact that the infection by two bipartite begomoviruses in plants dramatically induces SA accumulation but did not affect plant repellence against whitefly urges us to explore the modulation of SA-mediated repellence by these viruses. In SA application experiments we found that BV1 proteins encoded by DNA-B interfered with SA-induced repellence against whitefly that was observed in wild type plants. These findings indicate that while virus-induced SA accumulation has the potential to deter whitefly, the two bipartite begomoviruses actively counteract this effect through BV1, thereby maintaining the attractiveness of infected plants to vectors. Mathematical modeling suggests that, under most circumstances, increased preference of uninfected vector for virus-infected plants enhances virus transmission [18]. Accordingly, BV1-mediated maintenance of whitefly preference for infected plants is likely to promote the transmission of bipartite begomoviruses to new hosts. When combining our findings with previous reports [20], we propose a network of bipartite begomovirus-plant-whitefly interactions. On the one hand, AC1 and bipartite begomovirus-induced SA accumulation activate plant repellence against whitefly. Since abundant SA accumulates in virus-infected plants, the function of AC1 may be masked by SA. Concurrently, BV1 interferes with SA-induced plant repellence against whitefly, sustaining whitefly preference to virus-infected plants. It is also possible that the modulation of plant attractiveness to whitefly by one viral protein may be affected by the other, and should be examined in future investigations.

In the attempt to identify plant genes whose transcription is upregulated by SA and downregulated by BV1 using transcriptomic analysis, two gene belonging to the *BTB/POZ and TAZ domain-containing protein 1* family in the GO term response to salicylic acid were identified. Of the two genes, *NbL13g16600* exhibited much higher transcription than *NbL03g16400*. Moreover, while *NbL13g16600* transcription was downregulated by BV1 whether SA was sprayed or not, *NbL03g16400* transcription was downregulated only in SA-treated plants. We choose *NbL13g16600* (renamed as *NbBT1*) for further analysis as it is more likely to function in the suppression of SA signaling by BV1, which dampens SA signaling in both ethanol solvent and SA-sprayed plants. Further qPCR experiments validate that *NbBT1* transcription was upregulated by SA and downregulated by BV1. Although we focus on *NbBT1*, it should be noted that *NbL03g16400* may also play a role and should be examined in future investigations. More importantly, how BV1 modulates *NbBT1* transcription remains unknown, and whether the two BV1 proteins modulate *NbBT1* transcription via the same pathway is currently unknown. Resolving these issues via detailed elucidation of the modulation of *NbBT1* transcription by BV1 proteins will unravel one of the key modes of action of BV1 in reprograming plant physiology. Specifically, the promoter of *NbBT1* and plant proteins dictating the promoter activity such as transcription factors may be identified first, and their molecular interplays with BV1 proteins can be determined.

The BTB/POZ domain is a well-established substrate receptor for Cullin3-based E3 ubiquitin ligases [46], enabling BTB/POZ domain-containing proteins to interact with diverse targets as part of E3 ligase complexes [e.g., 47]. TAZ domains, also known as transcription adaptor putative zinc finger domains, mediate the binding of TAZ domain-containing proteins to transcription factors [48]. In plants, BTB/POZ and TAZ domain-containing proteins regulate diverse biological processes, including development and responses to abiotic and biotic stresses [49–51]. Notably, *A. thaliana BTB/POZ and TAZ domain-containing protein 4* is SA-inducible and mediates SA-dependent resistance to bacterial pathogens [52]. Here, we provide comprehensive evidence that *BT1* in *N. benthamiana* plants is SA-inducible and positively regulates plant SA signaling, antiviral defenses, and repellence against whitefly. These findings broaden our understanding of the roles of BTB/POZ and TAZ domain-containing proteins in plant SA signal transduction and plant interactions with viruses and their insect vectors. However, the precise molecular mechanism by which *BT1* regulates SA signaling remains unknown. More importantly, whether *BT1* in the natural hosts of bipartite begomoviruses play a similar role has not been determined. Future investigations should explore the function of *BT1* in cassava and zucchini plants, and the mode of action of BT1 proteins in regulating SA signaling.

In summary, we reveal how the infection of two bipartite begomoviruses and whitefly preference are jointly modulated by the interplay between the SA signaling pathway and BV1 proteins. While SA accumulation induced by the two bipartite begomoviruses activates plant antiviral immunity and repellence against whitefly, BV1 proteins encoded by DNA-B dampen these responses by subverting SA signaling. Mechanistically, BV1 proteins downregulate the transcription of *NbBT1*, a positive regulator of SA signaling. These findings uncover new regulatory factors governing the life cycle of vector-borne plant viruses and reveal a mechanism that facilitates the persistence of bipartite begomoviruses.

## Materials and methods

### Plants

Four plant species were used: cotton (*Gossypium hirsutum* cv. Zhemian 1793), cassava (*Manihot esculenta* cv. SC8), *Nicotiana benthamiana* (laboratory strain), and zucchini (*Cucurbita pepo* cv. Faguodongkui). Cotton plants were grown in an insect-proof greenhouse under natural lighting and controlled temperature at 25 ± 3 °C, and were used for whitefly rearing. Cassava, *N. benthamiana*, and zucchini plants were grown in an insect-proof climate chamber at 26 ± 2 °C, 60–80% relative humidity, and a 14/10 h light/dark photoperiod (light intensity, 200 μmol m^-2^ s^-1^).

SLCMV *BV1* (GenBank accession code: OK571386) and *NbBT1* (NbL13g16600) were ligated into the pBWA(V) HS-3xFlag vector and transgenic *N. benthamiana* plants were generated using agrobacterium (strain GV3101)-mediated transformation by Biorun Co., Ltd. (China). *NbBT1* overexpression was validated with quantitative polymerase chain reaction (qPCR) using primers listed in S1 Table. *NbBT1*-knockout *N. benthamiana* plants (*Nbbt1*) were generated by Biorun Co., Ltd. (China) using the CRISPR/Cas9 technique. Guide RNAs were designed with an online tool (https://crispr.cos.uni-heidelberg.de) and ligated into K5-KRSN-ccdB vector for agrobacterium (strain GV3101)-mediated transformation. The K5-KRSN-ccdB vector employs a plant-codon-optimized *Streptococcus pyogenes* Cas9 variant under the control of the CaMV 35S promoter. T1 plants containing stable and non-mosaic homozygous mutations were used for experimentation after Sanger sequencing of PCR-amplified target regions of *NbBT1* in the plants of T0 and T1 generations. *NahG*-transgenic *N. benthamiana* plants were provided by Dr. Xinzhong Cai (Institute of Biotechnology, Zhejiang University).

### Viruses and agrobacteria-mediated inoculation

The Sri Lankan cassava mosaic virus (SLCMV) isolate Cambodia2015, squash leaf curl China virus (SLCCNV) isolate Guangxi2017, cotton leaf curl Multan virus (CLCuMuV) isolate GD37 with its cognate betasatellite (CLCuMuB), tobacco curly shoot virus (TbCSV) with its cognate betasatellite (TbCSB), tomato yellow leaf curl virus (TYLCV) isolate SH2 and papaya leaf curl China virus (PaLCuCNV) isolate HeNZM1 were used. GenBank accession codes are OK571385 for SLCMV DNA-A, OK571386 for SLCMV DNA-B, MG525551 for SLCCNV DNA-A, MG525552 for SLCCNV DNA-B, JN968573 for CLCuMuV, JN968574 for CLCuMuB, AJ420318 for TbCSV, AJ421484 for TbCSB, AM282874 for TYLCV, and FN256260 for PaLCuCNV. Infectious clones of SLCMV and SLCCNV were constructed in our laboratory and those of CLCuMuV, CLCuMuB, TbCSV, TbCSB, TYLCV and PaLCuCNV were kindly provided by Dr. Xueping Zhou (Institute of Biotechnology, Zhejiang University). All infectious clones were constructed using pBINPLUS as the vector backbone and then mobilized into agrobacteria strain EHA105.

To generate infectious clones of mutant SLCMV DNA-B constructs, the full-length sequence of SLCMV DNA-B was cloned into the pGEM-T Easy Vector (Promega, USA) using primers listed in S1 Table. Site-directed mutagenesis was performed to convert the start codons (ATG) of *BC1* and *BV1* into stop codons (TAG) using Fast Mutagenesis System (Transgen Biotech, China) with primers listed in S1 Table. Full-length sequences were then used to construct infectious clones using pBINPLUS as the vector backbone as previously described [53].

Virus-infected plants were obtained using agrobacteria-mediated inoculation. Agrobacterium cultures containing the empty vector pBINPLUS, infectious clones of SLCMV or SLCCNV DNA-A and DNA-B, CLCuMuV, CLCuMuB, TbCSV, TbCSB, TYLCV and PaLCuCNV were grown separately to an OD_600_ of 2.0 and resuspended in infiltration buffer (10 mM MgCl_2_, 10 mM MES, and 200 µM acetosyringone). DNA-A was inoculated into plants either alone or in a 1:1 ratio with wild type or mutant DNA-B constructs. The final OD_600_ of agrobacteria containing infectious clones of DNA-As was kept constant (1.0) between DNA-A and DNA-A + DNA-B agrobacteria solutions. Infectious clones of CLCuMuV + CLCuMuB and TbCSV-TbCSB were prepared similarly. As for pBINPLUS, TYLCV and PaLCuCNV, the final OD_600_ of agrobacteria was set at 1.0. Inoculation of agrobacterial solutions in cassava and *N. benthamiana* plants was conducted using 1 mL syringes at the 4–6 and 5–6 true-leaf stages, respectively. Cassava plants were inoculated three times (once every 5 days) and analyzed at 45 days post inoculation. *N. benthamiana* plants were inoculated once and used at 10 days post inoculation. Zucchini plants were inoculated once at one true-leaf stage and analyzed at 25 days post inoculation. Control plants were inoculated with agrobacteria containing the empty vector pBINPLUS.

### Analysis of plant hormone contents

Cassava plants at 45 days post inoculation, *N. benthamiana* plants at 10 days post inoculation, and zucchini plants at 25 days post inoculation were used for hormone analysis. For virus-infected plants, only symptomatic plants were used. Apical leaves were harvested and stored at -80 °C until use. To extract plant hormones, leaves were ground in liquid nitrogen, and approximately 0.3 g of powder was mixed with 1 mL ethyl acetate containing 200 ng of D5-IAA (Cat #0311531), D4-SA (Cat #0376581), D6-JA (Cat # 0142821) and D6-JA-Ile (Cat # 0146861) (OlhemIm, Czechoslovakia). Samples were vortexed for 15 min and centrifuged at 4 °C, and supernatants were collected. The residual pellets were subjected to a second extraction with ethyl acetate (0.5 mL), followed by centrifugation. Supernatants from both extractions were mixed, and ethyl acetate was evaporated at 30 °C using a vacuum concentrator (Eppendorf, USA). Residues were resuspended in 0.5 mL of 70% (v/v) methanol through vortexing for 15 min. After centrifugation, supernatants were collected and subjected to detection of plant hormones using HPLC-MS/MS (LCMS-8040, Shimadzu, Japan) equipped with a Shim-pack XR-ODS III column (2.0 mm I.D. × 75 mm L, 1.6 μm particle size; Shimadzu).

### Analysis of viral accumulation and gene transcription

The first fully expanded apical leaves were harvested for analysis of viral accumulation and gene transcription. Genomic DNA was extracted using Easy Plant Genomic DNA Extraction Kit (Cat # DR0302250, Easy-Do, China). Total RNA was extracted using an RNA extraction kit (Cat #AG21024, Accurate Biology, China) following the user manual, and cDNA was synthesized using an Evo M-MLV RT Kit with gDNA Clean for qPCR (Cat #AG11711, Accurate Biology, China). Quantitative real-time PCR was performed using SYBR Green Premix Pro Taq HS qPCR Kit (Cat # AG11701, Accurate Biology, China) on a CFX96 Real-Time PCR Detection System (Bio-Rad, USA). Primers are listed in S1 Table.

### Analysis of phenylalanine ammonia-lyase (PAL) enzyme activity

The first to third apical leaves were harvested and stored at -80 °C. PAL enzyme activity was measured using Phenylalanine Ammonia-lyase (PAL) Activity Assay Kit (UV Colorimetric Method; Cat #D799599, Sangon Biotech, China) as per the user manual. Blank controls that were used to eliminate background absorbance were provided within the kit and used as per the manual. Prior to formal analysis, to ensure the assay was reliable for the plant species tested (*N. benthamiana*, cassava, and zucchini), we first prepared leaf extracts from control and virus-infected plants of three species and made serial dilutions. After treatment following the kit manual, we analyze the absorbance at 290 nm and found that results of the extracts from 0.1 g of leaves were within the linear range of this assay. In the formal analysis, leaves were ground to fine powder, and approximately 0.1 g of tissue was mixed with 1 mL of extraction buffer. After centrifugation at 4 °C, supernatants were mixed with assay reagents and incubated at 30 °C for 30 min. Absorbance at 290 nm was then measured. PAL activity was calculated based on fresh tissue weight, with one unit (U) defined as the amount of enzyme that causes an absorbance change of 0.1 per minute per gram of tissue in a 1 mL reaction system.

### Salicylic acid (SA) and 2-aminoindan-2-phosphonic acid (AIP) treatments

A 2 M stock solution of SA (Cat #247588, Sigma-Aldrich, USA) was prepared using ethanol as solvent. SA stock solutions were diluted with water to obtain working SA solutions at 0.25, 0.5, and 1.0 mM, each containing 0.1% ethanol. An ethanol solution (0.1%) was used as control. Plants were sprayed with approximately 0.5 mL SA or ethanol solution once per day for three consecutive days. One day after the final spray, plants were sampled for gene transcription analysis or subjected to virus inoculation.

AIP (Cat # HY-W004494, MedChemExpress, USA) was dissolved in DMSO to prepare a stock solution (400 mM). Working AIP solutions (100 and 200 μM) were prepared by diluting the stock solution with water, with the final DMSO concentration adjusted to 0.5%. A DMSO solution (0.5%) was used as a control. Plants were sprayed with approximately 0.5 mL of AIP or DMSO solution per plant per day for three consecutive days using a hand sprayer. Virus-infected *N. benthamiana* and zucchini plants were treated with AIP at 10 and 25 days post inoculation, respectively. Samples were collected for virus quantity analysis at 10, 20 and 30 days post the final spray for *N. benthamiana*, and at 5 days post treatment for zucchini plants.

### Whitefly rearing and preference assay

A culture of Middle East-Asia Minor 1 (MEAM1) whiteflies (mt*COI* GenBank accession code: KM821540) was reared on cotton plants. Newly emerged (0–4 days) whiteflies were used for preference assays, which were conducted as described by Li et al. with minor modifications [54]. Two plants representing different treatments were placed diagonally in a cage in an artificial climate chamber, with a distance of 20 cm between the closest leaves of the two plants. Twenty-five whiteflies (0–4 days post emergence) were captured, chilled on ice for 30 s, and then released at the center of the cage. The number of whiteflies on each plant was counted at 30 min post release. The positions of the two plants were then switched, and the assay was repeated. Data from the two trials were combined as one biological replicate.

### Virus-induced gene silencing (VIGS)

For VIGS of *NbBT1*, fragments of approximately 300 bp from CDS (69–368 and 582–881) were cloned into pTRV2 vector using primers listed in S1 Table. Recombinant pTRV2 constructs were introduced into *Agrobacterium tumefaciens* strain EHA105 using electroporation. Agrobacterium cultures containing recombinant pTRV2 or pTRV1 were grown, resuspended, and adjusted to an OD_600_ of 0.2 with resuspension buffer. Agrobacterium cultures harboring pTRV1 and recombinant pTRV2 were mixed in a 1:1 volume ratio and infiltrated into the leaves of *N. benthamiana*. Plants inoculated with TRV1 + pTRV2-*GFP* were included as negative controls. One week after inoculation, plants were sprayed with 0.5 mM SA for 3 days. At one day post the last spray, six samples (apical systemic leaves from three plants) were harvested for the analysis of *NbBT1*, *NbPR1a* and *NbPR2* transcription. A reduction over 70% in the transcription of *NbBT1* was used as the criteria of successful silencing.

### RNA-seq library preparation, sequencing, and data analysis

RNA isolation, library preparation, sequencing, and data analysis were conducted by Seqhealth Ltd. (China). Apical leaves from three *N. benthamiana* plants were pooled as one biological replicate and three independent biological replicates were used for each treatment. Total RNA was extracted using TRIzol Reagent (Cat # 15596026, Thermo Scientific, USA) following the manufacturer’s protocol, followed by DNase I treatment (Cat # M0303L, NEB, USA) to eliminate genomic DNA contamination. RNA purity and integrity were assessed using a Nanodrop OneC spectrophotometer (Thermo Scientific, USA) and a LabChip GX Touch system (Revvity, USA), respectively. RNA concentration was determined using a Qubit 3.0 fluorometer with the Qubit RNA Broad Range Assay kit (Cat # Q10210, Thermo Scientific, USA). RNA-seq libraries were prepared using the KC-Digital stranded mRNA-seq Library Prep Kit (Cat # DR09202, Seqhealth Ltd., China) as per the manufacturer’s instructions. The enriched libraries corresponding to fragments ranging from 200 to 500 bp were sequenced on a DNBSEQ-T7 platform (MGI Tech Co., Ltd. China) using the PE150 model.

Raw sequencing data were filtered using fastp (v0.23.2) to remove low-quality reads and adapter sequences. Unique molecular identifier (UMI) sequences were identified and extracted from clean reads using umikit (v1.0) based on anchor sequences. Paired reads possessing valid 5’ and 3’ UMI anchors were retained as valid UMI reads, whereas reads with insert lengths shorter than 15 nt were discarded. The remaining valid UMI reads were aligned to the *N. benthamiana* LAB360 reference genome (available at https://solgenomics.net/) using STAR (version 2.5.3a) with default parameters. UMI-based deduplication and error correction were performed using the customized gencore software (v1.0). UMI-deduplicated reads mapped to exon regions were counted using featureCounts (Subread-1.5.1; Bioconductor) and gene expression levels were calculated accordingly. Differentially expressed genes were identified using the edgeR package (version 3.40.2) with a fold-change cutoff of 2 and a *P*-value threshold of 0.05. Gene ontology (GO) enrichment analysis was performed using KOBAS (version: 2.1.1) with a *P*-value cutoff of 0.05.

### Statistics and reproducibility

qPCR data of virus accumulation and relative mRNA levels were normalized to plant *actin* using the 2^-ΔCt^ method. Prior to the analysis, normal distribution analysis using Shapiro-Wilk test was performed. Since all data of phytohormone content and relative PAL enzyme activity, and the majority of data of relative mRNA levels (84 out of 94 cases) were normally distributed, Levene’s test for equality of variances and two-sided Student’s independent t-test were employed. *P* values of t-test were determined according to the results of Levene’s test. Non-parametric Mann-Whitney U tests were used for the data of virus quantity as in more than one third (25 out of 69) cases they did not follow normal distribution. All analyses of phytohormone content, relative PAL enzyme activity, virus quantity, and relative mRNA levels were conducted using SPSS Statistics 21.0 and Excel. Whitefly preference data were analyzed using R package lme4 (version 4.6.0) with Generalized Linear Mixed Model (GLMM) with binomial distribution and logit link function. All data are presented as the mean ± standard error of the mean (mean ± SEM), and differences were considered statistically significant at *P* < 0.05. All experiments described in this study were repeated at least once with similar results, and data from representative experiments are shown.

## Supporting information

S1 Table Primers used in this study. (DOCX)

S1 Fig Symptoms induced by begomoviruses and begomovirus-betasatellite complexes in *N. benthamiana* plants. *N. benthamiana* plants were inoculated with pBINPLUS (control), cotton leaf curl Multan virus (CLCuMuV)-cotton leaf curl Multan betasatellite (CLCuMuB), tobacco curly shoot virus (TbCSV)-tobacco curly shoot betasatellite (TbCSB), Sri Lankan cassava mosaic virus (SLCMV) A + B, papaya leaf curl China virus (PaLCuCNV) or tomato yellow leaf curl virus (TYLCV). Pictures were taken at 10 days post inoculation.
(DOCX)

S2 Fig The contents of various hormones and related metabolates in *N. benthamiana* plants upon the infection of diverse begomoviruses and begomovirus-betasatellite complexes. *N. benthamiana* plants were inoculated with pBINPLUS (control), begomovirus-betasatellite complexes or begomoviruses. At 10 days post inoculation, the contents of jasmonic acid (JA), jasmonoyl-isoleucine (JA-Ile), 12-oxo-phytodienoic acid (OPDA), abscisic acid (ABA), indole-3-acetic acid (IAA) were analyzed. n = 6 samples (2–3 plants per sample). Comparisons were made between control and begomovirus-betasatellite complexes or begomoviruses-inoculated plants. Data were analyzed using the two-sided Student’s t-test and expressed as the mean ± SEM. ns stands for no significant difference, *P < 0.05, **P < 0.01, ***P < 0.001.
(DOCX)

S3 Fig The contents of SA in *N. benthamiana* plants that were inoculated with infiltration buffer, untransformed agrobacteria and agrobacteria containing pBINPLUS. *N. benthamiana* plants were inoculated with infiltration buffer, untransformed agrobacteria (strain EHA105) and agrobacteria (strain EHA105) containing pBINPLUS (empty vector). At 10 days post inoculation, the contents of SA were analyzed. n = 4 samples (3 plants per sample). Data were analyzed using the two-sided Student’s t-test and expressed as the mean ± SEM. ns stands for no significant difference.
(DOCX)

S4 Fig Differentially expressed genes in SLCMV A + B-infected *N. benthamiana* plants as compared with control and GO enrichment analysis of these genes. *N. benthamiana* plants were inoculated with pBINPLUS (empty vector, control) or SLCMV A + B. At 10 days post inoculation, plants were sampled for RNA-seq. Differentially expressed genes were shown in the volcano plot with upregulated genes marked as red dots and downregulated genes marked as blue dots (A). Identified genes were subjected to gene ontology (GO) enrichment analysis (B).
(DOCX)

S5 Fig The effect of AIP treatment on the contents of various hormones and related metabolites in control or SLCMV-infected *N. benthamiana* plants. *N. benthamiana* plants were inoculated with pBINPLUS (control) or SLCMV A + B. At 10 days post inoculation, plants were sprayed with approximately 0.5 mL of AIP or DMSO (solvent) solution per plant per day for three consecutive days. One day post the last spray, plants were sampled for the profiling of salicylic acid (SA, A), abscisic acid (ABA, B), jasmonic acid (JA, C), jasmonoyl-isoleucine (JA-ILE, D) and 12-oxo-phytodienoic acid (OPDA, E). n = 6 samples (3 plants per sample). Comparisons were made between DMSO and AIP-treated plants. Data were analyzed using the two-sided Student’s t-test and expressed as the mean ± SEM. ns stands for no significant difference, ***P < 0.001.
(DOCX)

S6 Fig Symptoms of SLCMV A + B infected cassava plants. Cassava plants were inoculated with pBINPLUS (control) or SLCMV A + B and pictures were taken at 45 days post inoculation. (A-B, E-F) Side and top view of control (A-B) and SLCMV A + B-infected (D-E) cassava plants; (C and F) Enlarged view of cassava leaves.
(DOCX)

S7 Fig Mutations of BC1 and/or BV1 in DNA-B impair SLCMV infection in *N. benthamiana* plants. *N. benthamiana* plants were inoculated with SLCMV DNA-A alone or with wild type or mutant DNA-B. At 10 days post inoculation, plants were subjected to the quantification of SLCMV DNA-A (A) or photographing (B). N = 9–11 plants for A. Data were analyzed using the non-parametric Mann-Whitney U test and expressed as the mean ± SEM, *P < 0.05, **P < 0.01, ***P < 0.001.
(DOCX)

S8 Fig The effect of AIP treatment on SLCMV infection in wild type and *NahG*-transgenic *N. benthamiana* plants. Wild type and *NahG*-transgenic *N. benthamiana* plants were inoculated with SLCMV A + B. At 10 days post inoculation, plants were sprayed with approximately 0.5 mL of AIP or DMSO (solvent) solution per plant per day for three consecutive days. At 10 days post the last spray, plants were sampled for the analysis of SLCMV DNA-A quantity. n = 16 plants. Comparisons were made between DMSO and AIP-treated plants. Data were analyzed using the non-parametric Mann-Whitney U test and expressed as the mean ± SEM. ns stands for no significant difference, *P < 0.05.
(DOCX)

S9 Fig Validation of SLCMV *BV1-* and SLCCNV *BV1-*transgenic *N. benthamiana* plants. (A) Picture of wild type and SLCMV *BV1*-transgenic *N. benthamiana* plants; (B) PCR amplification of SLCMV *BV1* and *NbActin* in wild type and SLCMV *BV1*-transgenic plants; (C) Picture of wild type and SLCCNV *BV1*-transgenic plants; (D) PCR amplification of SLCCNV *BV1* and *NbActin* in wild type and SLCCNV *BV1*-transgenic plants.
(DOCX)

S10 Fig SA content in wild type and SLCMV *BV1*-transgenic *N. benthamiana* plants. Wild type and SLCMV *BV1*-transgenic plants were collected and subjected to SA quantification. n = 6 samples (3 plants per sample). Data were analyzed using the two-sided Student’s t-test and expressed as the mean ± SEM, ns stands for no significant difference.
(DOCX)

S11 Fig Differentially expressed genes in SA-treated *N. benthamiana* plants as compared with ethanol solvent-treated plants and GO enrichment analysis of these genes. *N. benthamiana* plants were treated with 0.5 mM SA or ethanol solvent. Next, plants were sampled for RNA-seq. Differentially expressed genes were shown in the volcano plot with upregulated genes marked as red dots and downregulated genes marked as blue dots (A). Identified genes were subjected to gene ontology (GO) enrichment analysis (B).
(DOCX)

S12 Fig Validation of *NbBT1*-overexpression and knockout *N. benthamiana* lines. (A and C) Picture of wild type, *NbBT1*-transgenic (A) and knockout (C) plants; (B) Validation of *NbBT1* overexpression in transgenic plants. Total RNAs were extracted from wild type and *NbBT1*-transgenic plants and subjected to reverse-transcription and qPCR analysis of *NbBT1* and *NbActin*; (D) Schematic presentation of *NbBT1* knockout in two *Nbbt1* lines. CRISPR/cas9 was used and the small-guide RNA (sgRNA) sequence was shown in the diagram. Knockout was determined using Sanger sequencing. N = 6 samples (3 plants per sample) for B. Data were analyzed using the two-sided Student’s t-test and expressed as the mean ± SEM. **P < 0.01, and ***P < 0.001.
(DOCX)

S13 Fig Relative mRNA level of *NbBT1* in wild type and SLCMV *BV1*-transgenic *N. benthamiana* plants that were inoculated with TRV-*GFP* or TRV-*NbBT1.* Wild type and SLCMV *BV1*-transgenic *N. benthamiana* plants were inoculated with pTRV2-*GFP* + pTRV1, pTRV2-*NbBT1*–1 + pTRV1 or pTRV2-*NbBT1*–2 + pTRV1. At seven days post inoculation, plants were sprayed with 0.5 mM SA and then subjected to the analysis of *NbBT1* mRNA level. n = 6 samples (3 plants per sample). Data were analyzed using the two-sided Student’s t-test and expressed as the mean ± SEM. *P < 0.05, **P < 0.01.
(DOCX)
