# Supplementary material for: Subversion of the salicylic acid signaling pathway by the bipartite begomoviral protein BV1 promotes virus infection and vector preference to virus-infected plants
Source: PLoS Pathog. 2026 Jul 7;22(7):e1014354. doi: 10.1371/journal.ppat.1014354 (PMC13340803; doi:10.1371/journal.ppat.1014354)
Supplement: S1 Table — (DOCX) [file ppat.1014354.s001.docx]

**S1 Table Primers used in this study.**

| Primer | Sequence (5’-3’) | Application |
| --- | --- | --- |
| SLCMV-A-RTF | ACGCCAGGTCTGAGGCTGTA | SLCMV DNA-A quantification |
| SLCMV-A-RTF | GTTCAACAGGCCGTGGGACA |  |
| NbDNA-Actin-RTF | GCGAGTAAACCCGTAAGG | *NbActin* quantity analysis in DNA |
| NbDNA-Actin-RTR | GCTCAGGCATAGTTCACC |  |
| SLCMV-B-FLF | CGCGGATCCTATTAGACTTGGGCC | Amplification of full-length SLCMV DNA-B |
| SLCMV-B-FLR | CGCGGATCCAGATCCATGAGATATG |  |
| SLCMV-BC1-MuF | ATTGCTACTATTATTCTCCTATGGCCCCGCA | Mutagenesis of SLCMV *BC1* |
| SLCMV-BC1-MuR | TAGGAGAATAATAGTAGCAATGCAGCGTA |  |
| SLCMV-BV1-MuF | CACATGGTGGTGGCATGTAGAGAAGAGGTG | Mutagenesis of SLCMV *BV1* |
| SLCMV-BV1-MuR | TACATGCCACCACCATGTGCAGATCTCCG |  |
| NbcDNA-Actin-RTF | TCCTGATGGGCAAGTGATTAC | *NbActin* transcription analysis in cDNA |
| NbcDNA-Actin-RTR | TTGTATGTGGTCTCGTGGATTC |  |
| TRV-NbBT1-1-F | ATTCTGTGAGTAAGGTTACCGAATTCAATCATCACCTCCGGTGGCAGCCGG | Ligation of *NbBT1*-1 with pTRV2 |
| TRV-NbBT1-1-R | AGACGCGTGAGCTCGGTACCGGATCCATGGTCAATTGCTCAGCTAATCCCT |  |
| TRV-NbBT1-2-F | ATTCTGTGAGTAAGGTTACCGAATTCAGGAAGAAGAGGACAAGAAGAAACA | Ligation of *NbBT1*-2 with pTRV2 |
| TRV-NbBT1-2-R | AGACGCGTGAGCTCGGTACCGGATCCGCATTCATCAGGTTTGTCACAAATAG |  |
| NbcDNA-BT1-RTF | CAGTGAGGAGCAGATGGAGA | *NbBT1* transcription analysis in cDNA |
| NbcDNA-BT1-RTR | GATCAGGTGCATCACAGAGC |  |
| NbcDNA-PR1a-RTF | CGTTGAGATGTGGGTCGATG | *NbPR1a* transcription analysis in cDNA |
| NbcDNA-PR1a-RTR | CCTAGCACATCCAACACGAA |  |
| NbcDNA-PR2-RTF | CATTAGCAGCAGCAGGGTTG | *NbPR2* transcription analysis in cDNA |
| NbcDNA-PR2-RTR | GAGTGGAAGGTTATGTCGTGC |  |
| SLCCNV-A-RTF | GAAGCGACCAGCCGATATTA | SLCCNV DNA-A quantification |
| SLCCNV-A-RTF | GGCACATCGGGACTTCTATA |  |
| ZuDNA-Actin-RTF | TTGCTGGTCGTGATCTGACT | Zucchini *Actin* quantity analysis in DNA |
| ZuDNA-Actin-RTR | TGTCTCCAGTTCTTGCTCGT |  |
