# Supplementary material for: Subversion of the salicylic acid signaling pathway by the bipartite begomoviral protein BV1 promotes virus infection and vector preference to virus-infected plants
Source: PLoS Pathog. 2026 Jul 7;22(7):e1014354. doi: 10.1371/journal.ppat.1014354 (PMC13340803; doi:10.1371/journal.ppat.1014354)
Supplement: S1 Fig — N. benthamiana plants were inoculated with pBINPLUS (control), cotton leaf curl Multan virus (CLCuMuV)-cotton leaf curl Multan betasatellite (CLCuMuB), tobacco curly shoot virus (TbCSV)-tobacco curly shoot betasatellite (TbCSB), Sri Lankan cassava mosaic virus (SLCMV) A + B, papaya leaf curl China virus (PaLCuCNV) or tomato yellow leaf curl virus (TYLCV). Pictures were taken at 10 days post inoculation. (DOCX) [file ppat.1014354.s002.docx]

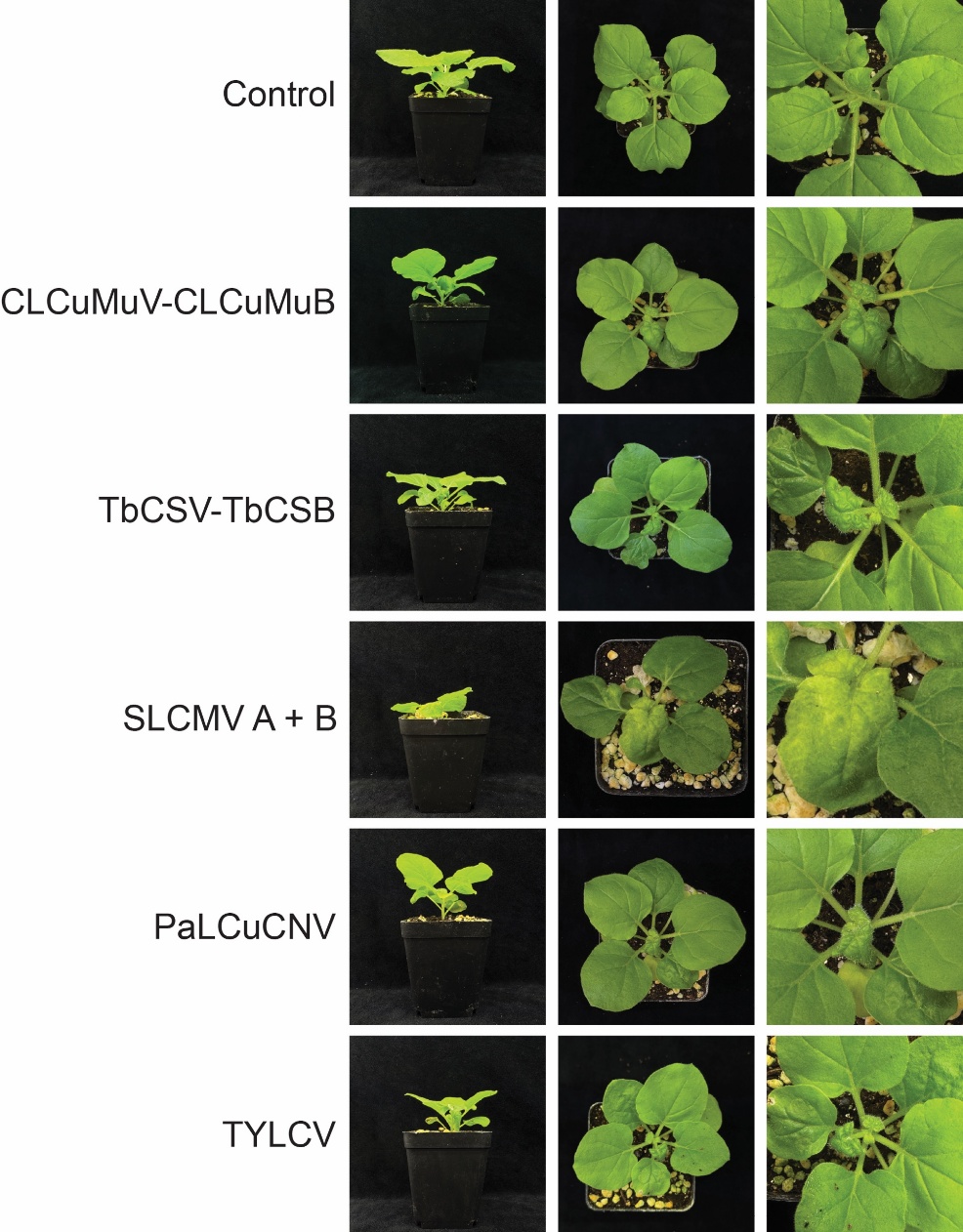


**S1 Fig Symptoms induced by begomoviruses and begomovirus-betasatellite complexes in *N. benthamiana* plants.**

*N. benthamiana* plants were inoculated with pBINPLUS (control), cotton leaf curl Multan virus (CLCuMuV)-cotton leaf curl Multan betasatellite (CLCuMuB), tobacco curly shoot virus (TbCSV)-tobacco curly shoot betasatellite (TbCSB), Sri Lankan cassava mosaic virus (SLCMV) A+B, papaya leaf curl China virus (PaLCuCNV) or tomato yellow leaf curl virus (TYLCV). Pictures were taken at 10 days post inoculation.
