# Supplementary material for: Subversion of the salicylic acid signaling pathway by the bipartite begomoviral protein BV1 promotes virus infection and vector preference to virus-infected plants
Source: PLoS Pathog. 2026 Jul 7;22(7):e1014354. doi: 10.1371/journal.ppat.1014354 (PMC13340803; doi:10.1371/journal.ppat.1014354)
Supplement: S2 Fig — N. benthamiana plants were inoculated with pBINPLUS (control), begomovirus-betasatellite complexes or begomoviruses. At 10 days post inoculation, the contents of jasmonic acid (JA), jasmonoyl-isoleucine (JA-Ile), 12-oxo-phytodienoic acid (OPDA), abscisic acid (ABA), indole-3-acetic acid (IAA) were analyzed. n = 6 samples (2–3 plants per sample). Comparisons were made between control and begomovirus-betasatellite complexes or begomoviruses-inoculated plants. Data were analyzed using the two-sided Student’s t-test and expressed as the mean ± SEM. ns stands for no significant difference, *P < 0.05, **P < 0.01, ***P < 0.001. (DOCX) [file ppat.1014354.s003.docx]

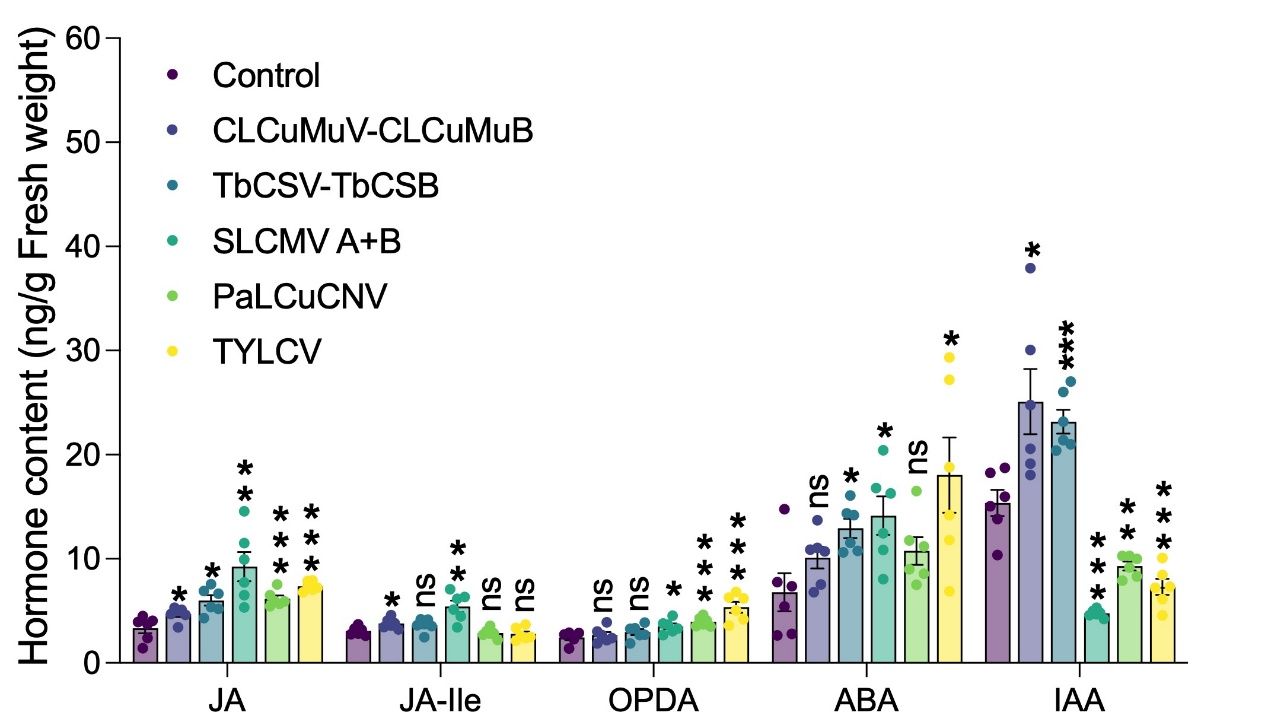


**S2 Fig. The contents of** **various hormones and related metabolites in *N. benthamiana* plants upon the infection of diverse begomoviruses and begomovirus-betasatellite complexes.**

*N. benthamiana* plants were inoculated with pBINPLUS (control), begomovirus-betasatellite complexes or begomoviruses. At 10 days post inoculation, the contents of jasmonic acid (JA), jasmonoyl-isoleucine (JA-Ile), 12-oxo-phytodienoic acid (OPDA), abscisic acid (ABA), indole-3-acetic acid (IAA) were analyzed. n=6 samples (2-3 plants per sample). Comparisons were made between control and begomovirus-betasatellite complexes or begomoviruses-inoculated plants. Data were analyzed using the two-sided Student’s t-test and expressed as the mean ± SEM. ns stands for no significant difference, **P* < 0.05, ***P* < 0.01, ****P* < 0.001.
