# Supplementary material for: Subversion of the salicylic acid signaling pathway by the bipartite begomoviral protein BV1 promotes virus infection and vector preference to virus-infected plants
Source: PLoS Pathog. 2026 Jul 7;22(7):e1014354. doi: 10.1371/journal.ppat.1014354 (PMC13340803; doi:10.1371/journal.ppat.1014354)
Supplement: S3 Fig — N. benthamiana plants were inoculated with infiltration buffer, untransformed agrobacteria (strain EHA105) and agrobacteria (strain EHA105) containing pBINPLUS (empty vector). At 10 days post inoculation, the contents of SA were analyzed. n = 4 samples (3 plants per sample). Data were analyzed using the two-sided Student’s t-test and expressed as the mean ± SEM. ns stands for no significant difference. (DOCX) [file ppat.1014354.s004.docx]

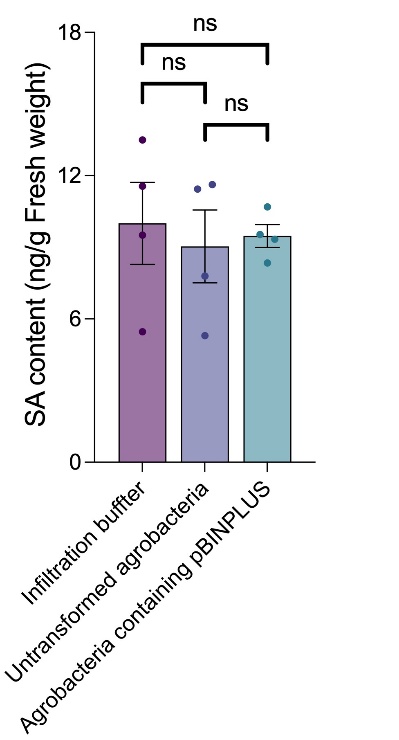


**S3 Fig. The contents of SA in *N. benthamiana* plants that were inoculated with infiltration buffer, untransformed agrobacteria and agrobacteria containing pBINPLUS.**

*N. benthamiana* plants were inoculated with infiltration buffer, untransformed agrobacteria (strain EHA105) and agrobacteria (strain EHA105) containing pBINPLUS (empty vector). At 10 days post inoculation, the contents of SA were analyzed. n=4 samples (3 plants per sample). Data were analyzed using the two-sided Student’s t-test and expressed as the mean ± SEM. ns stands for no significant difference.
