# Supplementary material for: Subversion of the salicylic acid signaling pathway by the bipartite begomoviral protein BV1 promotes virus infection and vector preference to virus-infected plants
Source: PLoS Pathog. 2026 Jul 7;22(7):e1014354. doi: 10.1371/journal.ppat.1014354 (PMC13340803; doi:10.1371/journal.ppat.1014354)
Supplement: S4 Fig — N. benthamiana plants were inoculated with pBINPLUS (empty vector, control) or SLCMV A + B. At 10 days post inoculation, plants were sampled for RNA-seq. Differentially expressed genes were shown in the volcano plot with upregulated genes marked as red dots and downregulated genes marked as blue dots (A). Identified genes were subjected to gene ontology (GO) enrichment analysis (B). (DOCX) [file ppat.1014354.s005.docx]

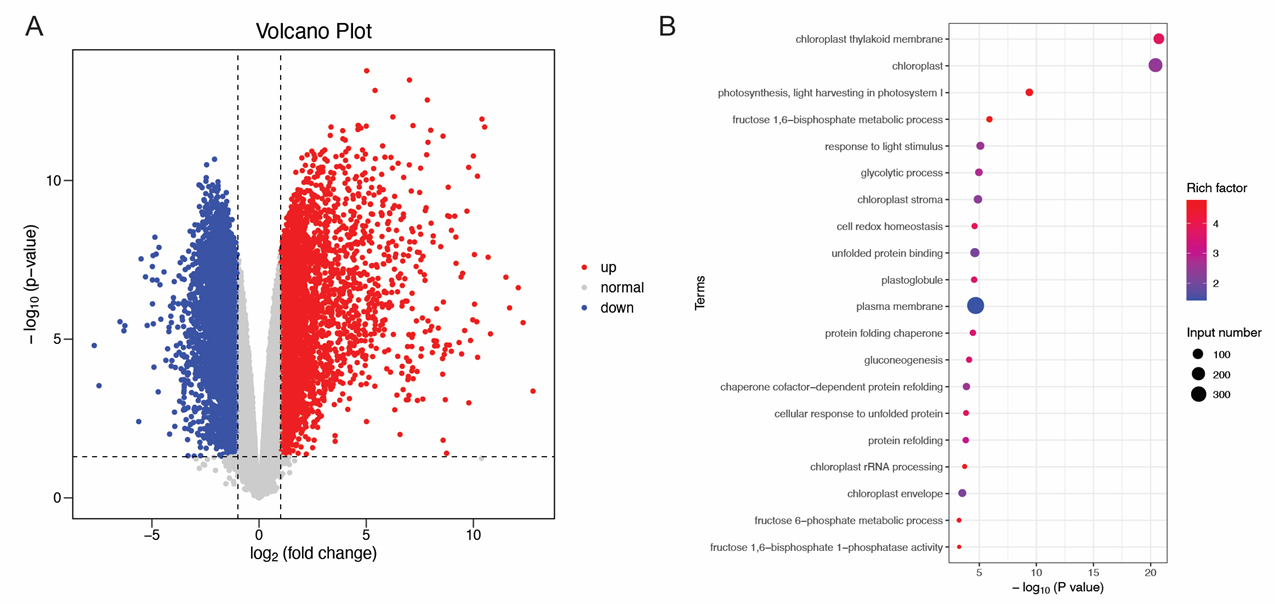


**S4 Fig. Differentially expressed genes in SLCMV A+B-infected *N. benthamiana* plants as compared with control and GO enrichment analysis of these genes.**

*N. benthamiana* plants were inoculated with pBINPLUS (empty vector, control) or SLCMV A+B. At 10 days post inoculation, plants were sampled for RNA-seq. Differentially expressed genes were shown in the volcano plot with upregulated genes marked as red dots and downregulated genes marked as blue dots (A). Identified genes were subjected to gene ontology (GO) enrichment analysis (B).
