# Supplementary material for: Subversion of the salicylic acid signaling pathway by the bipartite begomoviral protein BV1 promotes virus infection and vector preference to virus-infected plants
Source: PLoS Pathog. 2026 Jul 7;22(7):e1014354. doi: 10.1371/journal.ppat.1014354 (PMC13340803; doi:10.1371/journal.ppat.1014354)
Supplement: S5 Fig — N. benthamiana plants were inoculated with pBINPLUS (control) or SLCMV A + B. At 10 days post inoculation, plants were sprayed with approximately 0.5 mL of AIP or DMSO (solvent) solution per plant per day for three consecutive days. One day post the last spray, plants were sampled for the profiling of salicylic acid (SA, A), abscisic acid (ABA, B), jasmonic acid (JA, C), jasmonoyl-isoleucine (JA-ILE, D) and 12-oxo-phytodienoic acid (OPDA, E). n = 6 samples (3 plants per sample). Comparisons were made between DMSO and AIP-treated plants. Data were analyzed using the two-sided Student’s t-test and expressed as the mean ± SEM. ns stands for no significant difference, ***P < 0.001. (DOCX) [file ppat.1014354.s006.docx]

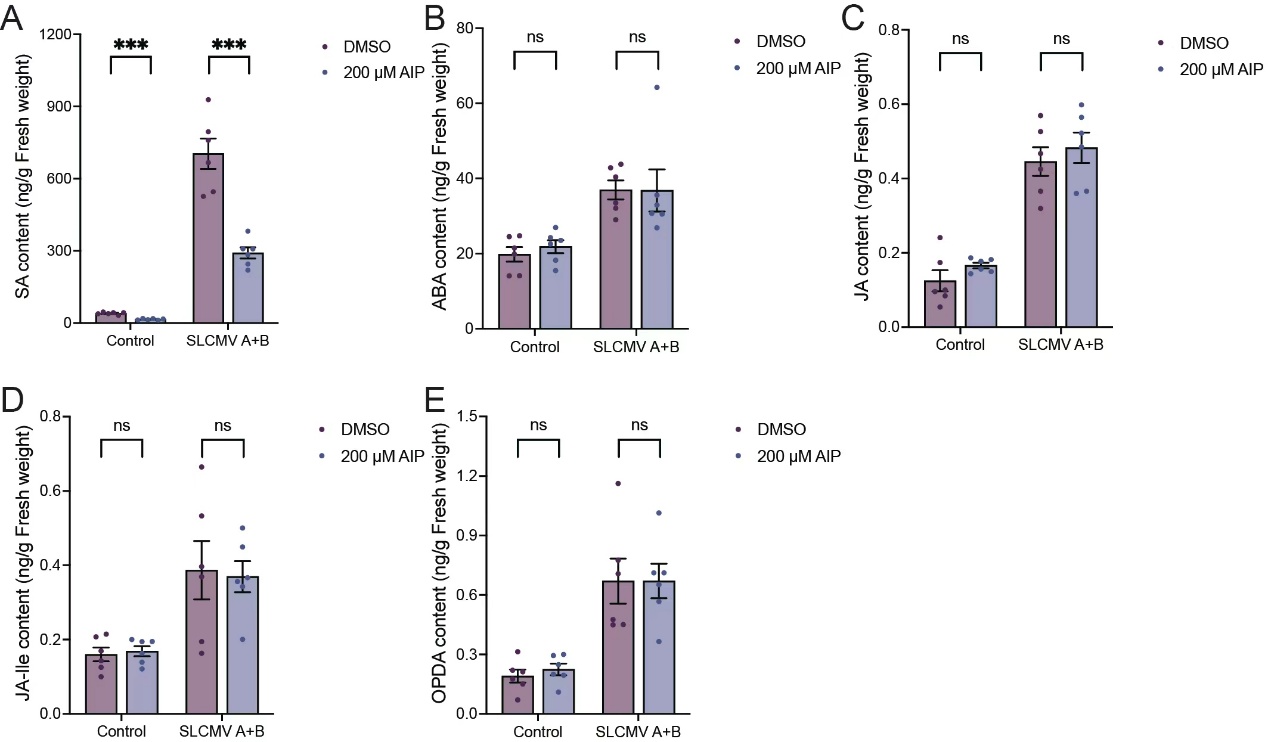


**S5 Fig. The effect of AIP treatment on the contents of various hormones and related metabolites in control or SLCMV-infected *N. benthamiana* plants.**

*N. benthamiana* plants were inoculated with pBINPLUS (control) or SLCMV A+B. At 10 days post inoculation, plants were sprayed with approximately 0.5 mL of AIP or DMSO (solvent) solution per plant per day for three consecutive days. One day post the last spray, plants were sampled for the profiling of salicylic acid (SA, A), abscisic acid (ABA, B), jasmonic acid (JA, C), jasmonoyl-isoleucine (JA-Ile, D) and 12-oxo-phytodienoic acid (OPDA, E). n=6 samples (3 plants per sample). Comparisons were made between DMSO and AIP-treated plants. Data were analyzed using the two-sided Student’s t-test and expressed as the mean ± SEM. ns stands for no significant difference, ****P* < 0.001.
