# Supplementary material for: Subversion of the salicylic acid signaling pathway by the bipartite begomoviral protein BV1 promotes virus infection and vector preference to virus-infected plants
Source: PLoS Pathog. 2026 Jul 7;22(7):e1014354. doi: 10.1371/journal.ppat.1014354 (PMC13340803; doi:10.1371/journal.ppat.1014354)
Supplement: S6 Fig — Cassava plants were inoculated with pBINPLUS (control) or SLCMV A + B and pictures were taken at 45 days post inoculation. (A-B, E-F) Side and top view of control (A-B) and SLCMV A + B-infected (D-E) cassava plants; (C and F) Enlarged view of cassava leaves. (DOCX) [file ppat.1014354.s007.docx]

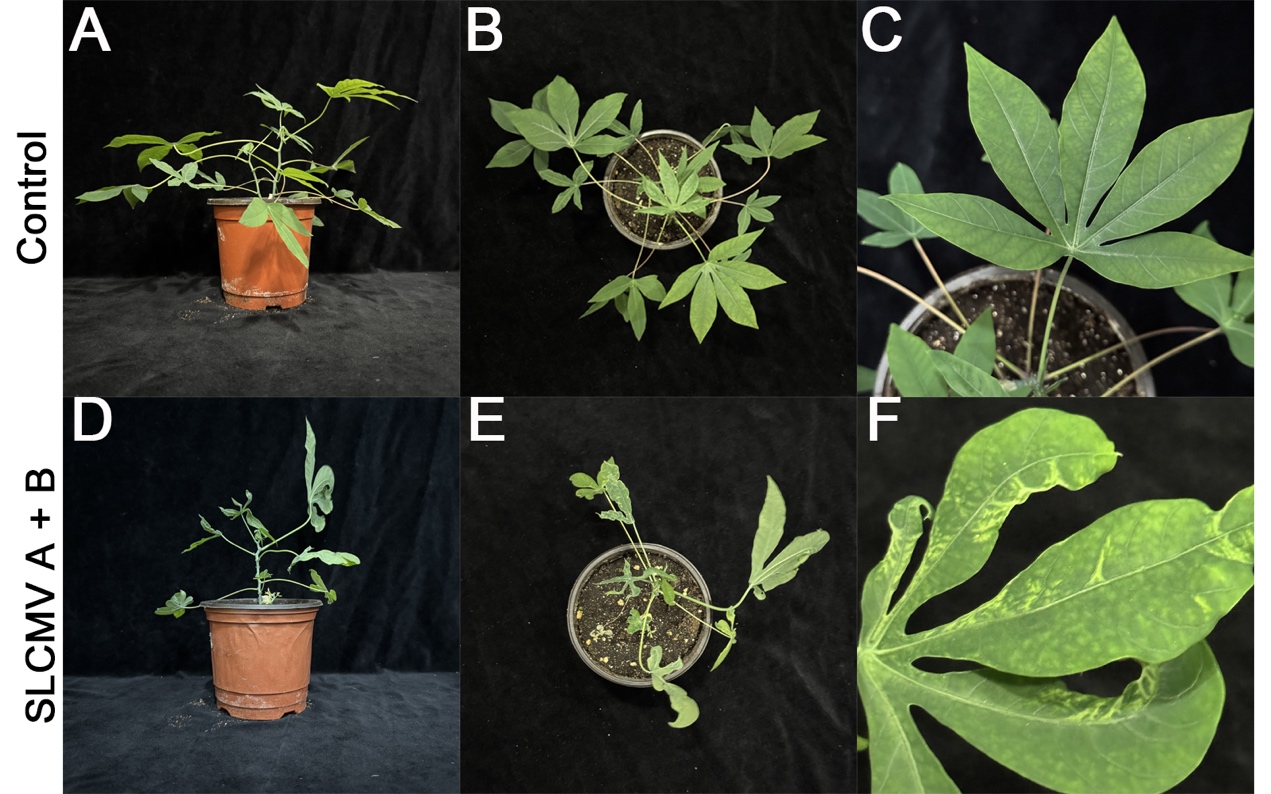


**S6 Fig. Symptoms of SLCMV A+B infected cassava plants.**

Cassava plants were inoculated with pBINPLUS (control) or SLCMV A+B and pictures were taken at 45 days post inoculation. (A-B, E-F) Side and top view of control (A-B) and SLCMV A+B-infected (D-E) cassava plants; (C and F) Enlarged view of cassava leaves.
