# Supplementary material for: Subversion of the salicylic acid signaling pathway by the bipartite begomoviral protein BV1 promotes virus infection and vector preference to virus-infected plants
Source: PLoS Pathog. 2026 Jul 7;22(7):e1014354. doi: 10.1371/journal.ppat.1014354 (PMC13340803; doi:10.1371/journal.ppat.1014354)
Supplement: S7 Fig — N. benthamiana plants were inoculated with SLCMV DNA-A alone or with wild type or mutant DNA-B. At 10 days post inoculation, plants were subjected to the quantification of SLCMV DNA-A (A) or photographing (B). N = 9–11 plants for A. Data were analyzed using the non-parametric Mann-Whitney U test and expressed as the mean ± SEM, *P < 0.05, **P < 0.01, ***P < 0.001. (DOCX) [file ppat.1014354.s008.docx]

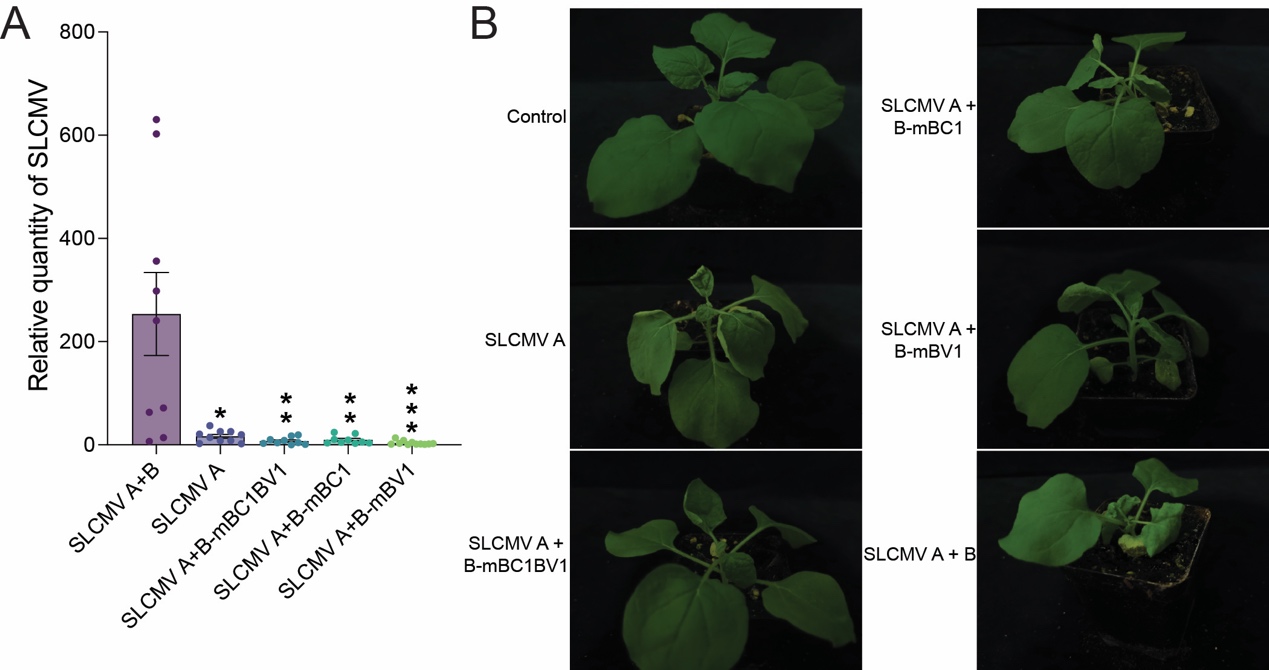


**S7 Fig. Mutations of BC1 and/or BV1 in DNA-B impair SLCMV infection in *N. benthamiana* plants.**

*N. benthamiana* plants were inoculated with SLCMV DNA-A alone or with wild type or mutant DNA-B. At 10 days post inoculation, plants were subjected to the quantification of SLCMV DNA-A (A) or photographing (B). n=9-11 plants for A. Data were analyzed using the non-parametric Mann-Whitney U test and expressed as the mean ± SEM, **P* < 0.05, ***P* < 0.01, ****P* < 0.001.
