# Supplementary material for: Subversion of the salicylic acid signaling pathway by the bipartite begomoviral protein BV1 promotes virus infection and vector preference to virus-infected plants
Source: PLoS Pathog. 2026 Jul 7;22(7):e1014354. doi: 10.1371/journal.ppat.1014354 (PMC13340803; doi:10.1371/journal.ppat.1014354)
Supplement: S8 Fig — Wild type and NahG-transgenic N. benthamiana plants were inoculated with SLCMV A + B. At 10 days post inoculation, plants were sprayed with approximately 0.5 mL of AIP or DMSO (solvent) solution per plant per day for three consecutive days. At 10 days post the last spray, plants were sampled for the analysis of SLCMV DNA-A quantity. n = 16 plants. Comparisons were made between DMSO and AIP-treated plants. Data were analyzed using the non-parametric Mann-Whitney U test and expressed as the mean ± SEM. ns stands for no significant difference, *P < 0.05. (DOCX) [file ppat.1014354.s009.docx]

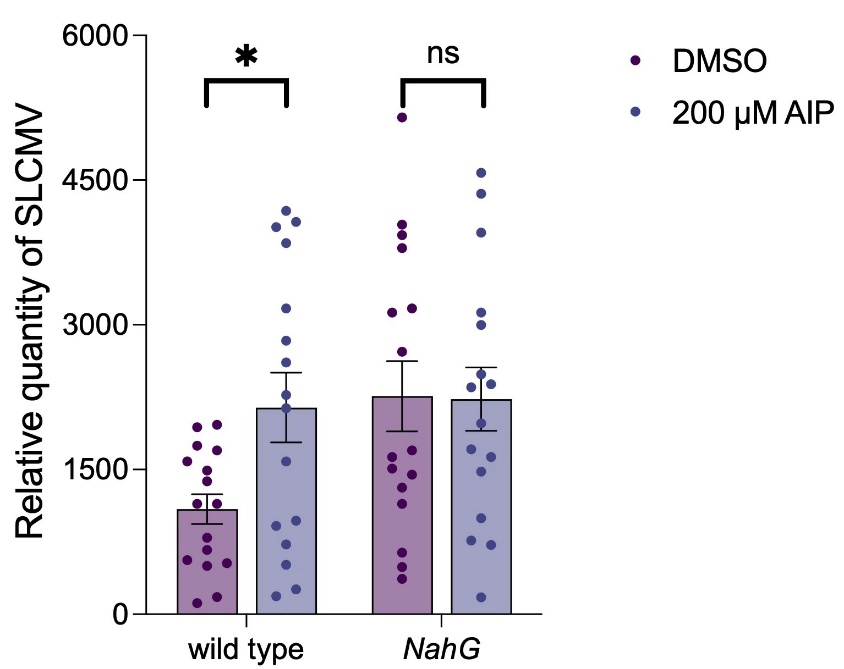


**S8 Fig. The effect of AIP treatment on SLCMV infection in wild type and *NahG*-transgenic *N. benthamiana* plants.**

Wild type and *NahG*-transgenic *N. benthamiana* plants were inoculated with SLCMV A+B. At 10 days post inoculation, plants were sprayed with approximately 0.5 mL of AIP or DMSO (solvent) solution per plant per day for three consecutive days. At 10 days post the last spray, plants were sampled for the analysis of SLCMV DNA-A quantity. n= 16 plants. Comparisons were made between DMSO and AIP-treated plants. Data were analyzed using the non-parametric Mann-Whitney U test and expressed as the mean ± SEM. ns stands for no significant difference, **P* < 0.05.
