# Supplementary material for: Subversion of the salicylic acid signaling pathway by the bipartite begomoviral protein BV1 promotes virus infection and vector preference to virus-infected plants
Source: PLoS Pathog. 2026 Jul 7;22(7):e1014354. doi: 10.1371/journal.ppat.1014354 (PMC13340803; doi:10.1371/journal.ppat.1014354)
Supplement: S9 Fig — (A) Picture of wild type and SLCMV BV1-transgenic N. benthamiana plants; (B) PCR amplification of SLCMV BV1 and NbActin in wild type and SLCMV BV1-transgenic plants; (C) Picture of wild type and SLCCNV BV1-transgenic plants; (D) PCR amplification of SLCCNV BV1 and NbActin in wild type and SLCCNV BV1-transgenic plants. (DOCX) [file ppat.1014354.s010.docx]

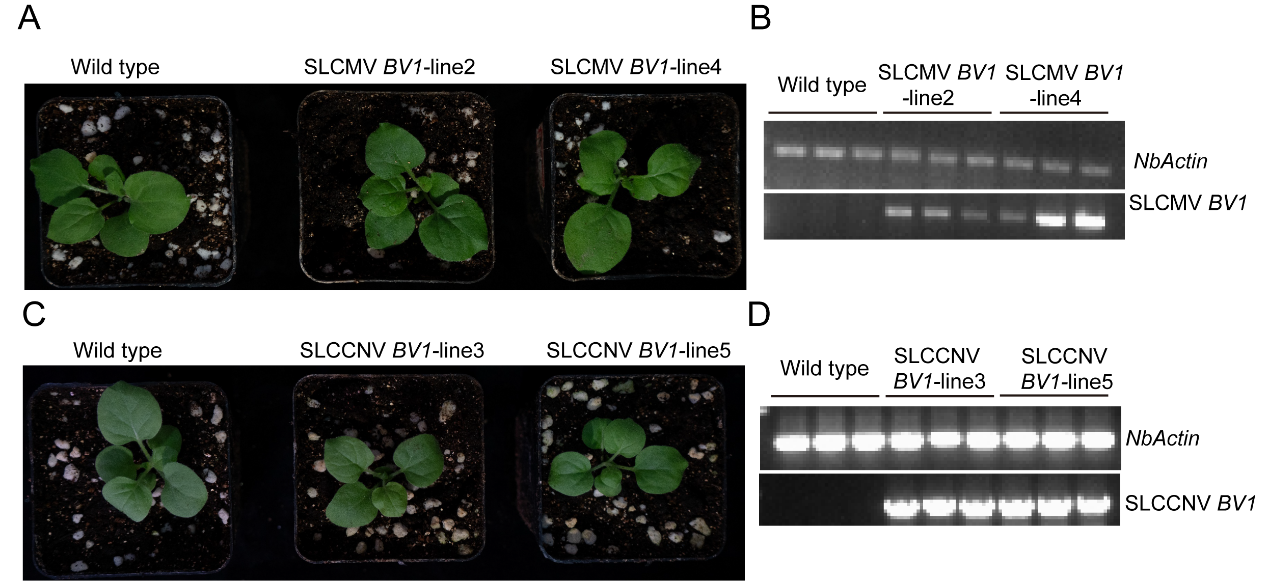


**S9 Fig. Validation of SLCMV *BV1-* and SLCCNV *BV1-*transgenic *N. benthamiana* plants.**

(A) Picture of wild type and SLCMV *BV1*-transgenic *N. benthamiana* plants; (B) PCR amplification of SLCMV *BV1* and *NbActin* in wild type and SLCMV *BV1*-transgenic plants; (C) Picture of wild type and SLCCNV *BV1*-transgenic plants; (D) PCR amplification of SLCCNV *BV1* and *NbActin* in wild type and SLCCNV *BV1*-transgenic plants.
