# Supplementary material for: Subversion of the salicylic acid signaling pathway by the bipartite begomoviral protein BV1 promotes virus infection and vector preference to virus-infected plants
Source: PLoS Pathog. 2026 Jul 7;22(7):e1014354. doi: 10.1371/journal.ppat.1014354 (PMC13340803; doi:10.1371/journal.ppat.1014354)
Supplement: S10 Fig — Wild type and SLCMV BV1-transgenic plants were collected and subjected to SA quantification. n = 6 samples (3 plants per sample). Data were analyzed using the two-sided Student’s t-test and expressed as the mean ± SEM, ns stands for no significant difference. (DOCX) [file ppat.1014354.s011.docx]

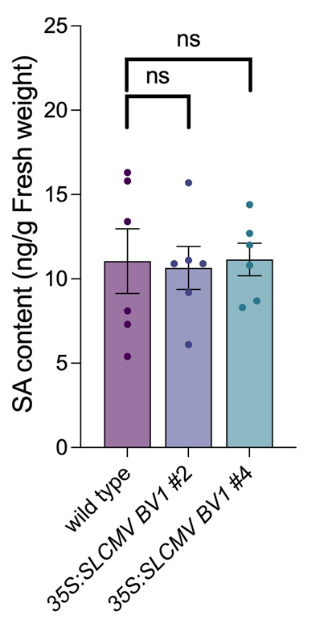


**S10 Fig. SA content in wild type and SLCMV *BV1*-transgenic *N. benthamiana* plants.**

Wild type and SLCMV *BV1*-transgenic plants were collected and subjected to SA quantification. n = 6 samples (3 plants per sample). Data were analyzed using the two-sided Student’s t-test and expressed as the mean ± SEM, ns stands for no significant difference.
