# Supplementary material for: Subversion of the salicylic acid signaling pathway by the bipartite begomoviral protein BV1 promotes virus infection and vector preference to virus-infected plants
Source: PLoS Pathog. 2026 Jul 7;22(7):e1014354. doi: 10.1371/journal.ppat.1014354 (PMC13340803; doi:10.1371/journal.ppat.1014354)
Supplement: S12 Fig — (A and C) Picture of wild type, NbBT1-transgenic (A) and knockout (C) plants; (B) Validation of NbBT1 overexpression in transgenic plants. Total RNAs were extracted from wild type and NbBT1-transgenic plants and subjected to reverse-transcription and qPCR analysis of NbBT1 and NbActin; (D) Schematic presentation of NbBT1 knockout in two Nbbt1 lines. CRISPR/cas9 was used and the small-guide RNA (sgRNA) sequence was shown in the diagram. Knockout was determined using Sanger sequencing. N = 6 samples (3 plants per sample) for B. Data were analyzed using the two-sided Student’s t-test and expressed as the mean ± SEM. **P < 0.01, and ***P < 0.001. (DOCX) [file ppat.1014354.s013.docx]

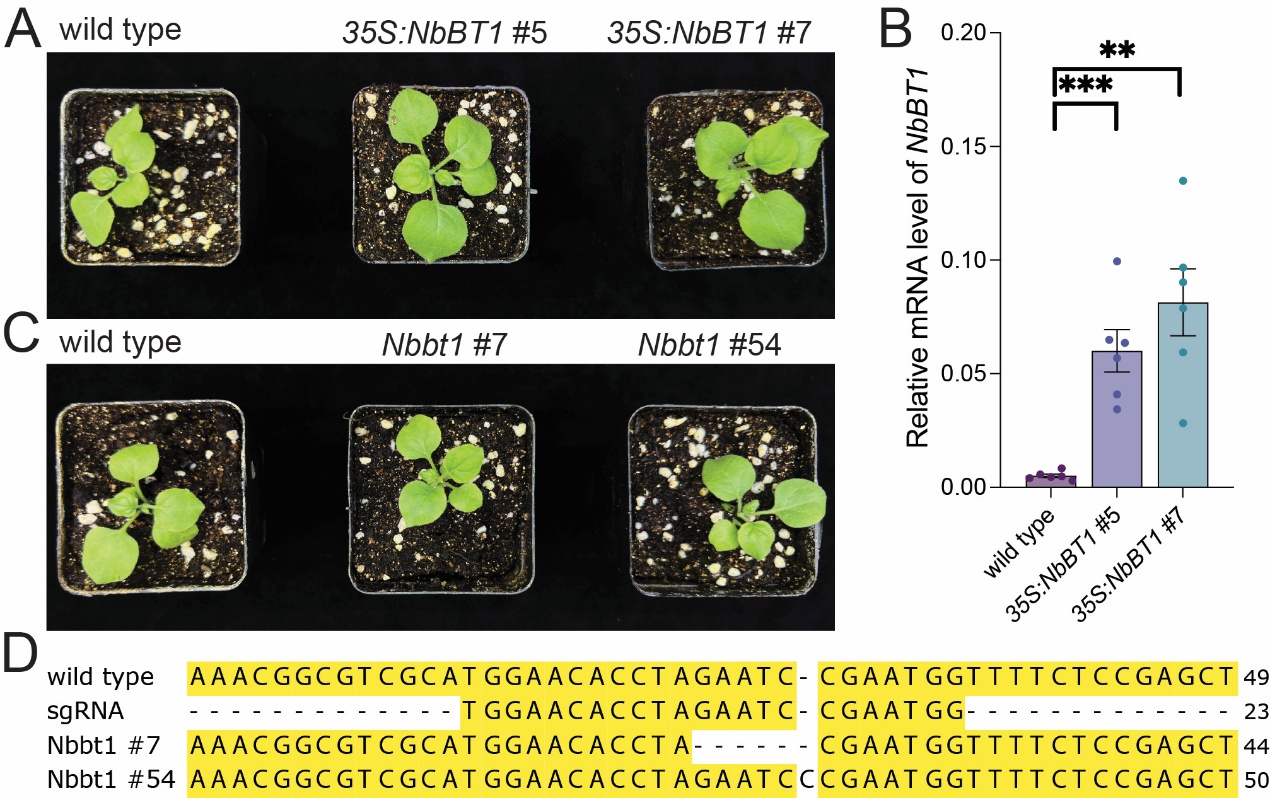


**S12 Fig. Validation of *NbBT1*-overexpression and knockout *N. benthamiana* lines.**

(A and C) Picture of wild type, *NbBT1*-transgenic (A) and knockout (C) plants; (B) Validation of *NbBT1* overexpression in transgenic plants. Total RNAs were extracted from wild type and *NbBT1*-transgenic plants and subjected to reverse-transcription and qPCR analysis of *NbBT1* and *NbActin*; (D) Schematic presentation of *NbBT1* knockout in two *Nbbt1* lines. CRISPR/cas9 was used and the small-guide RNA (sgRNA) sequence was shown in the diagram. Knockout was determined using Sanger sequencing. N = 6 samples (3 plants per sample) for B. Data were analyzed using the two-sided Student’s t-test and expressed as the mean ± SEM. ***P* < 0.01, and ****P* < 0.001.
