# Supplementary material for: Subversion of the salicylic acid signaling pathway by the bipartite begomoviral protein BV1 promotes virus infection and vector preference to virus-infected plants
Source: PLoS Pathog. 2026 Jul 7;22(7):e1014354. doi: 10.1371/journal.ppat.1014354 (PMC13340803; doi:10.1371/journal.ppat.1014354)
Supplement: S13 Fig — Wild type and SLCMV BV1-transgenic N. benthamiana plants were inoculated with pTRV2-GFP + pTRV1, pTRV2-NbBT1–1 + pTRV1 or pTRV2-NbBT1–2 + pTRV1. At seven days post inoculation, plants were sprayed with 0.5 mM SA and then subjected to the analysis of NbBT1 mRNA level. n = 6 samples (3 plants per sample). Data were analyzed using the two-sided Student’s t-test and expressed as the mean ± SEM. *P < 0.05, **P < 0.01. (DOCX) [file ppat.1014354.s014.docx]

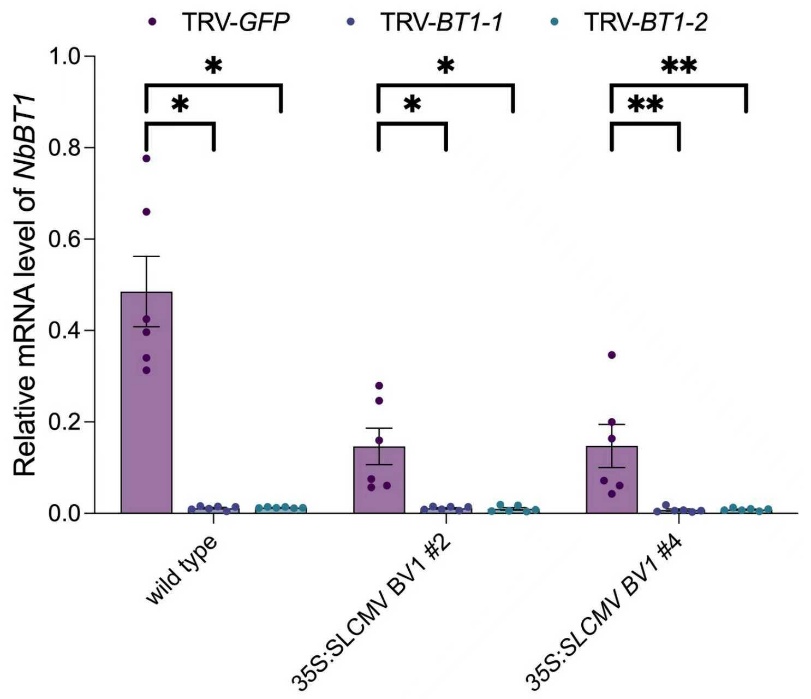


**S13 Fig. Relative mRNA level of *NbBT1* in wild type and SLCMV *BV1*-transgenic *N. benthamiana* plants that were inoculated with TRV-*GFP* or TRV-*NbBT1*.**

Wild type and SLCMV *BV1*-transgenic *N. benthamiana* plants were inoculated with pTRV2-*GFP*+pTRV1, pTRV2-*NbBT1*-1+pTRV1 or pTRV2-*NbBT1*-2+pTRV1. At seven days post inoculation, plants were sprayed with 0.5 mM SA and then subjected to the analysis of *NbBT1* mRNA level. n = 6 samples (3 plants per sample). Data were analyzed using the two-sided Student’s t-test and expressed as the mean ± SEM. **P* < 0.05, ***P* < 0.01.
