# Supplementary material for: Subversion of the salicylic acid signaling pathway by the bipartite begomoviral protein BV1 promotes virus infection and vector preference to virus-infected plants
Source: PLoS Pathog. 2026 Jul 7;22(7):e1014354. doi: 10.1371/journal.ppat.1014354 (PMC13340803; doi:10.1371/journal.ppat.1014354)
Supplement: Attachment — Submitted filename: 3 Point-to-point responses to comments PLL 2026.06.01.docx [file ppat.1014354.s015.docx]

**Point-to-point responses to reviewer comments**

In response to the comments from the three Reviewers, we have edited the text (particularly the Introduction and Discussion sections) and legends to accommodate their suggestions. In addition, eight experiments were performed to bolster our conclusion including: (1) analyzed the effect of infiltration buffer, untransformed agrobacteria and agrobacteria containing pBINPLUS on plant SA contents; (2) tested the effect of AIP treatment on the contents of SA and other hormones and related metabolites in pBINPLUS and SLCMV-inoculated plants; (3) analyzed the effect of AIP treatment on SLCMV infection in wild type and *NahG*-transgenic *N. benthamiana* plants; (4-7) re-compared whitefly preference between: (4) ethanol solvent and SA-treated *N. benthamiana* plants that were infected by pBINPLUS (control), SLCMV A+B, SLCMV A, SLCMV A+B-mBC1 or SLCMV A+B-mBV1; (5) ethanol solvent and SA-treated wild type or SLCMV *BV1*-transgenic *N. benthamiana* plants; (6) wild type and *NbBT1*-knockout *N. benthamiana* plants, and (7) ethanol solvent or SA-treated wild type and SLCCNV *BV1*-transgenic *N. benthamiana* plants; (8) compared whitefly preference between wild type and *NbBT1*-overexpressing *N. benthamiana* plants. We also reanalyzed our data of virus quantity using non-parametric Mann-Whitney U tests, and whitefly preference using binomial tests.

We thank the three Reviewers for their insightful comments that have significantly improved our manuscript. To facilitate reading, the comments from the Reviewers are copied in blue, followed by our response. The line numbers in the responses refer to those in the revised version of the manuscript with track changes.

Journal Requirements:

1) Please ensure that the CRediT author contributions listed for every co-author are completed accurately and in full.

At this stage, the following Authors/Authors require contributions: Guan-Ping Chen, Di Li, Xing Zhang, Ming-Yang Yu, Jing-Ru Zhang, Wilmer J. Cuellar, Xiao-Wei Wang, Shu-Sheng Liu, Yin-Quan Liu, and Li-Long pan. Please ensure that the full contributions of each author are acknowledged in the "Add/Edit/Remove Authors" section of our submission form.

The list of CRediT author contributions may be found here: https://journals.plos.org/plospathogens/s/authorship#loc-author-contributions

**Author’s Response (AR):** Thank you for pointing this out. We have added contributions to all authors in the online submission form.

2) Please upload all main figures as separate Figure files in .tif or .eps format. For more information about how to convert and format your figure files please see our guidelines:

https://journals.plos.org/plospathogens/s/figures

**AR:** Thank you for pointing this out. We have uploaded all main figures as separate files in tif format.

3) We have noticed that you have uploaded Supporting Information files, but you have not included a list of legends. Please add a full list of legends for your Supporting Information files after the references list.

**AR:** Thank you for pointing this out. We have added a full list of legends for Supporting Information files after the references list.

4) Please amend your detailed Financial Disclosure statement. This is published with the article. It must therefore be completed in full sentences and contain the exact wording you wish to be published.

- State the initials, alongside each funding source, of each author to receive each grant. For example: "This work was supported by the National Institutes of Health (####### to AM; ###### to CJ) and the National Science Foundation (###### to AM)."

- State what role the funders took in the study. If the funders had no role in your study, please state: "The funders had no role in study design, data collection and analysis, decision to publish, or preparation of the manuscript.".

If you did not receive any funding for this study, please simply state: u201cThe authors received no specific funding for this work.u201d

**AR:** Thank you for the pointing this out. We have revised our Financial Disclosure statement accordingly. See lines 779-783.

5) Figures: S1, S4, S5, S6, and S9: Please confirm (a) that you are the photographer; or (b) provide written permission from the photographer to publish the photo(s) under our CC BY 4.0 license.

**AR:** We confirm that the first author (Guan-Ping Chen) is the photographer of Figures S1, S4 (currently S6), S5 (currently S7), S6 (current S9), and S9 (currently S12).

Reviewers' Comments:

Reviewer's Responses to Questions

Part I – Summary Please use this section to discuss strengths/weaknesses of study, novelty/significance, general execution and scholarship.

Reviewer #1: The authors demostrate that SLCMV induces SA accumulation via the PAL pathway, and that the DNA‑B–encoded BV1 (NSP) protein suppresses SA‑induced antiviral defenses and abolishes the SA‑driven repellence to whiteflies. Using RNA‑seq and reverse genetics in N. benthamiana, they identify NbBT1 (BTB/POZ and TAZ domain‑containing protein 1) as an SA‑inducible gene and a positive regulator of SA signaling. Furthermore, the study shows that the BV1 downregulates NbBT1 transcription and, consequently, reduces the SA-responsive PR1a/PR2 expression as well as antiviral resistance and repellence to the vector. The novelty of the study is limited, as the viral suppression of SA signaling to attenuate antiviral defense and modify B. tabaci behavior has already been demonstrated by the same research group in other begomovirus systems. This includes the βC1–HSP90–NPR3‑mediated suppression of SA‑dependent immunity (Zhang et al., 2024) and the C2‑induced inhibition of SA/JA signaling that abolishes JA‑based repellence (Wang et al., 2026, First published: 16 January 2026 in Pest Manag Sci. 2026. doi: 10.1002/ps.70558.). In addition, SLCMV AC1 (Plant Stress 19, 2026, doi.org/10.1016/j.stress.2025.101134) was shown to activate anti‑whitefly defense via MYC2 accumulation/dimerization and TPS up‑regulation (a “negative relationship” with the vector), i.e., the opposite polarity of BV1.

**AR:** We thank the reviewer for the insightful comment and the references provided. As for the interaction between plant SA signaling pathway and begomoviruses, while previous studies have reported viral subversion of SA signaling pathway, these studies were conducted with monopartite begomoviruses (Medina-Puche et al. 2020; Li et al. 2025; Wang SD et al. 2026), or betasatellites associated with monopartite begomoviruses (Zhang et al. 2024; Chen et al. 2026; Zhao et al. 2026). Specifically, C2 and C4 encoded by tomato yellow leaf curl virus dampen SA signaling and biosynthesis, respectively (Medina-Puche et al. 2020; Li et al. 2025; Wang et al. 2026). In addition, βC1 proteins encoded by betasatellites associated with monopartite begomoviruses interfere with SA signaling (Zhang et al. 2024; Chen et al. 2026; Zhao et al. 2026). The genomic organization of bipartite begomoviruses differ significantly from their monopartite counterparts, and more importantly DNA-B of bipartite begomoviruses share no homology with the genome of monopartite begomoviruses or betasatellites except in the intergenic region (Elvira Fiallo-Olivé et al. 2021). How bipartite begomoviruses interact with host SA signaling pathway remain largely unknown.

As for whitefly behavior, so far only three plant factors have been implicated in the modulation of whitefly preference by begomoviruses. For example, begomoviruses and begomoviral proteins modify whitefly preference by disrupting JA signaling (Li et al. 2014; Wang SD et al. 2026) or inducing β-myrcene production and release (Liang et al. 2025). Recently, AC1 proteins encoded in two SLCMV isolates (Col and HN7) induce plant repellence against whiteflies by promoting the accumulation and dimerization of MYC2 (Wang D et al. 2026). Therefore, our understanding of the modulation of whitefly preference by begomoviruses is far from complete and more investigations are warranted.

With these rationales, we believe that this study provides distinct and significant mechanistic insights into plant interactions with begomoviruses and their whitefly vector, and in turn advance the field of plant-virus-vector interactions. We have integrated these claims into the Discussion section to highlight the novelty of our study (lines 424-435 for the interaction between plant SA signaling pathway and begomoviruses and lines 480-486 for whitefly behavior)

References

Chen GP, Wu YM, Zhao SX, Wang SD, Liu YQ, Wang XW, Liu SS, Pan LL. 2026. Viral action on the auxin signaling repressor IAA16 reveals a conserved negative regulator of plant growth and immunity. Cell Reports 45(3):117034.

Fiallo-Olivé E, Lett JM, Martin DP, Roumagnac P, Varsani A, Zerbini FM, Navas-Castillo J, ICTV Report Consortium. 2021. ICTV virus taxonomy profile: *Geminiviridae* 2021. Journal of General Virology 102(12): 001696.

Li R, Weldegergis BT, Li J, Jung C, Qu J, Sun Y, Qian H, Tee C, van Loon J, Dicke M. 2014. Virulence factors of geminivirus interact with MYC2 to subvert plant resistance and promote vector performance. Plant Cell 26: 4991-5008.

Liang P, Zeng Y, Ning J, Wu X, Wang W, Ren J, Wu QJ, Yang X, Wang SL, Guo ZJ, Su Q, Zhou XG, Turlings TCJ, Xie W, Zhang YJ. 2025. A plant virus manipulates both its host plant and the insect that facilitates its transmission. Science Advances 11(9): eadr4563.

Wang D, Zhang X, Liu Q, Zhang P, Ye J. 2026. Begomovirus AC1 protein switches sides to boost plant defense against whiteflies. Plant Stress 19: 101134.

Wang SD, Zhao SX, Wang XW, Liu SS, Pan LL, Liu YQ. 2026. Geminiviral C2 proteins promote virus transmission by subverting plant defenses upon infestation of non-vector herbivores. Pest Management Science 82(5): 4419-4428.

Zhang JR, Liu YM, Li D, Wu YJ, Zhao SX, Wang XW, Liu SS, Walling LL, Pan LL. 2024. Viral proteins resolve the virus-vector conundrum during hemipteran-mediated transmission by subverting salicylic acid signaling pathway. Nature Communications 15(1): 9448.

Zhao SX, Liu YM, Wang SD, Wang XW, Liu SS, Liu YQ, Pan LL. 2026. The βC1 protein encoded in betasatellites promotes begomovirus-whitefly coexistence by subverting vector infestation-induced plant antiviral defenses. PLoS Pathogens 22(1): e1013907.

Reviewer #2: The manuscript presents a comprehensive and mechanistically detailed study demonstrating how the bipartite begomoviral protein BV1 subverts SA signaling by downregulating NbBT1 to promote both viral infection and vector-mediated transmission. The work is compact with necessary validation demonstrated when and where required, and the use of multiple pathosystems, transgenic lines, and behavioral assays strengthens the conclusions. However, several gaps remain including missing controls (DNA-B alone, ethanol-treated BV1-transgenic RNA-seq), overclaimed causality (PAL pathway, DNA-B dependence), and incomplete datasets (NbBT1-overexpression repellence assays, silencing efficiency data) that require clarification or additional experimentation. With significant revisions addressing these points as suggested, the manuscript will be suitable for acceptance.

**AR:** Thank you for the positive assessment of our study and the insightful comments. We have performed additional experiments and revised the manuscript as per your comments. Detailed responses to your comments are provided below.

Reviewer #3: None

Part II – Major Issues: Key Experiments Required for Acceptance Please use this section to detail the key new experiments or modifications of existing experiments that should be absolutely required to validate study conclusions. Generally, there should be no more than 3 such required experiments or major modifications for a "Major Revision" recommendation. If more than 3 experiments are necessary to validate the study conclusions, then you are encouraged to recommend "Reject".

Reviewer #1: I have major concerns: The title and narrative suggest a direct impact on whitefly transmission; however, the data presented assess only insect preference/repellence (30‑min choice assays) and in‑plant viral accumulation. To substantiate such claims, the authors should include true transmission assays (acquisition/inoculation efficiencies) that causally link the BV1–BT1–SA axis to epidemiologically relevant outcomes.

**AR:** Thanks for the insightful comment. Our current results focus on the preference of whitefly vector to virus-infected plants only. Although whitefly preference is tightly associated with transmission processes, we agree that more trails such as the test of acquisition/inoculation efficiencies are need to directly link our results to whitefly-mediated transmission of begomoviruses. We have thus revised the title and narratives in the manuscript. See lines 2, 27-28, 42-43, 51-52, 122.

The whitefly choice assays are based on only n = 5 cages and, critically, treat individual insects within a cage as independent observations despite the aggregative behavior of Bemisia. This constitutes pseudoreplication, as the experimental unit is the cage rather than each insect, resulting in inflated significance and reduced statistical power. I therefore recommend conducting single‑insect arena assays, or if group assays are maintained, analyzing data at the cage level with an adequate number of replicate cages and appropriate GLMM/beta‑binomial statistical models. Moreover, N. benthamiana is a suboptimal host for whitefly bioassays as its glandular trichomes secrete acylsugars that repel or physically trap adults, potentially confounding preference or settling measurements. Finally, given that salicylic acid modulates plant volatile emissions (e.g., methyl salicylate, D‑limonene) influencing B. tabaci host choice (Int. J. Mol. Sci. 2016, 17(7), 1048), it would be advisable to characterize the volatile organic compound (VOC) profile by GC–MS under ±SA and ±BV1/±BT1 conditions to complete the causal chain (plant → VOCs → vector).

**AR:** We appreciate your detailed evaluation of our behavioral assays and the insightful comments regarding our experimental design and the underlying mechanisms. Regarding the concern of pseudoreplication, we would like to clarify that in our statistical analysis, the cage (experimental unit) was indeed treated as the replicate rather than the individual insect. As described in our Methods section, each biological replicate consisted of the combined data from two separate trials where plant positions were switched to eliminate positional bias. Fifty whiteflies per cage (25 whiteflies per trail) were used to ensure a robust behavioral response.

We agree that more replicates (cages) will contribute to a better dissection of whitefly preference, and thus have repeated all *N. benthamiana* plant-related whitefly preference experiments with ten replicates, including 1) ethanol solvent vs SA-treated *N. benthamiana* plants that were infected by pBINPLUS (control), SLCMV A+B, SLCMV A, SLCMV A+B-mBC1 or SLCMV A+B-mBV1; 2) ethanol solvent vs SA-treated wild type or SLCMV *BV1*-transgenic *N. benthamiana* plants; 3) wild type vs *NbBT1*-knockout *N. benthamiana* plants, and 4) ethanol solvent vs SA-treated wild type and SLCCNV *BV1*-transgenic *N. benthamiana* plants. Please note that we included the comparison between wild type and *NbBT1*-overexpressing *N. benthamiana* plants as per your comments below. The figures (3C, 3D, 5H and 6F) have been updated with these newly-obtained results.

As for the statistical analysis of whitefly preference data, after reading your advice and conducting normal distribution analysis, we realize that our previous methods (paired samples t-test) is not appropriate. We have thus reanalyzed our data using R package lme4 (version 4.6.0) with Generalized Linear Mixed Model (GLMM) with binomial distribution and logit link function. The methods were described in Statistics and reproducibility of the Materials and Methods section (lines 757-758).

In terms of the host suitability of *N. benthamiana* plants, we agree that its glandular trichomes may trap whiteflies and cause mortality. However, a previous study from our lab showed that *N. benthamiana* plants can effectively attract whiteflies (Han et al. 2024). Here we used *N. benthamiana* because it behaves like the natural host of SLCMV in the regard of SA induction of plant repellence against whitefly. More importantly, considering that *N. benthamiana* is a widely-used model plant in plant-virus interaction, we choose this plant species for analyzing the interaction between SA signaling pathways and begomoviruses or viral proteins.

As for the mechanisms underlying SA-induced plant repellence against whitefly, we agree with you that volatile emissions may play a key role, and profiling of these volatile organic compounds (VOCs) and elucidating their roles in the modulation of plant repellence by SA and BV1 proteins will further clarify the causal chain. However, in this study we focus on the interplays between SA signaling pathway and bipartite begomoviruses, and the modulation of plant antiviral immunity and repellence against whitefly by these interplays. With due respect to the Reviewer, we feel that our study is already complex. The determination and functional characterization of VOCs would add additional complexity to the manuscript that might distract our readers from our focus on the modulation of virus infection and vector preference by the interplays between SA signaling pathway and bipartite begomoviruses. In future endeavors, we will explore the role of VOCs in the modulation of plant repellence by SA and BV1 proteins.

Reference

Han WH, Wang JX, Zhang FB, Ji SX, Zhong YW, Liu YQ, Liu SS, Wang XW. 2024. A new feature of the laboratory model plant *Nicotiana benthamiana*: Dead-end trap for sustainable field pest control. Plants People Planet 6(3): 743-759.

The authors demonstrate that BV1 downregulates NbBT1 transcripts and that BT1 enhances SA signaling and whitefly repellence; however, the mechanism by which BV1 represses BT1 remains unclear. The molecular link downstream of BV1 needs to be elucidated. It is also noteworthy that the study focuses exclusively on BT1 without considering NbL03g16400, despite the potential for functional redundancy between these genes.

**AR:** Thanks for the insightful comment. In this study, we determine that BV1 suppresses *NbBT1* transcription using RNA-seq and qPCR analyses. However, the detailed molecular mechanisms underlying the transcription suppression are unknown. We agree that further dissection of these mechanisms will enriches our understanding of the mode of action of BV1. However, the main focus of our study is the modulation of virus infection and vector preference by the interplays between SA signaling pathway and bipartite begomoviruses. We feel our current results have provided important insights into these interplays and thus decide to conduct and report these explorations in the future. We have thus highlighted this issue and propose future research directions in the Discussion section. See lines 533-540.

As for the functional redundancy of NbL13g16600 and NbL03g16400, here we focus on NbL13g16600 because of two reasons: 1) NbL13g16600 exhibits much higher transcription than NbL03g16400, and 2) while NbL13g16600 transcription is downregulated by BV1 whether SA is sprayed or not, NbL03g16400 transcription is downregulated only in SA-treated plants. We thus feel that NbL13g16600 is more likely to function in the suppression of SA signaling by BV1, which dampens SA signaling in both ethanol solvent and SA-sprayed plants, and in turn choose it for further analysis. After reading your comments, we realize that the role of NbL03g16400 can not be excluded even with these considerations. We have added our considerations and highlighted that functional characterization of NbL03g16400 should be pursued in the future. See lines 523-533.

Reviewer #2: (No Response)

Reviewer #3: None

Part III – Minor Issues: Editorial and Data Presentation Modifications Please use this section for editorial suggestions as well as relatively minor modifications of existing data that would enhance clarity.

Reviewer #1: Please position your claim as a BV1‑specific addition to an already rich framework, and explicitly discuss AC1 vs BV1 antagonism and how these arms coexist temporally.

**AR:** Thanks for the insightful comment. After reading the article of Wang D et al. 2026 (Plant Stress), we realize that both AC1 and BV1 modulate plant attractiveness to whitefly. We have thus added the following contents discussing their roles during coexistence. It should be noted that the role of AC1 and BV1 was determined without considering their interactions. We thus propose a network of bipartite begomovirus-plant-whitefly interactions and urged for more studies (below). See lines 513-520.

When combining our findings with previous reports (Wang D et al. 2026), we propose a network of bipartite begomovirus-plant-whitefly interactions. On the one hand, AC1 and bipartite begomovirus-induced SA accumulation activate plant repellence against whitefly. Since abundant SA accumulates in virus-infected plants, the function of AC1 may be masked by SA. Concurrently, BV1 interferes with SA-induced plant repellence against whitefly, sustaining whitefly preference to virus-infected plants. It is also possible that the modulation of plant attractiveness to whitefly by one viral protein may be affected by the other, and should be examined in future investigation.

Deep functional genetics were conducted in N. benthamiana. Although the study presents assays in cassava (SLCMV) and zucchini (SLCCNV), functional data for BT1/BV1 in these natural hosts are missing. Identifying BT1 orthologs in cassava and zucchini, analyzing their expression levels (±SA and ±BV1), and performing VIGS—if feasible—would significantly strengthen the agronomic relevance of the findings.

**AR:** Thanks for pointing this out. In this study, we explored the function of begomoviral BV1 and plant BT1 in *N. benthamiana* plants. We agree that identification of BT1 orthologs and further functional characterization of BV1 and BT1 in the natural hosts of bipartite begomoviruses will improve the agronomic relevance of the findings. However, since we did not work much on cassava and zucchini plants, related experimental systems such as gene cloning and silencing have not been established, preventing further dissection of this issue within a short period of time. We have acknowledged this limitation in our manuscript and urged for further investigations. See lines 33, 443-446, 556-559.

Reviewer #2:

The manuscript presents a comprehensive and mechanistically detailed study demonstrating how the bipartite begomoviral protein BV1 subverts SA signaling by downregulating NbBT1 to promote both viral infection and vector-mediated transmission. The work is compact with necessary validation demonstrated when and where required, and the use of multiple pathosystems, transgenic lines, and behavioral assays strengthens the conclusions. However, several gaps remain including missing controls (DNA-B alone, ethanol-treated BV1-transgenic RNA-seq), overclaimed causality (PAL pathway, DNA-B dependence), and incomplete datasets (NbBT1-overexpression repellence assays, silencing efficiency data) that require clarification or additional experimentation. With significant revisions addressing these points as suggested, the manuscript will be suitable for acceptance.

**AR:** Thank you for the positive assessment of our study and the insightful comments. We have performed additional experiments and revised the manuscript as per your comments. Detailed responses to your comments are provided below.

INTRODUCTION

Lines 86-96 : The authors state that "whether and how the interplay between viruses and plant antiviral defenses modulates plant attractiveness to arthropod vectors remains largely unknown." This claim requires revision, as several studies have already established direct mechanistic links. For instance, Liang et al. (2025) demonstrated that TYLCV upregulates JA-associated terpene synthase genes in tomato; CRISPR knockout of these genes abolished both volatile emissions and whitefly attraction, proving that virus-modified defense gene expression directly controls vector preference. Similarly, Shi et al. (2019) used JA-pathway mutants to show that viruliferous whiteflies suppress JA biosynthesis in tomato, reducing defense volatiles and increasing plant attractiveness, explicitly linking viral modulation of phytohormone defenses to vector behaviour. The field has moved beyond asking whether such interplay exists to understanding how it is mechanistically executed. The authors should revise this sentence to reflect current knowledge while identifying genuine remaining gaps.

**AR:** Thanks for the insightful comment and references provided. We agree with you that our previous claim is inappropriate as several relevant reports are available. We have thus searched for relevant publications and in turn changed our claim to the sentences listed below in the manuscript (see lines 100-107).

In the last decades, dozens of case studies have been reported on the modulation of whitefly preference by whitefly-borne begomoviruses (Zhao et al. 2022; Liang et al. 2025; Wang D et al. 2026; Wang SD et al. 2026). While several studies have been conducted to dissect the plant and viral factors involved, mechanistic insights were provided in only a few studies (Li et al. 2014; Shi et al. 2019; Liang et al. 2025; Wang D et al. 2026; Wang SD et al. 2026). For example, jasmonate signaling pathway is often modulated by begomoviruses for the manipulation of whitefly preference (Li et al. 2014; Shi et al. 2019; Wang D et al. 2026; Wang SD et al. 2026). Under this scenario, the identification of additional plant and viral factors, and more importantly, the molecular interplays between these factors governing the modulation of whitefly preference by begomoviruses, will significantly expand our understanding of begomovirus-whitefly interactions and virus-vector interactions in general.

Reference

Li R, Weldegergis BT, Li J, Jung C, Qu J, Sun Y, Qian H, Tee C, van Loon J, Dicke M. 2014. Virulence factors of geminivirus interact with MYC2 to subvert plant resistance and promote vector performance. Plant Cell 26: 4991-5008.

Liang P, Zeng Y, Ning J, Wu X, Wang W, Ren J, Wu QJ, Yang X, Wang SL, Guo ZJ, Su Q, Zhou XG, Turlings TCJ, Xie W, Zhang YJ. 2025. A plant virus manipulates both its host plant and the insect that facilitates its transmission. Science Advances 11(9): eadr4563.

Shi XB, Preisser EL, Liu BM, Pan HP, Xiang M, Xie W, Wang SL, Wu QJ, Li CY, Liu Y, Zhou XG, Zhang YJ. 2019. Variation in both host defense and prior herbivory can alter plant-vector-virus interactions. BMC Plant Biology 19(1): 556.

Wang D, Zhang X, Liu Q, Zhang P, Ye J. 2026. Begomovirus AC1 protein switches sides to boost plant defense against whiteflies. Plant Stress 19: 101134.

Wang SD, Zhao SX, Wang XW, Liu SS, Pan LL, Liu YQ. 2026. Geminiviral C2 proteins promote virus transmission by subverting plant defenses upon infestation of non-vector herbivores. Pest Management Science 82(5): 4419-4428.

Zhao K, Liu SS, Wang XW, Yang JG, Pan LL. 2022. Manipulation of whitefly behavior by plant viruses. Microorganisms 10(12): 2410.

General Comment : More broadly, the Introduction section would benefit from restructuring. Rather than framing this research area as largely uncharted, the authors should first acknowledge and cite the growing body of mechanistic studies that have already established clear connections between virus-defense interplay and vector attractiveness. This would provide necessary context and demonstrate the authors' command of the field. Following this, the authors can then identify the specific gaps their work addresses such as unresolved molecular mechanisms, conserved signalling nodes, or host counter-manipulation strategies thereby positioning their contribution within an active, advancing research frontier rather than an unexplored one.

**AR:** Thank you for the guidance in improving our Introduction section. Indeed, the original introduction is too broad and many relevant studies are not properly cited. Following your advice, we have restructured the second and third paragraphs so as to clearly point out the knowledge gaps. In the second paragraph, we directly put our focus on the interactions between SA signaling pathway and begomoviruses, and identify the key knowledge gap, namely the induction and subversion of plant SA signaling pathway by bipartite begomoviruses (lines 80-91). In the third paragraph, we first acknowledge that a handful of factors have been identified and functionally characterized in the modulation of vector preference and then highlight that more investigations on plant and viral factors and their molecular interplays are warranted (lines 107-117).

MATERIAL and METHODS

Lines 419-424, 432-435 ; Please provide details on the Agrobacterium strain used for generating the SLCMV BV1 and NbBT1 overexpression lines. For the NbBT1 CRISPR lines, please specify the Cas9 variant, the promoter driving Cas9 expression, and the method used to confirm homozygosity beyond Sanger sequencing.

**AR:** Thanks for pointing this out. The *Agrobacterium tumefaciens* strain GV3101 was used for all plant transformations, including the generation of both the overexpression and CRISPR/Cas9 lines. The *NbBT1* CRISPR lines were generated using the K5-KRSN-ccdB vector, which employs a plant-codon-optimized *Streptococcus pyogenes* Cas9 (SpCas9) variant under the control of the CaMV 35S promoter. When revising the manuscript as per your advice, we noticed that in previous version the vector for generating CRISPR lines is incorrect and have thus corrected it. To confirm the homozygosity of the edited lines, we performed Sanger sequencing of PCR-amplified target regions of *NbBT1* in the plants of T0 and T1 generations, ensuring the selection of stable, non-mosaic homozygous mutants. These details have now been integrated into the Methods section of the revised manuscript. See lines 583-592.

Lines 477-484 ; Please provide more details on the PAL enzyme activity assay, including the full kit name, manufacturer, and catalogue number. Additionally, please clarify whether the assay was validated for the plant species tested (N. benthamiana, cassava, zucchini) and provide information on controls, background absorbance, and assay linearity.

**AR:** The activity of phenylalanine ammonia-lyase (PAL) was quantified using the Phenylalanine Ammonia-lyase (PAL) Activity Assay Kit (UV Colorimetric Method; Cat #D799599, Sangon Biotech, Shanghai, China). Blank controls that were used to eliminate background absorbance were provided within the kit and used as per the manual. To ensure the assay was reliable for the plant species tested (*N. benthamiana*, cassava, and zucchini), we first prepared leaf extracts from control and virus-infected plants of three species and made serial dilutions. After treatment following the kit manual, we analyze the absorbance at 290 nm and found that results of the extracts from 0.1 g of leaves were within the linear range of this assay. We thus used 0.1 g of leaf tissues in all analysis. We have added this information in the Methods to improve clarity. See lines 662-675.

Several commercial reagents are cited without catalogue numbers (e.g., Easy Plant Genomic DNA Extraction Kit, RNA extraction kit from Accurate Biology, Evo M-MLV RT Kit).

**AR:** We thank the reviewer for pointing this out. The catalogue numbers for all commercial reagents mentioned, including the Easy Plant Genomic DNA Extraction Kit, the RNA extraction kit (Accurate Biology), and the Evo M-MLV RT Kit, have been added to the "Materials and Methods" section of the revised manuscript to ensure reproducibility. See lines 639-640, 652-657, 663, 678, 685, and 724-730.

Lines 523-525; The authors state: "Apical leaves from three N. benthamiana plants were pooled as one biological sample." However, the total number of biological replicates per treatment for RNA-seq is not clearly stated. This information is essential for assessing the statistical power of the transcriptomic analysis.

**AR:** In RNA-seq, three independent biological replicates were used for each treatment. We have added this information in the Methods sections. See line 723.

Lines 513-521; Please provide additional details on the VIGS experiments: the specific region of NbBT1 targeted (e.g., 5'UTR, CDS, or 3'UTR), the time point post-infiltration when silencing was assessed, the tissues sampled, how silencing efficiency was evaluated and what criteria were used to define acceptable silencing, and the number of independent VIGS experiments performed.

**AR:** Thanks for pointing this out. We have now specified that the target regions were within the *NbBT1* CDS (69-368 and 582-881), clarified the time point post-infiltration when silencing was assessed (one days post the last spray of SA) and methods (RT-PCR), and defined our criteria for successful silencing (a reduction of >70%). Additionally, we specified that six samples (apical systemic leaves from three plants) were harvested for the analysis of *NbBT1*, *NbPR1a* and *NbPR2* transcription. We have updated the Methods section to clarify these concerns. See lines 707-718. As stated in the Statistics and reproducibility section, VIGS experiments were conducted twice and only data from representative experiments were shown (lines 764-766).

Lines 550-557; Please provide details on whether data from independent experiments were pooled or if representative experiments are shown, specify the methods used to assess normality and homogeneity of variance prior to parametric testing, and indicate whether any corrections for multiple comparisons were applied.

**AR:** Thanks for bringing this to our attention. In this study, all experiments described were repeated at least once with similar results, and data from representative experiments are shown. No correction of data was applied. After reading your comment, we performed normal distribution analysis for all data using Shapiro-Wilk test. We found that all data of phytohormone content and relative PAL enzyme activity, and the majority of data of relative mRNA levels (84 out of 94 cases), and thus Levene's test for equality of variances and two-sided Student’s independent t-test were employed. Please note that *P* values of t-test were determined according to the results of Levene's test. Additionally, non-parametric Mann-Whitney U tests were used for the data of virus quantity as in more than one third (25 out of 69) cases they did not follow normal distribution. As for whitefly preference data, we analyze them using R package lme4 (version 4.6.0) with Generalized Linear Mixed Model (GLMM) with binomial distribution and logit link function. These details have been updated in the manuscript, see lines 749-766.

RESULTS:

Lines 109-160 ; In the first result section "SLCMV induces SA accumulation via the PAL pathway in a DNA-B-dependent manner":

The conclusion that SA induction is "DNA-B-dependent" is not fully supported, as DNA-B alone controls are missing. The current data only show that DNA-A+DNA-B induces more SA than DNA-A alone, not that DNA-B is sufficient or directly responsible. Please clarify whether DNA-B-only inoculations were attempted or revise the claim accordingly. The striking difference in SA accumulation between SLCMV (~37-fold) and other begomoviruses like TYLCV (~5-10 fold) is not addressed. Was viral titer comparable at the time of sampling? Were the same Agrobacterium strain and vector backbone used for all constructs? Please clarify whether differential infection efficiency or experimental conditions could explain this disparity. The claim that SA accumulation occurs "via the PAL pathway" remains correlational. PAL genes are upregulated and enzyme activity increases, but no loss-of-function evidence (PAL inhibitors, silencing) is provided to demonstrate causality. Please either temper this conclusion or provide additional support. Additionally, no true mock control (buffer-only or untransformed agrobacteria) is included. Since Agrobacterium infiltration itself triggers SA responses, this is a critical omission particularly when comparing across viruses.

Recommendation: Revise this subsection to reflect that successful systemic infection (requiring DNA-B) is associated with high SA and PAL activation, rather than claiming a direct, DNA-B-dependent, PAL-mediated mechanism.

**AR:** Thanks for the guidance in interpreting our results. After reading your comments, we realize that our previous claim that SLCMV induces SA accumulation via the PAL pathway in a DNA-B-dependent manner is inappropriate. We agree that "DNA-B-dependent" is not fully supported, because effect of DNA-B alone on SA contents was not determined as DNA-B alone can not infect plants. We have clarified this point in the manuscript (lines 183-185). We have also deleted ‘in a DNA-B-dependent manner’ in the titles of this section and figure legends since we did not directly determine the role of DNA-B (lines 133, 968).

As for the role of PAL pathway in SLCMV-induced SA accumulation, we agree with you that previous data did not clearly demonstrate the causality. We have thus conducted new experiments using AIP (2-aminoindan-2-phosphonic acid), a strong competitive inhibitor of PAL enzymes (Appert et al. 2003). We found that pharmacological inhibition of PAL significantly attenuates the SLCMV-induced SA accumulation, thereby providing functional evidence for this pathway's involvement. We have added and described this data in the manuscript (see lines 168-172 and Fig. S5A). Even with these results, we feel we can only conclude that PAL pathways contribute to SLCMV-induced SA accumulation, but can not exclude the involvement of other pathways. We have thus deleted ‘via the phenylalanine ammonia-lyase (PAL) pathways’ in the titles of this section and figure legends (lines 132, 967).

Regarding the induction of SA accumulation, we first exclude the effect of agrobacteria infiltration by comparing SA contents in plants inoculated with infiltration buffer, untransformed agrobacteria and agrobacteria containing pBINPLUS (empty vector). We found no significant difference in SA contents among the three kinds of plants, thereby excluding the possibility that the infiltration of agrobacteria alone or agrobacteria containing pBINPLUS induce SA accumulation. We have updated the Results section, see lines 151-156 and Fig. S3. When comparing SA accumulation induced by begomoviruses and begomovirus-betasatellite complexes, we used the same agrobacterium strain, inocula OD value and vector backbone (pBINPLUS). The details of infectious clones, agrobacteria strain and solutions were given in the Methods section (lines 603-609, 623-627). As for the disparity in inducing SA accumulation, we propose several reasons such as differential virus accumulation and symptoms. We have incorporated these considerations in the Discussion sections (lines 388-390).

Reference

Appert C, Zoń J, Amrhein N. 2003. Kinetic analysis of the inhibition of phenylalanine ammonia-lyase by 2-aminoindan-2-phosphonic acid and other phenylalanine analogues. Phytochemistry 62: 415-422.

Lines 162-201 ; In the second result section "SLCMV DNA-B and BV1 mitigate SA-induced antiviral defenses" ; There is an apparent contradiction regarding BV1's role: Figure 1G and lines 148-158 show that both BC1 and BV1 are required for SA accumulation, whereas lines 193-200 and Figure 2E-F show that BV1 does not affect SA levels but suppresses SA signaling. This creates confusion. Does BV1 contribute to inducing SA, suppressing its signaling, or both? The distinction is buried and should be explicitly clarified: BC1 and BV1 are likely required for systemic infection (thereby indirectly enabling SA induction), while BV1 alone directly suppresses SA signaling. Please revise the text to separate these two distinct functions and avoid any perceived contradiction.

**AR:** We appreciate the reviewer’s keen observation regarding the dual roles of BV1. We propose that like the well-studied βC1 protein, BV1 may play multiple roles in virus-plant interactions. BV1 may promote SA accumulation indirectly by promoting virus pathogenesis and at the same subvert SA signal transduction to sustain virus infection and vector preference. We have reorganized the Discussion and used one paragraph (see below and lines 451-472) to discuss the seemingly contrasting role of BV1 in the interactions between plant SA signaling pathway and SLCMV.

In this study, we uncover two seemingly contrasting roles of BV1 proteins in SLCMV interaction with plant SA signaling pathway. On the one hand, BV1 is required for the induction of SA accumulation by SLCMV as mutation of the ORF encoding this gene abolishes SLCMV-induced SA accumulation and at the same significantly impaired SLCMV DNA-A accumulation and symptom development. On the other hand, BV1 subverts SA signaling as it markedly suppresses transcription of SA downstream genes while does not affect SA content in plants. We propose that the two roles of BV1 are not conflicting as they occur in different processes. During SLCMV infection, BV1 may mediate the nuclear export of viral DNAs (Noueiry et al. 1994), and in turn promote virus systematic infection and symptom development, thereby indirectly contributing to SA accumulation. At the same time, BV1 suppresses the downstream signaling of SA, thereby sustaining virus infection and plant attractiveness to whitefly. In addition to acting as a suppressor of SA signaling, BV1 proteins have also been shown to suppress transmembrane receptor kinase-mediated antiviral defenses, post-transcriptional gene silencing, and jasmonate signaling (Fontes et al. 2004; Li et al. 2014; Ye et al. 2015). It seems that BV1 is similar to βC1 encoded in betasatellites associated with begomovirus, which promotes virus infection and at the same time dampens multiple plant antiviral defenses (Li et al. 2019). The identification of BV1 as a suppressor of SA signaling in our study, further expands our understanding of the multifaceted roles of bipartite begomoviral proteins in counteracting plant antiviral defenses.

Reference

Fontes EPB, Santos AA, Luz DF, Waclawovsky AJ, Chory J. 2004. The geminivirus nuclear shuttle protein is a virulence factor that suppresses transmembrane receptor kinase activity. Genes Dev. 18: 2545-2556.

Li FF, Yang XL, Bisaro DM, Zhou XP. 2018. The βC1 protein of geminivirus-betasatellite complexes: A target and repressor of host defenses. Molecular Plant 11(12): 1424-1426.

Noueiry AO, Lucas WJ, Gilbertson RL. 1994. Two proteins of a plant DNA virus coordinate nuclear and plasmodesmal transport. Cell 76: 925-932.

Ye J, Yang J, Sun Y, Zhao P, Gao S, Jung C, Qu J, Fang R, Chua NH. 2015. Geminivirus activates ASYMMETRIC LEAVES 2 to accelerate cytoplasmic DCP2-mediated mRNA turnover and weakens RNA silencing in Arabidopsis. PLoS Pathogens 11: e1005196.

Lines 203-225 ; In the section “SLCMV DNA-B and BV1 interfere with SA-induced plant repellence against whitefly” ; While this section is generally clear, one discrepancy should be addressed. In Figure 3C, the authors test whitefly preference on plants inoculated with SLCMV A, A+B, A+B-mBC1, and A+B-mBV1 following SA or ethanol treatment. However, no control is included for plants inoculated with DNA-B alone or DNA-B-mBC1/mBV1 alone (without DNA-A). This makes it difficult to determine whether the observed effects on repellence require active viral infection or whether BV1 expression alone is sufficient. Additionally, the order of treatments (inoculation first, then SA spray, then choice assay) is not clearly stated in the legend and should be explicitly described to aid interpretation.

AR: Thank you for bring this to our attention. We did not include plant infected with DNA-B or DNA-B-mBC1/mBV1 alone because these DNA-Bs (either wild type or mutant) can not establish infection without DNA-A. This information has been added in the manuscript as per your previous comments. See lines 183-185. We agree that in this case, it is difficult to determine the exact role of BV1. We have thus generated SLCMV *BV1*-transgenic *N. benthamiana* plants to clearly show the role of BV1 (see Fig. 3D). As for the detail of treatments, we have updated the legends to aid interpretation. See lines 1021-1024, 1027-1030.

Lines 227-254 ; In the section “SLCMV BV1 downregulates the transcription of BTB/POZ and TAZ domain-containing protein 1 (NbBT1), an SA-inducible gene” ;

Several discrepancies and missing controls should be addressed: 1. Missing control group: The RNA-seq experimental design (Figure 4A-F) compares SA-treated wild-type, SA-treated BV1-transgenic, and ethanol-treated wild-type plants. However, ethanol-treated BV1-transgenic plants were not included. This omission makes it impossible to determine whether BV1 downregulates NbBT1 independently of SA treatment or only in the presence of SA. Please explain why this control was omitted or provide additional data.

**AR:** Thank you for the careful assessment of our experiments and insightful comment. In RNA-seq experiment, initially we used four treatments, namely ethanol-treated wild type plants, ethanol-treated *BV1*-transgenic plants, SA-treated wild type plants and SA-treated *BV1*-transgenic plants. We omitted the treatment of ethanol-treated *BV1*-transgenic plants in previous version of our manuscript because of our hypothesis, namely that the genes mediating the suppression of SA signaling by BV1 may be upregulated by SA treatment and downregulated by BV1. Following this strategy, we conducted comparisons only between ethanol solvent- and SA-treated wild type plants, and SA-treated wild type and SA-treated BV1-transgenic plants. After reading your comment, we realize that the previously-omitted treatments should be included and have thus revised Fig. 4F. We have also edited some statements in the text so that readers can understand our logic flow. See lines 274-279, 294-300.

2. Figure 4G-H discrepancy: Figure 4G shows that SA induces NbBT1 transcription in a dose-dependent manner in wild-type plants. Figure 4H then shows that BV1 significantly reduces NbBT1 transcription under both ethanol and SA treatment. However, the ethanol-treated BV1-transgenic sample in Figure 4H has no counterpart in the RNA-seq analysis, creating inconsistency between the validation experiment and the transcriptomic dataset.

**AR:** Thanks for pointing this out. Following your previous comment, we have included the treatment of ethanol-treated *BV1*-transgenic plants in the analysis of transcripts in RNA-seq data analysis (See revised Fig. 4F). The suppression of *NbBT1* (*NbL13g16600*) transcription by BV1 upon both ethanol solvent and SA treatments is now consistent in RNA-seq (Fig. 4F) and qPCR validation (Fig. 4H) experiments.

3. Incomplete validation: The authors identify two SA-responsive BT1 family genes (NbL03g16400 and NbL13g16600) from RNA-seq but only validate NbL13g16600 (NbBT1). No explanation is provided for why NbL03g16400 was not pursued, nor whether it shows similar regulation by BV1.
**AR:** We appreciate your comment. Following our hypothesis and screening strategy, we identify two genes NbL03g16400 and NbL13g16600 that are modulated by both SA and BV1. We then analyze the abundance of their transcripts in RNA-seq samples and found that while the transcription of both genes is downregulated by BV1 upon SA treatment, only NbL13g16600 is downregulated by BV1 in ethanol solvent-treated plants. Under this scenario, we choose NbL13g16600 (renamed as *NbBT1*) for further analysis as it is more likely to function in the suppression of SA signaling by BV1, which dampens SA signaling in both ethanol solvent and SA-sprayed plants. We have added these considerations in the Results (lines 294-300) and Discussion (lines 523-533) sections.

4. Discrepancy between FPKM and qPCR data: In Figure 4F, NbL13g16600 FPKM in SA-treated BV1-transgenic plants appears reduced compared to SA-treated wild-type but is still higher than ethanol-treated wild-type. In Figure 4H, qPCR shows BV1 reduces NbBT1 levels to below ethanol-treated wild-type baseline. This quantitative discrepancy should be addressed.

**AR:** Thanks for the careful assessment of our data. After reading your comments, we have reexamined *NbBT1* transcription levels in SA-treated *BV1*-transgenic and ethanol-treated wild type plants in RNA-seq and qPCR experiments (see the table below). Statistical analysis shows that while transcript abundance of *NbBT1* is significantly higher in SA-treated *BV1*-transgenic line 2 than ethanol-treated wild type in RNA-seq experiment, in qPCR experiment no significant difference between ethanol-treated wild type and SA-treated *BV1*-transgenic plants is found. Therefore, although there are slight differences in the results between the two experiments, they are not conflicting.

|  | Transcript abundance in RNA-seq | Transcription in qPCR |
| --- | --- | --- |
| ethanol-treated wild type | 84.64, 72.71, 79.85 | 0.119, 0.193, 0.143, 0.118, 0.154, 0.139 |
| SA-treated *BV1*-transgenic line 2 | 92.59, 103.97, 105.99 (significantly higher) | 0.102, 0.182, 0.078, 0.120, 0.057, 0.060 (not significantly different) |
| SA-treated *BV1*-transgenic line 4 |  | 0.046, 0.067, 0.120, 0.077, 0.197, 0.181 (not significantly different) |

It should be noted that the main information we aim to provide in Figs. 4F and 4H is that BV1 downregulates *NbBT1* transcription upon both ethanol solvent and SA spray. This is consistent in the results of two experiments. Following your previous comments, we have added the results of ethanol-treated *BV1*-transgenic plants in Fig. 4F. To direct readers’ attention to our main conclusion, we have presented comparisons only between wild type and *BV1*-transgenic plants (see revised Fig. 4F).

Please clarify these points and provide appropriate justification or additional data.

"NbBT1 positively regulates plant SA signaling, antiviral defenses and plant repellence against whitefly"

• Figure 5H shows whitefly preference for NbBT1-knockout over wild-type plants, but no corresponding assay with NbBT1-overexpression lines is shown. This omission leaves the gain-of-function evidence for repellence incomplete.

**AR:** Thank you for bring this to our attention. We have conducted whitefly preference assay with increased replicates (10) using wild type, *NbBT1*-knockout and overexpression plants. Fig. 5H has been updated and results were described in lines 317-320.

"NbBT1 is required for the suppression of SA signaling by SLCMV BV1"

• VIGS silencing efficiency data for NbBT1 are referenced but not shown anywhere in the main text or supplementary files. Please provide these data.

**AR:** Thank you for pointing this out. We have added the description of gene silencing efficiency in manuscript (lines 325-326) and Fig. S13.

• The time point for PR gene expression analysis post-VIGS is not specified in the legend or methods.

**AR:** Thank you for pointing this out. We have added the time points in methods (lines 714-716) and legends (lines 1079-1080).

"SLCCNV induces SA accumulation and SLCCNV BV1 interferes with SA signaling"

• Figure 6D uses SLCMV DNA-A as a proxy to test SLCCNV BV1 function in N. benthamiana. The authors should explicitly acknowledge this as a limitation, as the two BV1 proteins are not identical and may function differently.

**AR:** Thank you for pointing this out. We have added some contents in Discussion section to acknowledge this limitation (lines 438-441).

• Figure 6I shows SLCCNV BV1 downregulates NbBT1, but no mechanistic data are provided to show this occurs via the same pathway as SLCMV BV1 ; this is assumed rather than demonstrated.

**AR:** Thanks for pointing this out. In Fig. 6I we showed that the transcription of *NbBT1* was downregulated by SLCCNV BV1, a finding that was similar to SLCMV BV1. Regarding the modes of action of two BV1 proteins, we agree with you that they may function differently in modulating *NbBT1* transcription since we did not explore the underlying mechanisms. We have revised the Discussion section to acknowledge this limitation and urge for further investigations (lines 533-540).

DISCUSSION

The discussion is well-structured but currently assumes all conclusions are fully validated and will require revision once the authors address the experimental gaps noted above. Specifically, the claim that SA accumulation is DNA-B-dependent should be tempered if DNA-B-only controls cannot be provided, and the assertion that SLCMV induces SA via the PAL pathway should be revised to reflect that causality has not been experimentally demonstrated. The discussion should also explicitly separate BV1's indirect role in enabling systemic infection from its direct role in suppressing SA signaling, as these are currently conflated. Additionally, the statement that BV1-mediated suppression is conserved across bipartite begomoviruses is based on only two viruses and should be softened to "observed in another bipartite begomovirus" unless additional evidence is provided. Finally, if missing controls or assays (such as ethanol-treated BV1-transgenic RNA-seq or NbBT1-overexpression repellence assays) are not added, the corresponding conclusions should be appropriately qualified. The authors should revisit the discussion after addressing these experimental gaps to ensure conclusions align with the strength of the evidence.

**AR:** Thanks for the guidance in revising our Discussion section. As for the role of DNA-B and its encoded proteins in SLCMV-induced SA accumulation, we have updated the discussion section to acknowledge that DNA-B-only treatment can not be provided. See lines 403-414. As for PAL pathway, we agree with you that its role in SLCMV-induced SA accumulation has not been experimentally demonstrated. We have analyzed the effect of AIP treatment and updated the discussion section with the results obtained. Moreover, we have tune down the statements as more investigations are warranted to unambiguously resolve this issue. See lines 414-422.

As for the role of BV1 in SLCMV interactions with plant SA signaling pathway, we have included a whole paragraph to discuss its seemingly contrasting role. See lines 451-472. With regard to the statements on bipartite begomoviruses, we agree they should be tuned down as only two bipartite begomoviruses were analyzed. We have thus revised the discussion (lines 371, 374, 396, 508, 563, 565) and also the abstract (line 36).

As for the inclusion of ethanol-treated SLCMV *BV1*-transgenic plants in RNA-seq analysis and *NbBT1*-overexpressing plants in whitefly preference assay, we have added the data in the manuscripts (Figs. 4F and 5H).

Reviewer #3: This paper investigates how a whitefly-transmitted begomovirus manipulates host plants and their defenses for their infection cycle and transmission. By investigating of two species of begomoviruses the authors show that plant infection with Sri Lankan cassava mosaic virus (SLCMV) induces the accumulation of salicylic acid SA, a positive regulator of antiviral defense. As a responses, SLCMV DNA-B and the BV1 protein interfere with SA-induced antiviral defenses and SA signal transduction. Additionally, SA induces plant repellence to whitefly vectors and this repellence is mitigated by SLCMV DNA-B and BV1. This response is achieved by BV1 downregulating the transcription of BTB/POZ and TAZ domain-containing protein 1 (BT1), a positive regulator of SA signal transduction, plant antiviral defenses and repellence against whitefly.

The manuscript show strong evidence for the arms race between virus infection and the plant defenses activated following infection. The manuscript is solid but would appreciate some clarifications:

**AR:** Thank you for the positive assessment. Detailed responses to your comments are provided below.

1. The pharmacological treatment for manipulating the amount of SA using AIP. Are there any side effects on other molecular pathways that the authors are not aware about? How they can be sure about that? Is there maybe any effect on the virus itself? Can AIP be applied to other plant system to confirm this result?

AR: We thank the reviewer for raising these important technical considerations regarding the specificity and application of AIP. In this study, we explored the role of SA in plant antiviral defenses using *NahG*-transgenic *N. benthamiana* plants and AIP spray in two species of plants (*N. benthamiana* and zucchini). Results from these experiments consistently showed that inhibiting SA accumulation significantly impaired plant antiviral defenses. We agree with you that AIP spray may exhibit side effects on other plant signaling pathways and the viruses themselves, and we thus analyzed the effect of AIP spray on the contents of SA and other plant hormones and the effect of AIP spray on plant antiviral defenses in *NahG*-transgenic *N. benthamiana* plants.

In both control and SLCMV-infected *N. benthamiana* plants, AIP treatment significantly decreased the contents of SA, but did not significantly affect the contents of JA, JA-Ile, OPDA or ABA (Figs. S5). Additionally, while in wild type plants AIP treatment significantly promoted SLCMV infection, in *NahG*-transgenic plants no significant difference in SLCMV DNA-A titers was found between DMSO and AIP-treated plants (Fig. S8). These findings show that the effect of AIP on SLCMV infection is attributable to its effect on SA accumulation, but not other plant pathways or the virus itself. These results were described in the manuscript (lines 206-212).

When reading your comments, we realize that the legends of Figs. 2A-B is not clear enough. These legends may lead to the mistaken conclusion that AIP treatment is more effective than *NahG* transgene on SLCMV infection. Actually, both AIP treatment and *NahG* transgene modulates SLCMV infection after 20 days post inoculation. In Fig. 2A, plants of two kinds were inoculated with SLCMV A+B and then sampled for analysis at 10, 20, 30 and 40 days post inoculation, and significant difference was found at 20, 30 and 40 days post inoculation. In Fig. 2B, wild type plants were inoculated with SLCMV A+B and at 10 days post inoculation plants were sprayed with DMSO or AIP for three consecutive days. Sampling was conducted at 10, 20 and 30 days post the last spray (23, 33 and 43 days post inoculation), and significant difference was found at all time points analyzed. We have revised the legends (lines 990-997) and text (lines 205-206) to avoid confusion.

2. In line 173 the authors assume that only SA functions in suppressing the ability of the virus to replicate and accumulate in the plant, while they didn't explore all possible mechanisms in the plant that might do that. The assumption that the virus might respond to the SA activity is OK, however they need to emphasize the fact that other unexplored mechanisms may exist.

**AR:** We appreciate the reviewer’s perspective that host antiviral defense is a multi-layered process involving numerous mechanisms beyond the SA signaling pathway. Here we focus on SA as it is a key regulator of plant antiviral defenses, and more importantly, SA accumulates abundantly in SLCMV-infected plants. We agree that other pathways may also play important roles in modulating the replication and accumulation of SLCMV in the plants, and thus should be subjected to further investigations. We have revised the Discussion section to incorporate this notion. See lines 398-401.

3. In the section dealing with the BV1 interference with the SA pathway, the authors performed a transcriptomic analysis to identify gene regulated by BV1, and ended up with two BTB/POZ gene, one was chosen for further analysis and was regulated by BV1. My question is if the authors initially assumed that the SA would be regulated by BV1 and they were going for this direction, why they didn't focus on this group of genes, and went for full transcriptomic analysis?

**AR:** We thank the reviewer for the comment regarding our experimental strategy. In Figs. 1, 2 and 3, we determined that SLCMV BV1 interferes with SA signaling, SA-mediated antiviral defenses and SA-mediated plant repellence against whitefly. Therefore, in Fig. 4 we focused on the molecular modes of action of SLCMV BV1. We did not predetermine that NbBT1 is a key factor in the modulation of SA signaling by SLCMV BV1, and thus did not focus on this group of genes in the beginning. However, we do have a clear strategy, namely identifying the plants genes that were upregulated by SA treatment and downregulated by BV1. Following this strategy, we performed full transcriptomic analysis, and identified two genes that met our criteria. The process has been described in detail in the Results section to facilitate understanding (lines 274-300).
