# Supplementary material for: Subversion of the salicylic acid signaling pathway by the bipartite begomoviral protein BV1 promotes virus infection and vector preference to virus-infected plants
Source: PLoS Pathog. 2026 Jul 7;22(7):e1014354. doi: 10.1371/journal.ppat.1014354 (PMC13340803; doi:10.1371/journal.ppat.1014354)
Supplement: Peer review history

# Peer review: Decision Letter 0

**DOI:** 10.1371/journal.ppat.1014354.r001

**Authors:** Shou-Wei Ding, John Carr

13 Apr 2026

-->PPATHOGENS-D-26-00197

Subversion of the salicylic acid-signaling pathway by the bipartite begomoviral protein BV1 promotes virus infection and vector-mediated transmission

PLOS Pathogens

Dear Dr. pan,

Thank you for submitting your manuscript to PLOS Pathogens. After careful consideration, we feel that it has merit but does not fully meet PLOS Pathogens's publication criteria as it currently stands. Therefore, we invite you to submit a revised version of the manuscript that addresses the points raised during the review process.

Please submit your revised manuscript by Jun 12 2026 11:59PM. If you will need more time than this to complete your revisions, please reply to this message or contact the journal office at plospathogens@plos.org. When you're ready to submit your revision, log on to https://www.editorialmanager.com/ppathogens/ and select the 'Submissions Needing Revision' folder to locate your manuscript file.

Please include the following items when submitting your revised manuscript:

* A letter that responds to each point raised by the editor and reviewer(s). You should upload this letter as a separate file labeled 'Response to Reviewers'. This file does not need to include responses to any formatting updates and technical items listed in the 'Journal Requirements' section below.

* A marked-up copy of your manuscript that highlights changes made to the original version. You should upload this as a separate file labeled 'Revised Manuscript with Track Changes'.

* An unmarked version of your revised paper without tracked changes. You should upload this as a separate file labeled 'Manuscript'.

If you would like to make changes to your financial disclosure, competing interests statement, or data availability statement, please make these updates within the submission form at the time of resubmission. Guidelines for resubmitting your figure files are available below the reviewer comments at the end of this letter.

We look forward to receiving your revised manuscript.

Kind regards,

John P Carr

Academic Editor

PLOS Pathogens

Shou-Wei Ding

Section Editor

PLOS Pathogens

Sumita Bhaduri-McIntosh

Editor-in-Chief

PLOS Pathogens

orcid.org/0000-0003-2946-9497

Michael Malim

Editor-in-Chief

PLOS Pathogens

orcid.org/0000-0002-7699-2064

**Journal Requirements:**

1) Please ensure that the CRediT author contributions listed for every co-author are completed accurately and in full.

At this stage, the following Authors/Authors require contributions: Guan-Ping Chen, Di Li, Xing Zhang, Ming-Yang Yu, Jing-Ru Zhang, Wilmer J. Cuellar, Xiao-Wei Wang, Shu-Sheng Liu, Yin-Quan Liu, and Li-Long pan. Please ensure that the full contributions of each author are acknowledged in the "Add/Edit/Remove Authors" section of our submission form.

The list of CRediT author contributions may be found here: https://journals.plos.org/plospathogens/s/authorship#loc-author-contributions

2) Please upload all main figures as separate Figure files in .tif or .eps format. For more information about how to convert and format your figure files please see our guidelines:

https://journals.plos.org/plospathogens/s/figures

3) We have noticed that you have uploaded Supporting Information files, but you have not included a list of legends. Please add a full list of legends for your Supporting Information files after the references list.

4) Please amend your detailed Financial Disclosure statement. This is published with the article. It must therefore be completed in full sentences and contain the exact wording you wish to be published.

- State the initials, alongside each funding source, of each author to receive each grant. For example: "This work was supported by the National Institutes of Health (####### to AM; ###### to CJ) and the National Science Foundation (###### to AM)."

- State what role the funders took in the study. If the funders had no role in your study, please state: "The funders had no role in study design, data collection and analysis, decision to publish, or preparation of the manuscript.".

If you did not receive any funding for this study, please simply state: u201cThe authors received no specific funding for this work.u201d

5) Figures: S1, S4, S5, S6, and S9: Please confirm (a) that you are the photographer; or (b) provide written permission from the photographer to publish the photo(s) under our CC BY 4.0 license.

**Reviewers' Comments:**

Reviewer's Responses to Questions

**Part I - Summary**

Please use this section to discuss strengths/weaknesses of study, novelty/significance, general execution and scholarship.

Reviewer #1: The authors demostrate that SLCMV induces SA accumulation via the PAL pathway, and that the DNA‑B–encoded BV1 (NSP) protein suppresses SA‑induced antiviral defenses and abolishes the SA‑driven repellence to whiteflies. Using RNA‑seq and reverse genetics in N. benthamiana, they identify NbBT1 (BTB/POZ and TAZ domain‑containing protein 1) as an SA‑inducible gene and a positive regulator of SA signaling. Furthermore, the study shows that the BV1 downregulates NbBT1 transcription and, consequently, reduces the SA-responsive PR1a/PR2 expression as well as antiviral resistance and repellence to the vector. The novelty of the study is limited, as the viral suppression of SA signaling to attenuate antiviral defense and modify B. tabaci behavior has already been demonstrated by the same research group in other begomovirus systems. This includes the βC1–HSP90–NPR3‑mediated suppression of SA‑dependent immunity (Zhang et al., 2024) and the C2‑induced inhibition of SA/JA signaling that abolishes JA‑based repellence (Wang et al., 2026, First published: 16 January 2026 in Pest Manag Sci. 2026. doi: 10.1002/ps.70558.). In addition, SLCMV AC1 (Plant Stress 19, 2026, doi.org/10.1016/j.stress.2025.101134) was shown to activate anti‑whitefly defense via MYC2 accumulation/dimerization and TPS up‑regulation (a “negative relationship” with the vector), i.e., the opposite polarity of BV1.

Reviewer #2: The manuscript presents a comprehensive and mechanistically detailed study demonstrating how the bipartite begomoviral protein BV1 subverts SA signaling by downregulating NbBT1 to promote both viral infection and vector-mediated transmission. The work is compact with necessary validation demonstrated when and where required, and the use of multiple pathosystems, transgenic lines, and behavioral assays strengthens the conclusions. However, several gaps remain including missing controls (DNA-B alone, ethanol-treated BV1-transgenic RNA-seq), overclaimed causality (PAL pathway, DNA-B dependence), and incomplete datasets (NbBT1-overexpression repellence assays, silencing efficiency data) that require clarification or additional experimentation. With significant revisions addressing these points as suggested, the manuscript will be suitable for acceptance.

Reviewer #3: None

**********

**Part II – Major Issues: Key Experiments Required for Acceptance**

Please use this section to detail the key new experiments or modifications of existing experiments that should be absolutely required to validate study conclusions.

Generally, there should be no more than 3 such required experiments or major modifications for a "Major Revision" recommendation. If more than 3 experiments are necessary to validate the study conclusions, then you are encouraged to recommend "Reject".

Reviewer #1: I have major concerns:

The title and narrative suggest a direct impact on whitefly transmission; however, the data presented assess only insect preference/repellence (30‑min choice assays) and in‑plant viral accumulation. To substantiate such claims, the authors should include true transmission assays (acquisition/inoculation efficiencies) that causally link the BV1–BT1–SA axis to epidemiologically relevant outcomes.

The whitefly choice assays are based on only n = 5 cages and, critically, treat individual insects within a cage as independent observations despite the aggregative behavior of Bemisia. This constitutes pseudoreplication, as the experimental unit is the cage rather than each insect, resulting in inflated significance and reduced statistical power. I therefore recommend conducting single‑insect arena assays, or if group assays are maintained, analyzing data at the cage level with an adequate number of replicate cages and appropriate GLMM/beta‑binomial statistical models. Moreover, N. benthamiana is a suboptimal host for whitefly bioassays as its glandular trichomes secrete acylsugars that repel or physically trap adults, potentially confounding preference or settling measurements. Finally, given that salicylic acid modulates plant volatile emissions (e.g., methyl salicylate, D‑limonene) influencing B. tabaci host choice (Int. J. Mol. Sci. 2016, 17(7), 1048), it would be advisable to characterize the volatile organic compound (VOC) profile by GC–MS under ±SA and ±BV1/±BT1 conditions to complete the causal chain (plant → VOCs → vector).

The authors demonstrate that BV1 downregulates NbBT1 transcripts and that BT1 enhances SA signaling and whitefly repellence; however, the mechanism by which BV1 represses BT1 remains unclear. The molecular link downstream of BV1 needs to be elucidated. It is also noteworthy that the study focuses exclusively on BT1 without considering NbL03g16400, despite the potential for functional redundancy between these genes.

Reviewer #2: (No Response)

Reviewer #3: None

**********

**Part III – Minor Issues: Editorial and Data Presentation Modifications**

Please use this section for editorial suggestions as well as relatively minor modifications of existing data that would enhance clarity.

Reviewer #1: Please position your claim as a BV1‑specific addition to an already rich framework, and explicitly discuss AC1 vs BV1 antagonism and how these arms coexist temporally.

Deep functional genetics were conducted in N. benthamiana. Although the study presents assays in cassava (SLCMV) and zucchini (SLCCNV), functional data for BT1/BV1 in these natural hosts are missing. Identifying BT1 orthologs in cassava and zucchini, analyzing their expression levels (±SA and ±BV1), and performing VIGS—if feasible—would significantly strengthen the agronomic relevance of the findings.

Reviewer #2:

The manuscript presents a comprehensive and mechanistically detailed study demonstrating how the bipartite begomoviral protein BV1 subverts SA signaling by downregulating NbBT1 to promote both viral infection and vector-mediated transmission. The work is compact with necessary validation demonstrated when and where required, and the use of multiple pathosystems, transgenic lines, and behavioral assays strengthens the conclusions. However, several gaps remain including missing controls (DNA-B alone, ethanol-treated BV1-transgenic RNA-seq), overclaimed causality (PAL pathway, DNA-B dependence), and incomplete datasets (NbBT1-overexpression repellence assays, silencing efficiency data) that require clarification or additional experimentation. With significant revisions addressing these points as suggested, the manuscript will be suitable for acceptance.

INTRODUCTION

Lines 86-96 : The authors state that "whether and how the interplay between viruses and plant antiviral defenses modulates plant attractiveness to arthropod vectors remains largely unknown." This claim requires revision, as several studies have already established direct mechanistic links. For instance, Liang et al. (2025) demonstrated that TYLCV upregulates JA-associated terpene synthase genes in tomato; CRISPR knockout of these genes abolished both volatile emissions and whitefly attraction, proving that virus-modified defense gene expression directly controls vector preference. Similarly, Shi et al. (2019) used JA-pathway mutants to show that viruliferous whiteflies suppress JA biosynthesis in tomato, reducing defense volatiles and increasing plant attractiveness, explicitly linking viral modulation of phytohormone defenses to vector behaviour. The field has moved beyond asking whether such interplay exists to understanding how it is mechanistically executed. The authors should revise this sentence to reflect current knowledge while identifying genuine remaining gaps.

General Comment : More broadly, the Introduction section would benefit from restructuring. Rather than framing this research area as largely uncharted, the authors should first acknowledge and cite the growing body of mechanistic studies that have already established clear connections between virus-defense interplay and vector attractiveness. This would provide necessary context and demonstrate the authors' command of the field. Following this, the authors can then identify the specific gaps their work addresses such as unresolved molecular mechanisms, conserved signalling nodes, or host counter-manipulation strategies thereby positioning their contribution within an active, advancing research frontier rather than an unexplored one.

MATERIAL and METHODS

Lines 419-424, 432-435 ; Please provide details on the Agrobacterium strain used for generating the SLCMV BV1 and NbBT1 overexpression lines. For the NbBT1 CRISPR lines, please specify the Cas9 variant, the promoter driving Cas9 expression, and the method used to confirm homozygosity beyond Sanger sequencing.

Lines 477-484 ; Please provide more details on the PAL enzyme activity assay, including the full kit name, manufacturer, and catalogue number. Additionally, please clarify whether the assay was validated for the plant species tested (N. benthamiana, cassava, zucchini) and provide information on controls, background absorbance, and assay linearity.

Several commercial reagents are cited without catalogue numbers (e.g., Easy Plant Genomic DNA Extraction Kit, RNA extraction kit from Accurate Biology, Evo M-MLV RT Kit).

Lines 523-525; The authors state: "Apical leaves from three N. benthamiana plants were pooled as one biological sample." However, the total number of biological replicates per treatment for RNA-seq is not clearly stated. This information is essential for assessing the statistical power of the transcriptomic analysis.

Lines 513-521; Please provide additional details on the VIGS experiments: the specific region of NbBT1 targeted (e.g., 5'UTR, CDS, or 3'UTR), the time point post-infiltration when silencing was assessed, the tissues sampled, how silencing efficiency was evaluated and what criteria were used to define acceptable silencing, and the number of independent VIGS experiments performed.

Lines 550-557; Please provide details on whether data from independent experiments were pooled or if representative experiments are shown, specify the methods used to assess normality and homogeneity of variance prior to parametric testing, and indicate whether any corrections for multiple comparisons were applied.

RESULTS:

Lines 109-160 ; In the first result section "SLCMV induces SA accumulation via the PAL pathway in a DNA-B-dependent manner" :

The conclusion that SA induction is "DNA-B-dependent" is not fully supported, as DNA-B alone controls are missing. The current data only show that DNA-A+DNA-B induces more SA than DNA-A alone, not that DNA-B is sufficient or directly responsible. Please clarify whether DNA-B-only inoculations were attempted or revise the claim accordingly. The striking difference in SA accumulation between SLCMV (~37-fold) and other begomoviruses like TYLCV (~5-10 fold) is not addressed. Was viral titer comparable at the time of sampling? Were the same Agrobacterium strain and vector backbone used for all constructs? Please clarify whether differential infection efficiency or experimental conditions could explain this disparity. The claim that SA accumulation occurs "via the PAL pathway" remains correlational. PAL genes are upregulated and enzyme activity increases, but no loss-of-function evidence (PAL inhibitors, silencing) is provided to demonstrate causality. Please either temper this conclusion or provide additional support. Additionally, no true mock control (buffer-only or untransformed agrobacteria) is included. Since Agrobacterium infiltration itself triggers SA responses, this is a critical omission particularly when comparing across viruses.

Recommendation: Revise this subsection to reflect that successful systemic infection (requiring DNA-B) is associated with high SA and PAL activation, rather than claiming a direct, DNA-B-dependent, PAL-mediated mechanism.

Lines 162-201 ; In the second result section "SLCMV DNA-B and BV1 mitigate SA-induced antiviral defenses" ; There is an apparent contradiction regarding BV1's role: Figure 1G and lines 148-158 show that both BC1 and BV1 are required for SA accumulation, whereas lines 193-200 and Figure 2E-F show that BV1 does not affect SA levels but suppresses SA signaling. This creates confusion. Does BV1 contribute to inducing SA, suppressing its signaling, or both? The distinction is buried and should be explicitly clarified: BC1 and BV1 are likely required for systemic infection (thereby indirectly enabling SA induction), while BV1 alone directly suppresses SA signaling. Please revise the text to separate these two distinct functions and avoid any perceived contradiction.

Lines 203-225 ; In the section “SLCMV DNA-B and BV1 interfere with SA-induced plant repellence against whitefly” ; While this section is generally clear, one discrepancy should be addressed. In Figure 3C, the authors test whitefly preference on plants inoculated with SLCMV A, A+B, A+B-mBC1, and A+B-mBV1 following SA or ethanol treatment. However, no control is included for plants inoculated with DNA-B alone or DNA-B-mBC1/mBV1 alone (without DNA-A). This makes it difficult to determine whether the observed effects on repellence require active viral infection or whether BV1 expression alone is sufficient. Additionally, the order of treatments (inoculation first, then SA spray, then choice assay) is not clearly stated in the legend and should be explicitly described to aid interpretation.

Lines 227-254 ; In the section “SLCMV BV1 downregulates the transcription of BTB/POZ and TAZ domain-containing protein 1 (NbBT1), an SA-inducible gene” ;

Several discrepancies and missing controls should be addressed:

1. Missing control group: The RNA-seq experimental design (Figure 4A-F) compares SA-treated wild-type, SA-treated BV1-transgenic, and ethanol-treated wild-type plants. However, ethanol-treated BV1-transgenic plants were not included. This omission makes it impossible to determine whether BV1 downregulates NbBT1 independently of SA treatment or only in the presence of SA. Please explain why this control was omitted or provide additional data.

2. Figure 4G-H discrepancy: Figure 4G shows that SA induces NbBT1 transcription in a dose-dependent manner in wild-type plants. Figure 4H then shows that BV1 significantly reduces NbBT1 transcription under both ethanol and SA treatment. However, the ethanol-treated BV1-transgenic sample in Figure 4H has no counterpart in the RNA-seq analysis, creating inconsistency between the validation experiment and the transcriptomic dataset.

3. Incomplete validation: The authors identify two SA-responsive BT1 family genes (NbL03g16400 and NbL13g16600) from RNA-seq but only validate NbL13g16600 (NbBT1). No explanation is provided for why NbL03g16400 was not pursued, nor whether it shows similar regulation by BV1.

4. Discrepancy between FPKM and qPCR data: In Figure 4F, NbL13g16600 FPKM in SA-treated BV1-transgenic plants appears reduced compared to SA-treated wild-type but is still higher than ethanol-treated wild-type. In Figure 4H, qPCR shows BV1 reduces NbBT1 levels to below ethanol-treated wild-type baseline. This quantitative discrepancy should be addressed.

Please clarify these points and provide appropriate justification or additional data.

"NbBT1 positively regulates plant SA signaling, antiviral defenses and plant repellence against whitefly"

• Figure 5H shows whitefly preference for NbBT1-knockout over wild-type plants, but no corresponding assay with NbBT1-overexpression lines is shown. This omission leaves the gain-of-function evidence for repellence incomplete.

"NbBT1 is required for the suppression of SA signaling by SLCMV BV1"

• VIGS silencing efficiency data for NbBT1 are referenced but not shown anywhere in the main text or supplementary files. Please provide these data.

• The time point for PR gene expression analysis post-VIGS is not specified in the legend or methods.

"SLCCNV induces SA accumulation and SLCCNV BV1 interferes with SA signaling"

• Figure 6D uses SLCMV DNA-A as a proxy to test SLCCNV BV1 function in N. benthamiana. The authors should explicitly acknowledge this as a limitation, as the two BV1 proteins are not identical and may function differently.

• Figure 6I shows SLCCNV BV1 downregulates NbBT1, but no mechanistic data are provided to show this occurs via the same pathway as SLCMV BV1 ; this is assumed rather than demonstrated.

DISCUSSION

The discussion is well-structured but currently assumes all conclusions are fully validated and will require revision once the authors address the experimental gaps noted above. Specifically, the claim that SA accumulation is DNA-B-dependent should be tempered if DNA-B-only controls cannot be provided, and the assertion that SLCMV induces SA via the PAL pathway should be revised to reflect that causality has not been experimentally demonstrated. The discussion should also explicitly separate BV1's indirect role in enabling systemic infection from its direct role in suppressing SA signaling, as these are currently conflated. Additionally, the statement that BV1-mediated suppression is conserved across bipartite begomoviruses is based on only two viruses and should be softened to "observed in another bipartite begomovirus" unless additional evidence is provided. Finally, if missing controls or assays (such as ethanol-treated BV1-transgenic RNA-seq or NbBT1-overexpression repellence assays) are not added, the corresponding conclusions should be appropriately qualified. The authors should revisit the discussion after addressing these experimental gaps to ensure conclusions align with the strength of the evidence.

Reviewer #3: This paper investigates how a whitefly-transmitted begomovirus manipulates host plants and their defenses for their infection cycle and transmission. By investigating of two species of begomoviruses the authors show that plant infection with Sri Lankan cassava mosaic virus (SLCMV) induces the accumulation of salicylic acid SA, a positive regulator of antiviral defense. As a responses, SLCMV DNA-B and the BV1 protein interfere with SA-induced antiviral defenses and SA signal transduction. Additionally, SA induces plant repellence to whitefly vectors and this repellence is mitigated by SLCMV DNA-B and BV1. This response is achieved by BV1 downregulating the transcription of BTB/POZ and TAZ domain-containing protein 1 (BT1), a positive regulator of SA signal transduction, plant antiviral defenses and repellence against whitefly.

The manuscript show strong evidence for the arms race between virus infection and the plant defenses activated following infection. The manuscript is solid but would appreciate some clarifications:

1. The pharmacological treatment for manipulating the amount of SA using AIP. Are there any side effects on other molecular pathways that the authors are not aware about? How they can be sure about that? Is there maybe any effect on the virus itself? Can AIP be applied to other plant system to confirm this result?

2. In line 173 the authors assume that only SA functions in suppressing the ability of the virus to replicate and accumulate in the plant, while they didn't explore all possible mechanisms in the plant that might do that. The assumption that the virus might respond to the SA activity is OK, however they need to emphasize the fact that other unexplored mechanisms may exist.

3. In the section dealing with the BV1 interference with the SA pathway, the authors performed a transcriptomic analysis to identify gene regulated by BV1, and ended up with two BTB/POZ gene, one was chosen for further analysis and was regulated by BV1. My question is if the authors initially assumed that the SA would be regulated by BV1 and they were going for this direction, why they didn't focus on this group of genes, and went for full transcriptomic analysis?

**********

PLOS authors have the option to publish the peer review history of their article (what does this mean?). If published, this will include your full peer review and any attached files.

If you choose “no”, your identity will remain anonymous but your review may still be made public.

**Do you want your identity to be public for this peer review?**   For information about this choice, including consent withdrawal, please see our Privacy Policy.

Reviewer #1: No

Reviewer #2: No

Reviewer #3: No

[NOTE: If reviewer comments were submitted as an attachment file, they will be attached to this email and accessible via the submission site. Please log into your account, locate the manuscript record, and check for the action link "View Attachments". If this link does not appear, there are no attachment files.]

**Figure resubmission:**

-->While revising your submission, we strongly recommend that you use PLOS’s NAAS tool (https://ngplosjournals.pagemajik.ai/artanalysis) to test your figure files. NAAS can convert your figure files to the TIFF file type and meet basic requirements (such as print size, resolution), or provide you with a report on issues that do not meet our requirements and that NAAS cannot fix.

After uploading your figures to PLOS’s NAAS tool - https://ngplosjournals.pagemajik.ai/artanalysis, NAAS will process the files provided and display the results in the "Uploaded Files" section of the page as the processing is complete. If the uploaded figures meet our requirements (or NAAS is able to fix the files to meet our requirements), the figure will be marked as "fixed" above. If NAAS is unable to fix the files, a red "failed" label will appear above. When NAAS has confirmed that the figure files meet our requirements, please download the file via the download option, and include these NAAS processed figure files when submitting your revised manuscript.

**Reproducibility:**

To enhance the reproducibility of your results, we recommend that authors of applicable studies deposit laboratory protocols in protocols.io, where a protocol can be assigned its own identifier (DOI) such that it can be cited independently in the future. Additionally, PLOS ONE offers an option to publish peer-reviewed clinical study protocols. Read more information on sharing protocols at https://plos.org/protocols?utm_medium=editorial-email&utm_source=authorletters&utm_campaign=protocols

# Author comment: Author response to Decision Letter 1

**DOI:** 10.1371/journal.ppat.1014354.r002

3 Jun 2026

Attachment Submitted filename: 3 Point-to-point responses to comments PLL 2026.06.01.docx

# Editor report: Decision Letter 1

**DOI:** 10.1371/journal.ppat.1014354.r003

**Authors:** Shou-Wei Ding, John Carr, Shou-Wei Ding, John Carr

5 Jun 2026

Dear Dr pan,

Thank you for addressing the Reviewers' comments and revising your manuscript accordingly.

Although I am recommending acceptance (to avoid slowing down the publication process) I have noted quite a few minor issues (spelling, including in citation list, grammar) that I hope you will deal with carefully at the proofing stage.

We are pleased to inform you that your manuscript 'Subversion of the salicylic acid-signaling pathway by the bipartite begomoviral protein BV1 promotes virus infection and vector preference to virus-infected plants' has been provisionally accepted for publication in PLOS Pathogens.

Before your manuscript can be formally accepted you will need to complete some formatting changes, which you will receive in a follow up email. A member of our team will be in touch with a set of requests.

Please note that your manuscript will not be scheduled for publication until you have made the required changes, so a swift response is appreciated.

IMPORTANT: The editorial review process is now complete. PLOS will only permit corrections to spelling, formatting or significant scientific errors from this point onwards. Requests for major changes, or any which affect the scientific understanding of your work, will cause delays to the publication date of your manuscript.

Should you, your institution's press office or the journal office choose to press release your paper, you will automatically be opted out of early publication. We ask that you notify us now if you or your institution is planning to press release the article. All press must be co-ordinated with PLOS.

Thank you again for supporting Open Access publishing; we are looking forward to publishing your work in PLOS Pathogens.

Best regards,

John P Carr

Academic Editor

PLOS Pathogens

Shou-Wei Ding

Section Editor

PLOS Pathogens

Sumita Bhaduri-McIntosh

Editor-in-Chief

PLOS Pathogens

orcid.org/0000-0003-2946-9497

Michael Malim

Editor-in-Chief

PLOS Pathogens

orcid.org/0000-0002-7699-2064

***********************************************************

Reviewer Comments (if any, and for reference):

# Editor report: Acceptance letter

**DOI:** 10.1371/journal.ppat.1014354.r004

**Authors:** Shou-Wei Ding, John Carr, Shou-Wei Ding, John Carr

Dear Dr pan,

We are delighted to inform you that your manuscript, "Subversion of the salicylic acid-signaling pathway by the bipartite begomoviral protein BV1 promotes virus infection and vector preference to virus-infected plants," has been formally accepted for publication in PLOS Pathogens.

We have now passed your article onto the PLOS Production Department who will complete the rest of the pre-publication process. All authors will receive a confirmation email upon publication.

The corresponding author will soon be receiving a typeset proof for review, to ensure errors have not been introduced during production. Please review the PDF proof of your manuscript carefully, as this is the last chance to correct any scientific or type-setting errors. Please note that major changes, or those which affect the scientific understanding of the work, will likely cause delays to the publication date of your manuscript. Note: Proofs for Front Matter articles (Pearls, Reviews, Opinions, etc...) are generated on a different schedule and may not be made available as quickly.

Soon after your final files are uploaded, the early version of your manuscript, if you opted to have an early version of your article, will be published online. The date of the early version will be your article's publication date. The final article will be published to the same URL, and all versions of the paper will be accessible to readers.

For Research Articles, you will receive an invoice from PLOS for your publication fee after your manuscript has reached the completed accept phase. If you receive an email requesting payment before acceptance or for any other service, this may be a phishing scheme. Learn how to identify phishing emails and protect your accounts at https://explore.plos.org/phishing.

Thank you again for supporting open-access publishing; we are looking forward to publishing your work in PLOS Pathogens.

Best regards,

Sumita Bhaduri-McIntosh

Editor-in-Chief

PLOS Pathogens

orcid.org/0000-0003-2946-9497

Michael Malim

Editor-in-Chief

PLOS Pathogens

orcid.org/0000-0002-7699-2064
